# Early-season helping yields increasing returns to scale at the onset of eusociality

**DOI:** 10.1093/evlett/qraf033

**Published:** 2025-09-22

**Authors:** Viviana Di Pietro, Ricardo Caliari Oliveira, Tom Wenseleers

**Affiliations:** Department of Biology, Laboratory of Socioecology and Social Evolution, Zoological Institute, KU Leuven, Leuven, Belgium; Department of Biology, Laboratory of Socioecology and Social Evolution, Zoological Institute, KU Leuven, Leuven, Belgium; Departament de Biologia Animal, de Biologia Vegetal i d'Ecologia, Universitat Autònoma de Barcelona, Bellaterra (Barcelona), Spain; Department of Biology, Laboratory of Socioecology and Social Evolution, Zoological Institute, KU Leuven, Leuven, Belgium

**Keywords:** eusociality, social evolution, demographic benefits, monogamy, population genetic model

## Abstract

The evolution of eusociality, characterized by cooperative brood care, reproductive division of labor, and overlapping generations, represents a major evolutionary transition. A central paradox is that early models suggested diminishing returns to helping, making solitary reproduction seemingly more efficient. Here, we experimentally demonstrate increasing returns to scale from early-season helping in the primitively eusocial wasp *Polistes gallicus*. By manipulating the proportion of females allowed to remain as helpers, we show that while reproductive output scales linearly with worker numbers, total sexual productivity increases convexly with helper probability, thereby showing that early-season helping leads to compounding effects on reproductive output. Using these data, we parameterize a dynamic population genetic model and show that this convex relationship facilitates the spread of eusociality alleles more readily under monandry than polyandry, contrary to the conclusions of some prior models. Importantly, we show that compounding effects can cause eusociality to evolve even when helpers are no more efficient at rearing brood than solitary breeders. Our findings emphasize the value of integrating experimental data with mechanistically motivated theoretical models to study social evolution.

## Teaser

Why complex animal societies, like those of ants and bees, evolved has long been debated, especially since early theories suggested that helping was less efficient than breeding alone. We show experimentally in wasps that even a small initial commitment to helping by offspring yields disproportionately large reproductive gains for the colony over time. A mathematical model shows that these increasing returns of helping arise from compounding effects of early helping and cause sociality to evolve most readily when workers help close relatives. This work clarifies how demographic benefits of early cooperation can cause eusociality to evolve, thereby providing a new perspective on a major evolutionary transition and resolving a long-standing paradox.

## Introduction

The evolution of eusociality, defined as a social system with cooperative brood care, reproductive division of labor, and overlapping generations, represents one of the most significant transitions in the history of life (Boomsma, 2022; Bourke, 2011). Much work has focused on explaining the emergence of reproductive altruism, whereby some individuals forgo personal reproduction to help others. Inclusive fitness theory and Hamilton’s rule (Hamilton, 1963, 1964) posit that helping can spread if indirect fitness benefits to kin outweigh direct fitness costs (Boomsma, 2022; Bourke, 2011, 2014; Crozier et al., 1996). In a further elaboration of this, the “monogamy hypothesis” posits that strict lifetime monandry was in fact a critical prerequisite for eusociality to evolve, as it makes the relatedness to sibs equal to mother-offspring relatedness, so that even modest demographic benefits of group living would favor eusociality (Boomsma, 2007, 2009, 2013).

Although inclusive fitness theory has been immensely influential and remains an extremely powerful framework to study long-term evolution (Abbot et al., 2011; Hughes et al., 2008), most models have been derived in the limit of vanishingly weak selection (Boomsma, 2009, 2013; Crozier et al., 1996; Frank, 1998; Gadagkar, 1990, 1991; Queller, 1989; Quiñones et al., 2017; Rees-Baylis et al., 2024). This assumption is appropriate for very large populations, but in smaller populations, such as those typical for primitively social Hymenoptera (where effective population sizes can be as low as 100), genetic drift can cause stochastic loss of weakly selected mutations, and mutations of moderate or large effect may instead play a decisive role (Allen et al., 2016; Birch, 2017). Moreover, many existing models are not mechanistically motivated. That is, they do not explicitly model how particular costs and benefits of helping would arise from underlying colony dynamics, such as worker-accelerated brood production or seasonal demography (Charlesworth, 1978; Charnov, 1978; Craig, 1979; Davies et al., 2018; Eshel et al., 2001; Levitt, 1975; Olejarz et al., 2015; Pamilo, 1984; Williams et al., 1957). Instead, they typically assume abstract cost–benefit trade-offs or static fitness functions, without capturing how helping affects colony growth and the cumulative production of reproductives across the season. As a result, these models often fail to elucidate what ecological or demographic conditions would produce particular costs and benefits of helping. In addition, even models with greater mechanistic structure often remain empirically unvalidated (Fromhage et al., 2011; Fu et al., 2015; Liao et al., 2015; Nowak et al., 2010), while others neglect critical details of the genetic system such as haplodiploidy, dominance relationships of invading alleles, or the timing and penetrance of gene expression, despite all of these influencing the potential for helping to invade in structured populations (e.g., Field et al., 2020; Fu et al., 2015, assumed asexual reproduction instead of haplodiploidy). These considerations motivate the need for models that are both mechanistically informed and empirically grounded, while remaining flexible enough to accommodate different strengths of selection and genetic systems. Such models complement existing approaches and help clarify the evolutionary conditions under which eusociality can emerge, especially in small, structured populations where demographic feedbacks and stochasticity may play a central role (Fromhage et al., 2011; Gadagkar, 1990, 1991; Nonacs, 2019; Queller, 1989).

Lately, attention has shifted to how the functional relationship between colony productivity and the proportion of helpers determines the conditions for the evolution of social behavior. Early models assumed linear colony-level benefits of helping (Williams et al., 1957), but more recently, Olejarz et al. (2015) demonstrated that if productivity is a convex function of the proportion of offspring that help—so that even a small increase in helper fraction produces a large gain—altruistic helping may evolve more readily at low relatedness. Although Davies et al. (2018) noted that this model requires mutations of large effect to work and was derived in the context of the evolution of worker sterility, the logic could also apply to eusociality itself. The idea is that under polyandry, it becomes more likely that at least one father would carry a rare eusociality allele, leading to relatively more sibships with at least some helpers, thereby allowing the allele to invade more easily if such sibships were sufficiently productive (Olejarz et al., 2015). Equally, alternative scenarios have been proposed that do not always require obligate monogamy, e.g., via maternal manipulation of offspring (Rees-Baylis et al., 2024) or a temporary nonaltruistic stage with delayed direct fitness benefits linked to nest inheritance (Nonacs, 2011).

Empirical studies on the ergonomic efficiency of social insect colonies—defined as the rate at which colonies convert a given number of workers into reproductive output—have predominantly relied on static, correlative analyses. These typically relate either cumulative worker number to final reproductive output (Jeanne et al., 2022) or current worker number to the rate of brood or adult emergence (Field et al., 2020; Kennedy et al., 2021). In an influential early study, Michener (1964) argued that brood-rearing efficiency declines with increasing group size, which led to a longstanding paradox: If solitary females achieve the highest per-capita efficiency, what selective forces could favor social behavior? Recent meta-analyses have challenged this view by showing that per-capita brood-rearing efficiency is generally independent of colony size across species, implying linear productivity benefits of helping (Jeanne et al., 2022). And although some primitively eusocial species exhibit slight declines in per-capita efficiency as colony size increases—particularly in cases where multiple foundresses that both help and compete are present (Clouse, 2001; Jeanne et al., 2022; Shreeves et al., 2002, 2003)—these declines do not necessarily translate into lower fitness benefits. This is because when additional advantages such as improved nest defense or reduced brood mortality are taken into account, the net returns on helping can remain constant or even increase (Brand et al., 2014; Field et al., 2020; Tibbetts et al., 2003). For example, single worker-removal experiments in a halictid bee demonstrated strong nest-defense benefits (Brand et al., 2014), and re-analysis of these data reveals that per-capita fitness returns on helping are stable or increasing with colony size, even though larger colonies are less efficient at brood provisioning.

Despite decades of research on ergonomic efficiency and returns on helping, no study to date has measured how reproductive output would have scaled with helping at the origin of eusociality—when the evolving trait was the *probability of offspring to help*, rather than *absolute worker number*. This is remarkable, given that it is this functional relationship that critically affects theoretical predictions (Olejarz et al., 2015; Williams et al., 1957). In addition, earlier empirical work has not accounted for delayed ergonomic benefits arising from workers facilitating the production of additional workers. Such delayed, compounding demographic benefits could produce a convex relationship between the probability of early helping and end-of-season sexual output (Bulmer, 1994; Hovestadt et al., 2018; Oster et al., 1978).

Here, we address this gap by experimentally simulating incipient sociality in the primitively eusocial paper wasp *Polistes gallicus*. By varying the proportion of early-emerging females allowed to remain as helpers, we quantify how total sexual production scales with the probability of staying as a helper. Our results reveal that although sexual productivity increases linearly with cumulative worker numbers, the relationship between the probability of staying as a helper and total reproductive output is convex. This implies that early-season helper contributions yield increasing returns due to compounding demographic gains. We then used these empirical data, complemented by independently estimated life-history parameters for *Polistes* from previous studies (Southon et al., 2015; Strassmann et al., 2003), to parameterize a fully dynamic population-genetic model, valid under arbitrary strength of selection (Birch, 2017; Gardner et al., 2011; Lehmann et al., 2020; Wenseleers et al., 2010). Counter to earlier arguments (Olejarz et al., 2015), we show that polyandry only permits invasion of eusociality under unrealistically high productivity, aligning with the empirical prevalence of ancestral monogamy in social Hymenoptera (Hughes et al., 2008). Furthermore, we demonstrate that eusociality alleles can spread under monandry even when helpers are no more efficient than solitary breeders, provided that the timing of helper behavior is such that it confers significant demographic advantages.

Taken together, our findings offer a mechanistically informed perspective on the origin of eusociality. By integrating experimental work with a dynamic population-genetic framework, we show how early-season helper contributions, together with monogamy, can drive the evolution of complex social systems, even when traditional per-capita productivity metrics appear constant. This work thus provides new insights into the genetic, ecological, and demographic factors underpinning one of the most significant transitions in evolutionary history, and offers an alternative resolution to Michener’s long-standing paradox (Jeanne et al., 2022; Michener, 1964).

## Methods

### Experimental simulation of incipient sociality

To assess how reproductive output scales with helping effort at the origin of eusociality, we experimentally simulated incipient sociality in the primitively eusocial paper wasp *P. gallicus*. While previous studies have sometimes examined facultatively social species (Bourke, 2014; Kapheim et al., 2011, 2015), the demographic parameters measured in such species are not representative for lineages that evolved obligate eusociality, because if they were, they too should already have evolved obligate sociality. To overcome this limitation, we instead focus on an obligate but still primitively eusocial species, *P. gallicus*. Although *Polistes* paper wasps have been eusocial for ca. 50 million years (Peters et al., 2017), their primitively eusocial colony structure that lacks morphologically defined female castes provides a tractable system to approximate ancestral conditions, provided that some parameters, such as sex ratio patterns, are adapted to reflect likely values in original solitary breeding ancestors (Table 1).

**Table 1.** Parameters used in our model, inspired by the lifecycle of the annual primitively eusocial paper wasp *Polistes gallicus* and our experimental data (*n* = 12 colonies).

Parameter	Value [95% C.I.s]	Meaning	Reference
*L*	97^a^	Length of the season in days after which no more new individuals are produced	This study
*q*	0.71^b^ (derived situation), 0.5 (ancestral situation)	Proportion of the season after which all females that are produced disperse and mate	This study
*f_1_*	1 (derived situation), 0.5^c^ (ancestral situation)	Proportion of individuals produced before time *q.L* that are females	This study
*f_2_*	0.54^d^ (derived situation), 0.5^c^ (ancestral situation)	Proportion of individuals produced after time *q.L* that are females	This study
*b*	0.094^e^	Per-capita productivity (birth rate, number of new individuals produced per individual per day)	This study
*μ*	1/19.2^f^ = 0.052	Mortality rate of workers (per day)	Southon et al. (2015)
*ν*	1/(19.2 × 5.8^g^) = 0.009	Mortality rate of sexuals (per day)	Southon et al. (2015)
*m_e_*	1, 2, or 3^h^	Effective female mating frequency	Strassmann et al. (2003)

a Adding one generation (39 days [Pardi, 1951]) for the few workers that were already present when observations started.

b Sexuals (males and dispersing females) started to be produced on average 30 days after the start of our observations ([23–38] 95% C.I. days, marginal mean from a Gamma GLM with treatment coded as a factor), which counting from nest initiation gives *q* = (30 + 39)/97 = 0.71. There were no significant differences in the onset of sexual production across treatments (type III ANOVA, *F*_2,9_ = 0.079, *p* = 0.92).

c Ancestrally, an identical and equal investment sex ratio across the season is assumed, in line with the expected maternal optimum.

d Fitted from a binomial GLMM with colony coded as a random intercept ([0.49–0.60] 95% C.I.s).

e Best fitting value of *b* ([0.092–0.096] 95% C.I.s) obtained using a nonlinear Poisson maximum likelihood fit, based on the solution of our ODE model; for details, see Figure 1.

f Reciprocal of the average worker life expectancy reported for *P. gallicus* (Southon et al., 2015).

g Based on foundress life expectancy that is reported to be 5.8 times longer than the worker life expectancy across five species of *Polistes* (Southon et al., 2015). For simplicity, the mortality rate of male and female sexuals is assumed to be identical.

h *Polistes gallicus* females normally mate with a single male (Strassmann et al., 2003), but as counterfactual scenarios, we also consider double or treble mating.

Twenty single-foundress nests were collected from late June 2022 on the campus of the Universitat Autònoma de Barcelona in Bellaterra, Spain (41°30′03.3″N, 2°06′26.9″E), and monitored until mid-September 2022. Nests were initiated by single, overwintered foundresses and were collected when the first workers were about to eclose or had just eclosed. Nests were randomly assigned to one of three experimental treatment groups. In these groups, approximately 0% (control, *n* = 6), 25% (*n* = 8), or 50% (*n* = 6) of all newly eclosed workers were removed throughout the ergonomic growth stage of the colony, i.e., before the onset of sexual production (Figure 1). This manipulation aimed to vary the proportion of female offspring that effectively stayed as helpers. Each nest was visited every other day. Newly emerged workers were individually marked on the thorax with nontoxic, oil-based acrylic markers (Uniball Paint Marker PX-21) for individual recognition. The original mated foundress in each nest was marked with a distinctive pink color to facilitate recognition and ensure she was alive and present.

**Figure 1.**
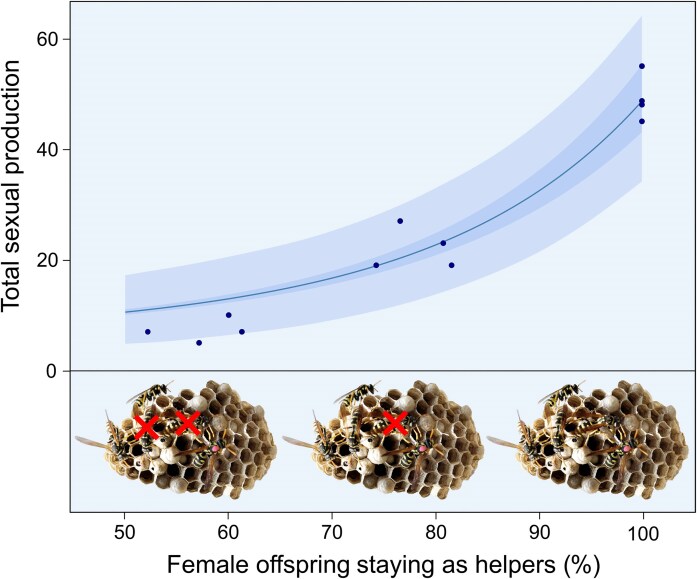
Helping confers increasing returns to scale in the annual paper wasp *Polistes gallicus*. By experimentally removing either ca. 50%, 25%, or none of the newly eclosed workers throughout the early season, before dispersing sexuals were produced, we simulated a variable fraction of females either staying and helping or leaving the nest to mate and breed on their own. In this way, we were able to mimic incipient sociality and determine how total sexual production measured at the end of the season would scale in function of the proportion of females carrying a dominant eusociality allele encoding helper behavior. Results show that this relationship is convex, i.e., there are increasing returns to scale of helper behavior, and that it is consistent with the solution of our ODE system whereby the workers and foundress have additive effects on the birth rate of new individuals throughout the colony cycle. The line represents a nonlinear Poisson maximum likelihood fit of the cumulative female and male sexuals produced, i.e., of *T_f_* + *T_m_* at the end of the season (*t* = *L*), whereby the per-capita birth rate *b* was fitted using penetrance parameter *P* = 1 and the remaining parameters were set as in Table 1 (*b* = 0.094, [0.092–0.096] 95% CL, *n* = 12 colonies); dark and light shaded areas represent 95% confidence and prediction intervals. All nests contained a single foundress, which was paint-marked to facilitate recognition. For details, see R script in Dataset S2.

The transition from the production of workers to sexuals was relatively abrupt. We used the appearance of the first male in a colony as a reliable marker for the onset of sexual production, as reproductives of both sexes are typically produced simultaneously in *P. gallicus* (Suzuki, 1986). Upon the appearance of the first male, all subsequently emerging individuals (both males and females) were collected and frozen at –20 °C. These individuals were later dissected to verify that the females emerging at the end of the season were indeed gynes (future queens), identifiable by their larger, solid white fat bodies characteristic of diapausing reproductives (Strassmann, 1984). In addition, we visually inspected the spermatheca of each foundress to ensure that each nest contained only one mated female. The experiment was terminated for each colony once its lifecycle was completed, typically indicated by the cessation of egg-laying, emergence of all brood, and the death or disappearance of all adult wasps. Out of the 20 study colonies, 12 (four per treatment condition) survived to produce dispersing sexuals and were included in the analyses of reproductive output.

### Statistical analysis

We evaluated the effect of treatment (categorical) or the proportion of females staying as helpers (parameter *p*, continuous) on the first day of sexual emergence using Gamma generalized linear models (GLMs) with a log link. These analyses showed no significant relationship between the onset of sexual production and worker removal treatment; instead, sexuals appeared to be produced after a fixed fraction of the season had passed. Nest failure (i.e., failure to produce dispersing sexuals) was analyzed using a binomial GLM with a logit link, using treatment as an explanatory variable. The numerical sex ratio among dispersing sexuals (proportion female, parameter *f_2_*​ in Table 1) was estimated from the data using a binomial GLM. Generalized linear mixed models (GLMMs) with colony ID added as a random intercept were also fitted, but in all cases resulted in the variance for the random effect being estimated as zero or near-zero and resulted in models with a worse AICc (Akaike Information Criterion), thereby implying there was no evidence for overdispersion caused by differences in nest locality or foundress quality.

To test how reproductive efficiency varied as a function of the cumulative number of workers produced over the colony lifecycle, we fitted linear and quadratic least-squares models, following approaches similar to Jeanne et al. (2022). For total sexual production, gyne, and male production (response variables), linear models provided a more parsimonious fit than quadratic models based on the AICc corrected for small sample sizes. Furthermore, log–log Poisson GLMs (log link) of sexual, gyne, and male production versus the log of cumulative worker numbers confirmed that the regression slopes were not significantly different from 1, indicating that per-capita brood-rearing efficiency (*b*) was independent of colony size. Here too, GLMMs with colony ID added as a random intercept were fitted, but were less parsimonious as there was no evidence for any overdispersion.

To assess how cumulative worker production scaled with the proportion of offspring staying as helpers (*p*, continuous), we fitted a log-link Poisson GLM. This model was strongly favored over a zero-intercept linear model (ΔAICc = 11.02), indicating a log-linear relationship between cumulative worker production and *p*. Finally, to quantify the nonlinear relationship between total sexual production and *p* in more detail, we fitted the solution of our Ordinary Differential Equation (ODE) model (see below) to the experimental data using a nonlinear Poisson maximum likelihood fit. In this fit, the per-capita birth rate parameter *b* was treated as a free parameter, while the remaining parameters were set to their empirically estimated or known values (Figure 1, Table 1). This nonlinear model provided a more parsimonious fit than alternative zero-intercept linear (ΔAICc = 62.05, evidence ratio = 3.10^13^) or softplus-smoothed broken-stick Poisson regressions (ΔAICc = 2.53, evidence ratio = 3.55; Dataset S2). To examine the fitness returns on helping in the function of group size in another primitively social species, the sweat bee *Halictus scabiosae*, we re-analyzed earlier data (Brand et al., 2014) on sexual productivity (counts of total second generation brood) in function of helper number (either counting the foundress mother as a helper or not) across all nests, including ones that eventually failed, using log–log zero-inflated negative binomial hurdle models (Dataset S1).

All statistical analyses were performed in R version 4.4.1. Linear, generalized linear, and GLMMs were fitted using the *lm, glm*, and *glmer* functions. Nonlinear maximum likelihood fits were performed using the *mle2* function in the *bbmle* package. Marginal means or marginal trends post hoc tests were performed using the *emmeans* package. Re-analysis of data on *H. scabiosae* used zero-inflated negative binomial hurdle models via the *pscl* package (Dataset S1).

### A dynamic population genetic model for the evolution of eusociality

**Figure 2.**
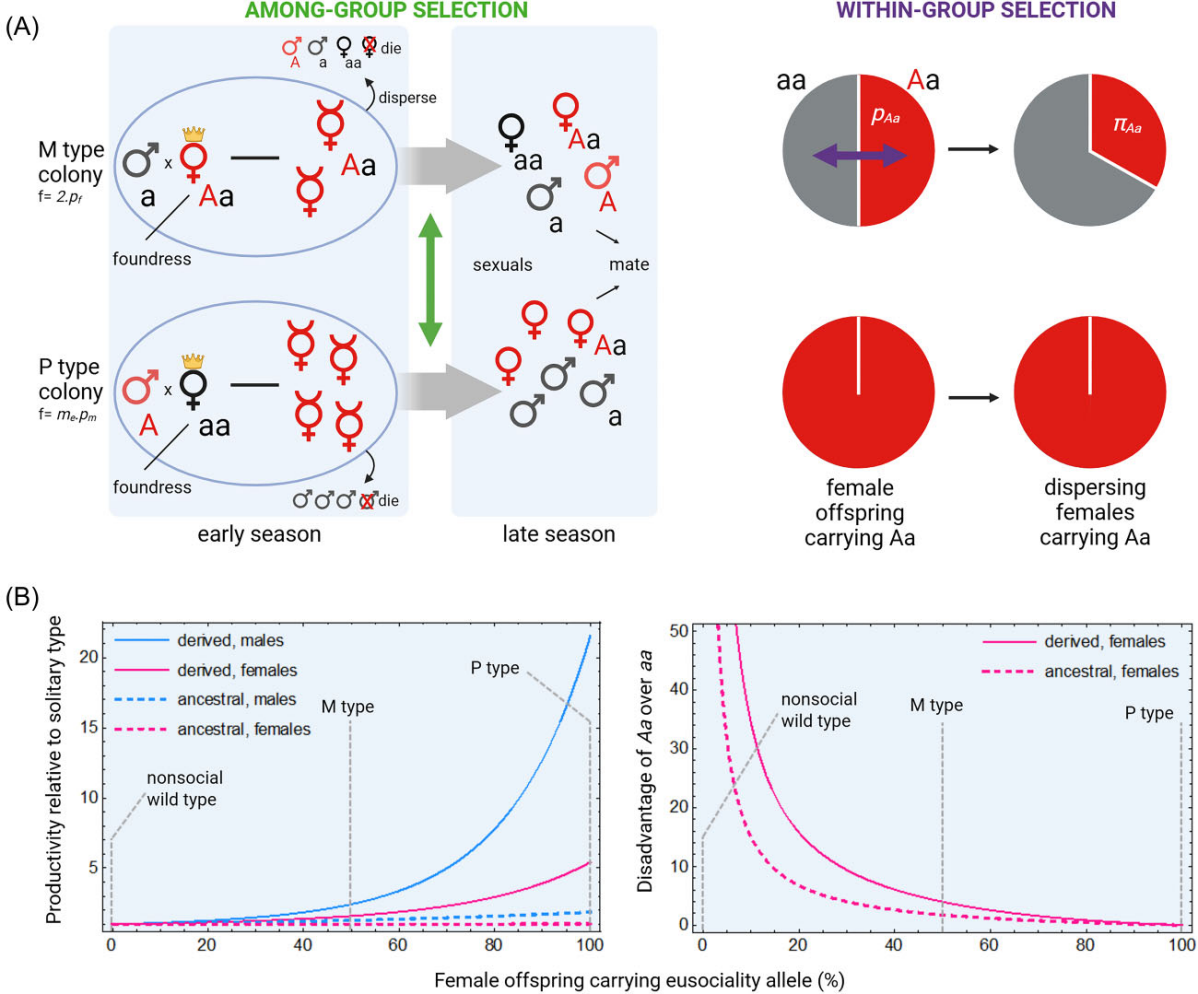
Opposing among-group and within-group selection for eusociality in male-haploid populations. (A) A rare, dominant eusociality allele (*A*) can arise through maternal (M-type sibships, frequency ≅ *2.p_f_*) or paternal inheritance (P-type sibships, frequency ≅ *m_e_.p_m_*, where *m_e_* is the effective mating frequency). In P-type sibships, all daughters carry the *A* allele, while in M-type, only half do. In our model, *Aa* females carrying the *A* allele may remain as helpers early in the season with a probability determined by penetrance (*P*), thereby boosting colony productivity. This creates among-group selection: Colonies with more helpers (e.g., P-type colonies under monandry) are more productive and produce more dispersing sexuals. However, this benefit is counterbalanced by within-group selection, as *Aa* females that help, forgo direct reproduction and leave fewer personal descendants than *aa* females in the same colony (Figure created in BioRender, Di Pietro, 2025). (B) Model predictions show how reproductive output scales with helping effort. The left panel shows the relative productivity benefits (*G_m_* and *G_f_*) in male and female offspring (compared to a nonsocial wild type) due to increased helping. The right panel shows the relative fitness disadvantage of *Aa* females over *aa* females within colonies (*T_faa_*/*T_fAa_* = (1 − *π_Aa_*)/*π_Aa_*). As more females carry the *A* allele and become helpers, their relative direct fitness declines, explaining the steep drop in their within-group reproductive share. Results are shown for an ancestral scenario (equal investment in females before and after onset of sexual production; *f₁* = *f₂* = 0.5, switchpoint *q *= 0.5) and a derived scenario based on *Polistes gallicus* life history (*f₁* = *1, f₂* = 0.54, *q * = 0.71, Table 1). For details, see Mathematica notebook in Dataset S3.

While our experiments focus on *P. gallicus*, chosen for its tractable and ancestrally representative colony structure, the model is intended to explore general invasion conditions for eusociality in annual haplodiploid species. In particular, the ancestral scenario modeled (Table 1) allows early helpers to produce additional workers before the production of sexuals, creating compounding demographic benefits and convex returns on helping. This framework generalizes beyond *Polistes* and is applicable to lineages with extended or multivoltine cycles, such as vespine wasps, where multiple broods of workers precede the production of dispersing sexuals (Bulmer, 1994; Hovestadt et al., 2018). We developed a dynamic population genetic model to investigate invasion conditions for a rare, dominant eusociality allele (*A*) in an annual, haplodiploid species, empirically parameterized from our *P. gallicus* data (Figure 2, Dataset S3). The allele, expressed with penetrance *P* in female carriers early in the season (before fraction *q* of season *L*), causes them to stay and help. We assume that a mutation preventing females from dispersing also results in helping behavior, based on evidence that worker behavior was likely co-opted from preexisting gene regulatory networks encoding maternal care (Amdam et al., 2006; Linksvayer et al., 2005; Rehan et al., 2014; Toth et al., 2007). As ancestral solitary Hymenoptera typically display maternal care, we assume helping behavior is restricted to females (Ross et al., 2013). The model comprises an ODE system for within-season colony demographics and recurrence equations for across-year allele transmission, assuming an annual lifecycle with synchronous mating (see the Online Appendix).

For the first part of the season (before switchpoint *t* = *q.L*), we use


(1) \begin{eqnarray*}
F^{\prime}\left( t \right)&=& 0 \\
W^{\prime}\left( t \right) &=& b.{{f}_1}.{{p}_{Aa}}.P.\left( {W\left( t \right) + F\left( t \right)} \right) - \mu .W\left( t \right)\\
W_{\mathrm{ cum}}^{\mathrm{^{\prime}}}\left( t \right) &=& b.{{f}_1}.{{p}_{Aa}}.P.\left( {W\left( t \right) + F\left( t \right)} \right)\\
Q_{aa}^{\mathrm{^{\prime}}}\left( t \right) &=& b.{{f}_1}.\left( {1 - {{p}_{Aa}}} \right).\left( {W\left( t \right) + F\left( t \right)} \right) - \nu .{{Q}_{aa}}\left( t \right) \\
Q_{Aa}^{\mathrm{^{\prime}}}\left( t \right) &=& b.{{f}_1}.{{p}_{Aa}}.P.\left( {W\left( t \right) + F\left( t \right)} \right) - \nu .{{Q}_{Aa}}\left( t \right)\\
M^{\prime}\left( t \right) &=& b.\left( {1 - {{f}_1}} \right).\left( {W\left( t \right) + F\left( t \right)} \right) - \nu .M\left( t \right),
\end{eqnarray*}

while dynamics in the second part of the season (after switchpoint *t* = *q.L*) can be described by


(2) \begin{eqnarray*}
F^{\prime}\left( t \right) &=& 0\\
W^{\prime}\left( t \right) &=& - \mu .W\left( t \right)\\
Q_{aa}^{\mathrm{^{\prime}}}\left( t \right) &=& b.{{f}_2}.\left( {1 - {{p}_{Aa}}} \right).\left( {W\left( t \right) + F\left( t \right)} \right) - \nu .{{Q}_{aa}}\left( t \right)\\
Q_{Aa}^{\mathrm{^{\prime}}}\left( t \right) &=& b.{{f}_2}.{{p}_{Aa}}.\left( {W\left( t \right) + F\left( t \right)} \right) - \nu .{{Q}_{Aa}}\left( t \right)\\
M^{\prime}\left( t \right) &=& b.\left( {1 - {{f}_2}} \right).\left( {W\left( t \right) + F\left( t \right)} \right) - \nu .M\left( t \right),
\end{eqnarray*}

where *F, W, W*_cum_, *Q_aa_, Q_Aa_*, and *M* are compartments for the foundress mother, workers, the cumulative number of workers produced and *aa* and *Aa* dispersing female sexuals (gynes) and males; *b* is the per-capita productivity (assumed independent of colony size, in line with our *P. gallicus* data and a large meta-analysis of data from other species; Jeanne et al., 2022); *f_1_* and *f_2_* are the numerical allocation to females before or after switchpoint *t* = *q.L; µ* is the mortality rate of workers (also assumed independent of colony size); and *ν* is the mortality rate of dispersing sexuals (here assumed identical for both sexes). As initial conditions, we set *F*(0) = 1 and 0 for the remaining compartments.

Key assumptions for the ODE model (tracking foundress, workers, and sexuals) include (i) additive per-capita productivity *b* from foundress and workers; (ii) a fixed switch (*q.L*) to sexual production independent of worker numbers; (iii) foundress mortality and subsequent nest inheritance (Leadbeater et al., 2011; Nonacs, 2011) or worker reproduction (Strassmann et al., 2003) are ignored to be able to focus on true worker altruism; (iv) producing new workers and dispersing females and males are all assumed to be equally costly; (v) effects of worker addition on colony growth are instantaneous; and (vi) nest failure is concentrated in the nest founding stage.

The ODE system yields end-of-season sexual production for different helper proportions. These outputs are then used to set up recurrence equations, which model cross-year allele *A* transmission via maternally (M-type) or paternally (P-type) inherited alleles, given a particular effective queen mating frequency *m_e_*​ (see the Online Appendix). The dominant eigenvalue of the resulting gene flow matrix determines the selection differential (*S*) for allele *A*. Invasion requires *S* > 0, or if we consider drift in small populations, *S* > 1/*N_e_*​. Ancestral conditions (*f_1_* = *f_2_* = 0.5, *q *= 0.5) were assumed for initial invasion modeling (Dataset S3).

## Results

### Increasing returns to scale of helping

To simulate incipient sociality at the origin of eusociality, we experimentally varied the proportion of newly eclosed *P. gallicus* females allowed to remain as helpers in single-foundress nests, continuously removing 0%, ∼25%, or ∼50% of the workers, before the onset of sexual production. The transition from the production of workers (nondispersing females) to sexuals (dispersing females with more developed fat bodies and males) was relatively abrupt, and its onset did not differ significantly between our treatments (Gamma GLM, Figure S1 and Dataset S1). This is consistent with the onset of sexual production occurring at a fixed fraction of the season’s length *L* (*q* = 0.71, Table 1)—approximately one generation before the end of the season (Hovestadt et al., 2018)—as opposed to when a critical colony size was reached, as assumed by Fromhage et al. (2011).

Out of our 20 study colonies, 12 survived to produce dispersing sexuals. We found that the cumulative total sexual output (males and dispersing females) at the end of the season exhibited a clear convex relationship with increasing proportion of females that were allowed to stay and help (Figure 1). This demonstrates increasing returns to scale on the *probability* of early helping. Although our results are for successful colonies only, excluding failed nests does not bias our results, since nest failure occurred only at or soon after nest founding and, based on our data, showed no differences across our worker removal treatments (binomial GLM, ANOVA test, $\chi _2^2\ $= 0.55, *p *= 0.76, Figure S2). Key life-history parameters observed in our experiment or derived from literature (Table 1) were subsequently used to parameterize our theoretical model.

### Ergonomic efficiency is independent of colony size

The observed increasing returns to scale of early helping probability (Figure 1) occur despite the per-capita brood-rearing efficiency, as such, being constant and independent of colony size. This was evidenced by a linear effect of the cumulative total number of workers on total sexual production when measured over the entire colony lifecycle (log-link Poisson GLMs, all *p* > 0.05, Figure S3). This finding is consistent with recent meta-analyses suggesting that per-capita brood-rearing efficiency is typically independent of colony size across most social insect groups (Jeanne et al., 2022). It also matches earlier findings on linear productivity benefits (Field et al., 2020), including *Polistes* paper wasps when measured over the entire colony lifecycle, such as in *P. snelleni* (Inagawa et al., 2001) and *P. chinensis* (Suzuki, 1981).

### Delayed ergonomic benefits of sociality

The convex relationship between helping probability and reproductive output (Figure 1) arises from delayed ergonomic benefits: workers produced early in the season help to produce more workers before the onset of sexual production (Bulmer, 1994; Hovestadt et al., 2018; Oster et al., 1978). Our empirical data show a compounding effect of early helping on cumulative worker numbers, resulting in a log-linear relationship between the proportion of offspring that stay as helpers and the total number of workers produced (Figure S4). To formalize this, we developed an ODE model of within-season colony growth based on key empirical observations. It assumes that both the foundress and each helper contribute additively to the colony’s net birth rate (with contribution *b*), and that critically, prior to the switch to sexual production (at observed fraction *q* = 0.71 of the season), all newly produced individuals are females that remain to help (i.e., numerical investment in females, *f_1_* ​= 1). When the ODE model, incorporating these worker-compounding dynamics and estimated parameters from Table 1, was fitted to our experimental data, it accurately replicated the observed convex relationship between helping probability and total sexual output (Figure S5 and Figure 1, blue line; estimated *b* = 0.094, [0.092–0.096] 95% C.I.s). This confirms that the benefits of early helping cascade and compound over time, leading to the observed increasing returns to scale.

### Increasing returns to scale reinforce traditional predictions

The experimentally observed convex relationship (Figure 1; Figure 2B, derived state) indicates increasing returns to scale of helping, contrasting with some theoretical hypotheses of concave returns that could favor eusociality under low relatedness (e.g., polyandry) (Olejarz et al., 2015). Such hypotheses often relied on polyandry increasing the chance that at least one father carries a rare eusociality allele, boosting productivity in P-type sibships if even a few helpers provide large benefits (Olejarz et al., 2015). However, given our empirical finding of convex or near-linear returns, this specific argument for polyandry facilitating invasion is less likely to apply to the initial evolution of eusociality.

**Figure 3.**
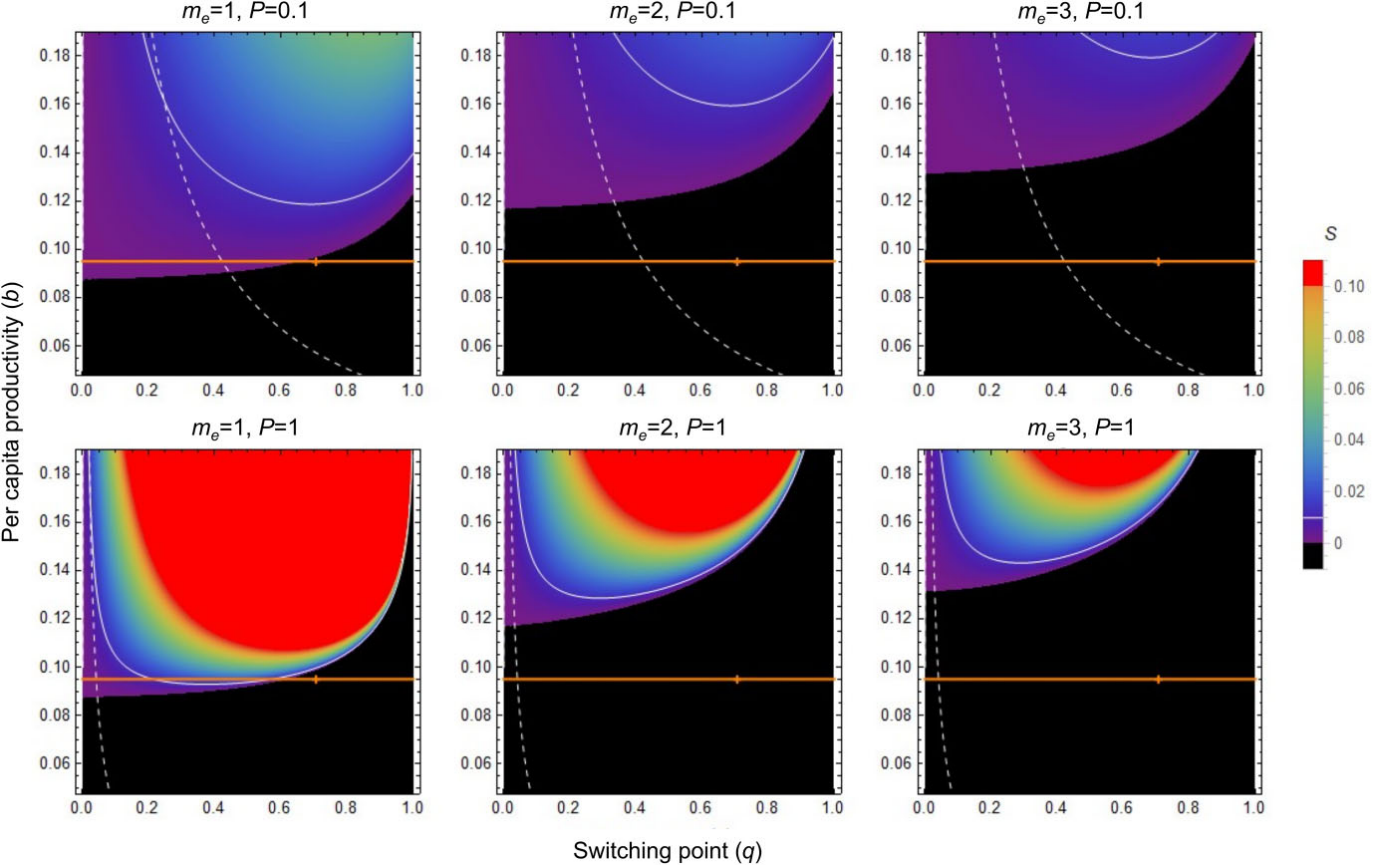
Genetic and demographic conditions favoring eusociality in annual haplodiploid populations. As expected, if there is a convex relationship between reproductive output and helping effort (Figure 2B), our dynamic model shows that a dominant eusociality allele is more likely to invade under monandry (left, *S* = selection coefficient) than under polyandry (double or triple mating, middle and right), with the difference being more pronounced under high penetrance (*P* = 1, bottom vs. *P* = 0.1, top). In our model, monogamy and monandry are not strict requirements for the evolution of eusociality though, as even with a brood provisioning efficiency that is assumed identical for workers and nonsocial solitary mothers, eusociality could in principle invade if the per-capita productivity *b* is high enough. At the same time, it is possible that in practice, ancestrally, per-capita productivity was not high enough for these demographic benefits to be large enough to allow eusociality to invade under multiple mating. Indeed, for the empirically observed value of *b* in *Polistes gallicus* paper wasps (orange line), eusociality can only invade if helping behavior is expressed for the first fraction *q* < 0.7 of the entire season (+ = observed value of *q*). Results also show that eusociality is more likely to spread under high penetrance (*P* = 1, bottom) than under low penetrance (*P* = 0.1, top). With high penetrance, more workers are produced early on in the season (before switchpoint *t* = *q.L*), which strengthens selection for sociality and increases the likelihood that it can overpower genetic drift (the thin white line indicates the contour where the selection differential *S* = 0.01, and the dashed white line shows the minimum per-capita productivity *b* that would be required for at least 10% of all M-type colonies to produce one worker). Consistent with the expected maternal optimum, all plots assume an ancestral numerical investment in females throughout the season of *f_1_ *= *f_2_ *= 0.5; plots for the derived situation with *f_1_ *= 1 and *f_2_ *= 0.54, as observed in *P. gallicus*, are shown in Figure S7. For details, see Mathematica notebook in Dataset S3.

To explore the evolutionary implications, we embedded our ODE model of within-season dynamics into a recurrence equation system to model across-year gene frequency dynamics of a dominant eusociality allele (*A*) in a haplodiploid population (Figure 2A, see the Online Appendix). Our dynamic population genetic model, considering maternally (M-type) and paternally (P-type) inherited alleles, shows that with convex or near-linear returns, traditional relatedness predictions hold: A dominant eusociality allele spreads more easily under monandry (*m_e_*​ = 1, ensuring high relatedness) than under polyandry (*m_e_*​ = 2 or *m_e_*​ = 3; Figure 3; Figure S7). Importantly, the model revealed that under monandry, eusociality alleles can spread even when helpers are no more efficient at rearing siblings than solitary breeders are at rearing their own offspring, due to intrinsic demographic benefits of sociality.

### Monogamy helps but is not strictly necessary for the evolution of eusociality

While monandry significantly expands the parameter space under which eusociality is selected for in our model (Figure 3), strict lifetime monogamy is not an absolute theoretical prerequisite for its evolution. Eusociality could, in principle, also evolve under conditions of double or even triple mating if the per-capita growth rate *b* were sufficiently high to overcome the diluted relatedness, even if individual worker efficiency simply matched that of solitary mothers. However, the feasibility of eusociality evolving under polyandry becomes an empirical question hinging on ancestral ecological conditions and achievable productivity rates. For instance, using the empirically observed value of *b* in our *P. gallicus* system (Figure 3, orange line), our model predicts that eusociality would only have been able to invade under single mating, and only if helping behavior was expressed for a sufficient portion of the early season (i.e., *q* < 0.7, with “+” in Figure 3 marking the observed *q*-value). This suggests that, in practice, ancestral per-capita productivity in many lineages may not have been high enough to allow eusociality to invade under conditions of multiple mating.

### Eusociality evolves more easily with genes of large effect

A final key insight from our model is that eusociality is more likely to spread if it is encoded by an allele of large effect (i.e., under high penetrance, *P*), allowing selection to be sufficiently strong to overcome genetic drift, particularly in smaller populations (Figure 3). For instance, for an effective population size (${{N}_e}$) of around 100, as reported in some social Hymenoptera (Dyson et al., 2021; Zayed et al., 2001; Zayed, 2004), an invading allele would need a selection strength $S > 1/{{N}_e} = 0.01$. Our results show that such selection strengths are more readily achieved with high penetrance (Figure 3, solid white contour line). High penetrance is also important to ensure that a sufficient proportion (e.g., ≥10%) of maternally derived (M-type) colonies produce at least one worker, which is a prerequisite for selection to act effectively on the eusociality allele (Figure 3, dashed white line). This suggests that inclusive fitness models, often derived under assumptions of low penetrance and vanishingly weak selection, may not always accurately capture the conditions under which eusociality could actually spread.

## Discussion

Our integrated experimental and theoretical study directly challenges the long-held view of prevalent diminishing returns at the origin of eusociality (Michener, 1964). We demonstrate that even when per-capita brood-rearing efficiency scales linearly with worker number, as supported by broader meta-analyses (Field et al., 2020; Jeanne et al., 2022), the fitness returns on the *probability of early-season helping* can be convex and increasing (Figure 1). This crucial distinction arises from acknowledging the crucial importance of timing. Early helping, even without immediate per-capita efficiency gains over solitary individuals, initiates a positive feedback loop by accelerating the production of more workers, thereby amplifying end-of-season reproductive output. This compounding demographic benefit, stemming from additive foundress and worker contributions to colony growth (Bulmer, 1994; Hovestadt et al., 2018; Oster et al., 1978), has often been overlooked in static, correlational studies (Field et al., 2020; Jeanne et al., 2022) but has profound implications for resolving Michener’s paradox (Michener, 1964). The fact that our quantitative estimates come from just 12 successful colonies in a single population is a possible limitation, as it means our data may not encompass the full genetic and phenotypic variation present in this species. Nevertheless, our theoretical model readily confirms the empirically observed relationship, which means our conclusions are likely robust.

Much of the controversy over returns on helping largely stems from differences in what studies measure (Jeanne et al., 2022). Some focus on instantaneous effects, like brood production rates, while others consider long-term fitness outcomes or returns on absolute worker numbers (e.g., Fromhage et al., 2011; Grinsted et al., 2018; Kennedy et al., 2021; Michener, 1964). This can be misleading, as even solitary ancestors possess baseline productivity (Figure S6). In contrast, our study emphasizes returns on the underlying probability of offspring helping, which better captures evolutionary dynamics at the origin of eusociality. It is clear that in the context of the evolution of eusociality, long-term fitness consequences over the entire colony lifetime and returns on helping probability are what actually matter, and our study is the first to quantify these. This perspective suggests that re-evaluating prior studies could alter conclusions; e.g., in the sweat bee *H. scabiosae*, per-capita fitness returns appear constant or increasing when the foundress is counted as a helper and colony survival benefits are included (Brand et al., 2014, Dataset S1). Similarly, even if brood-rearing efficiency decreases in large nests of species like *P. canadensis*, increasing returns from early helping likely persist in smaller, younger colonies due to these compounding effects (Kennedy et al., 2021).

Parameterizing our dynamic population genetic model with this empirical data further demonstrates that such convex fitness returns reinforce traditional relatedness predictions: Eusociality evolves most readily under monandry. This contrasts with earlier predictions based on hypothetical concave return functions, which suggested that worker sterility or eusociality might evolve more easily under conditions of low relatedness (Olejarz et al., 2015). However, our findings challenge the notion of monogamy as a strict, absolute prerequisite (Boomsma, 2007, 2009, 2013). Our model indicates that eusociality could, in principle, also invade under multiple mating if per-capita growth rates *b* were high enough (Figure 3). Empirically, however, typical per-capita growth rates, like those observed in *P. gallicus*, likely preclude this scenario for many lineages, thereby explaining why ancestral monogamy appears to be a consistent feature in the evolutionary history of social Hymenoptera (Hughes et al., 2008). Indeed, subsequent coevolution of life-history parameters after invasion would further strengthen selection for sociality (Figure S7), suggesting that once established, eusociality may become an evolutionary endpoint that is difficult to reverse, akin to an “evolutionary ratchet” that stabilizes these complex social systems over evolutionary time. Crucially, under monandry, our model reveals that eusociality can spread even when helpers are no more intrinsically efficient than solitary breeders, solely due to the compounding demographic advantages conferred by sociality and the “reproductive head start” provided by the presence of the foundress and the accelerated production of a workforce (Queller, 1989). Moreover, while the magnitude of these demographic advantages has been debated due to potential double counting issues in some inclusive fitness analyses (Gadagkar, 1990; Nonacs, 1991), our population genetic approach effectively avoids these ambiguities.

A final key conclusion from our study is the importance of considering the strength of selection in the evolution of eusociality. Unlike many inclusive fitness models derived in the limit of vanishingly weak selection (Boomsma, 2007, 2009, 2013; Crozier et al., 1996; Frank, 1998; Gadagkar, 1990, 1991; Queller, 1989; Quiñones et al., 2017; Rees-Baylis et al., 2024), our dynamic population genetic model is valid for any strength of selection. We find that eusociality is more likely to achieve relevant strengths of selection (i.e., sufficient to overcome genetic drift in typically small ancestral populations) when the underlying allele has high penetrance, leading to a large effect on the phenotype (Birch, 2017; Kimura, 1985) (Figure 3). While high penetrance was historically dismissed due to concerns it would prevent the production of dispersing sexuals (Charlesworth, 1978; Grafen, 1985), our assumption of time-limited expression of helping behavior (i.e., helpers are produced only during the ergonomic phase) averts this problem. Given that worker behavior could arise from the co-option of existing gene regulatory networks for maternal care (Amdam et al., 2006; Linksvayer et al., 2005; Rehan et al., 2014; Toth et al., 2007), we believe that mutations of relatively large effect influencing the propensity to stay and help are plausible in the early stages of eusociality. Thus, contrary to weak-selection assumptions (Birch, 2017; Fisher, 1930; Frank, 1998; Hamilton, 1996; Quiñones et al., 2017), strong selection and large-effect mutations may merit greater attention in studies of major evolutionary transitions.

Methodologically, the dynamic complexity of colony growth and nonlinear fitness returns makes traditional actor-centered inclusive fitness analyses challenging (Allen et al., 2013; Gardner et al., 2011; Lehmann et al., 2020; Wenseleers et al., 2010). Indeed, recent dynamic colony growth models have utilized alternative modeling approaches like deterministic population genetic matrix models (Fromhage et al., 2011; Liao et al., 2015; Nowak et al., 2010) or stochastic branching processes (Field et al., 2020; Fu et al., 2015). Actor-centered inclusive fitness analyses of any of these models would be nontrivial, and would require making a detailed matrix model stratified by colony size and sibship type to model how costs and benefits of helping depend on colony lifecycle stage and the frequency of the helper genotypes in the local sibship. Furthermore, the fact that genetic relatedness would be distorted by selection (Bulmer, 1994; Gardner et al., 2011; Grafen, 1985) would necessitate the use of a dynamically sufficient framework (Allen et al., 2013; Birch, 2017). Although formal inclusive fitness analysis is challenging here, the relevant selection pressures can still be interpreted intuitively as reflecting a balance between positive among-group selection (colonies with more helpers produce more dispersing sexuals) and negative within-group selection (females that help forgo direct reproduction and leave fewer personal descendants than nonhelpers) (Figure 2; Marshall, 2011; Price, 1972; Wade, 1980; Wenseleers et al., 2010; Williams et al., 1957).

In conclusion, our study provides a robust, empirically grounded framework for understanding the genetic and demographic factors favoring eusociality. By integrating experimental data with a dynamic population genetic model valid under any strength of selection, we offer significant new insights into the evolution of nonreproductive castes and social living. This approach, informed by realistic data and emphasizing the increasing returns to scale of early helping, advances our understanding of the evolutionary pathways leading to eusociality and provides a resolution to Michener’s long-standing paradox (Jeanne et al., 2022; Michener, 1964).

## Supplementary Material

qraf033_Supplemental_Files

## Data Availability

All data generated or analyzed during this study are included in the published article and its supplementary information files. The complete datasets supporting the conclusions of this article are available in the Mendeley Data repository, DOI: 10.17632/7hyy7bb4dc.2.
