# Supplementary material for: Early-season helping yields increasing returns to scale at the onset of eusociality
Source: Evol Lett. 2025 Sep 22;9(6):675–85. doi: 10.1093/evlett/qraf033 (PMC12676465; doi:10.1093/evlett/qraf033)
Supplement: qraf033_Supplemental_Files [file qraf033_supplemental_files.zip › DiPietro_etal_Appendix_EvolutionLetters.docx]

**Appendix**

**Population genetic model details**

**Overview of the model.** Our dynamic population genetic model was developed to investigate the conditions under which a dominant eusociality allele (A) could invade a nonsocial, wild-type population in an annual haplodiploid species. The model is empirically parameterized using data from our *P. gallicus* experiments (Table 1) and is valid under any strength of selection. The eusociality allele A, present in a proportion $p_{Aa}$ of all females in a given sibship, is assumed to be conditionally expressed with penetrance P in heterozygous female carriers (Aa) if they eclose early in the season, specifically before a fraction *q* of the total season length *L* has elapsed. Expression of the allele causes these females to stay at their natal nest and perform helper behaviors instead of dispersing to reproduce independently. This helping behavior is assumed to arise from the co-option of pre-existing gene regulatory networks encoding maternal care, which are present in ancestral solitary Hymenoptera, and therefore restricted to females (Amdam et al., 2006; Linksvayer et al., 2005; Rehan et al., 2014; Ross et al., 2013; Toth et al., 2007). The model consists of an Ordinary Differential Equation (ODE) system for within-season colony demographic dynamics and a system of recurrence equations for across-year allele transmission, assuming an annual lifecycle with synchronous mating, contrary to other previous dynamic population genetic models that assumed perennial lifecycles (Fromhage et al., 2011; Liao et al., 2015; Nowak et al., 2010).

**Across-year gene frequency dynamic (Recurrence equation system).** Outputs from the ODE model inform recurrence equations for allele A frequency change. We focus on the initial invasion conditions of a rare, dominant eusociality allele A occurring with low frequency *p_f_* and *p_m_* in female and male gametes. This allele can be inherited either maternally (M-type sibship, frequency *p_m_* ≅ *2.p_f_*) or paternally (P-type sibship, frequency *p_f_*  ≅ *m_e_.p_m_*, where *m_e_* is the effective female mating frequency) (Figure 2).

In *M*-type sibships, the proportion of Aa offspring females is $p_{Aa M}=1/2$, while in *P*-type sibships it is $p_{Aa P}=$1/ *m_e_​*. We can denote the reproductive outputs at the end of the season for M and P type sibships, relative to those of a nonsocial wild type (where $p_{Aa W}=0$), as $G_{f M}$, ​$G_{m M}$, $G_{f P}$ and ​$G_{m P}$. The proportions of dispersing sexuals carrying the A allele can further be denoted as $\pi_{m M}=1/2$, $\pi_{m P}=0$ (assuming the absence of worker reproduction), $\pi_{f M}=\pi_{Aa}$ with $p_{Aa}=p_{Aa M}$ and $\pi_{f P}=\pi_{Aa}$ with $p_{Aa}=p_{Aa P}$.

The frequency vector of the A allele in female and male gametes in the next generation (*p_f_’* and *p_m_’*) is

$$\begin{aligned} \left( \begin{matrix} p_{f}' \\ p_{m}' \end{matrix} \right)=\mathbf{A}.\left( \begin{matrix} p_{f} \\ p_{m} \end{matrix} \right) \#(3) \end{aligned}$$

where the gene flow matrix **A** is

$$\begin{aligned} \mathbf{A}=\left( \begin{matrix} G_{f M}.\pi_{f M} & G_{m M} \\ \left( {m_{e}}/2 \right).G_{f P}.\pi_{f P} & 0 \end{matrix} \right) \boldsymbol{\#}(4) \end{aligned}$$

The selection differential for the eusociality allele A is $S=\lambda_{1}-1$, where $\lambda_{1}$ is the dominant eigenvalue of **A**:

$$\begin{aligned} \lambda_{1}=\frac{1}{2}\left( G_{f M}.\pi_{f M}+\sqrt{G_{f M}^{2}.\pi_{f M}^{2}+2.m_{e}.G_{m M}.G_{f P}.\pi_{f P}} \right) \#(5) \end{aligned}$$

In a large population, the eusociality allele can invade when the selection differential *S > 0*, which holds when

$$\begin{aligned} m_{e}.G_{f P}.\pi_{f P}>\frac{1-G_{f M}.\pi_{f M}}{G_{m M}.\left( \frac{1}{2} \right)} \#(6) \end{aligned}$$

Whether this condition is easier or harder to satisfy with higher mating frequencies $m_{e}$ depends on how relative group productivities *G* scale with the proportion of individuals carrying the helper genotype Aa (Olejarz et al., 2015). Using the group productivities as derived from our mechanistically motivated ODE system, however, shows that traditional relatedness predictions hold and that the critical per-capita growth rate *b* needed for eusociality to invade is higher under multiple mating, regardless of penetrance (Figure 3). This is as expected, given the increasing or near-linear returns on carrying the helper genotype (Olejarz et al., 2015; Figure 1 and Figure 2).

For smaller effective population sizes, the selection differential $S$ would have to be greater than ca. $1/N_{e}$ if selection is to overcome drift (Kimura, 1985). Although the effective population size of ancestral solitary breeding Hymenoptera is not really known, effective population sizes in social Hymenoptera as small as 100 have been reported (Dyson et al., 2021; Zayed, 2004). As an example, we therefore used numerical calculations to determine the critical *b* for the selection differential to exceed 1/100=0.01, as well as for the probability of at least 10% of *M*-type colonies to produce one worker (Figure 3, solid and dashed white contour lines).

In the general case, the minimum per-capita growth rate *b* required to allow eusociality to invade needs to be solved numerically from eqn. (6). For low penetrance, a first order Taylor approximation of *S* around *P* = 0 for the ancestral condition where *f_1_*=*f_2_*=0.5 yields

$$\begin{aligned} S=\frac{{Pⅇ}^{-L\mu}\left( 4ⅇ^{L\mu}\left( {1-ⅇ}^{Lq\nu} \right)m_{e}\mu\left( \mu-\nu\right)+b\left( 1+m_{e} \right)\left( ⅇ^{L\nu}\nu-ⅇ^{L\left( \nu+q\mu\right)}\nu-ⅇ^{L\mu}\mu+ⅇ^{L\left( q\nu+\mu\right)}\mu\right) \right)}{6m_{e}\left( ⅇ^{L\nu}-1 \right)\mu\left( \mu-\nu\right)} \#(7) \end{aligned}$$

In this case, the critical *b* for invasion in a large population is

$$\begin{aligned} b>\frac{4{m_{e}ⅇ}^{L\mu}\left( ⅇ^{Lq\nu}-1 \right)\mu\left( \mu-\nu\right)}{\left( 1+m_{e} \right)\left( {\nuⅇ}^{L\nu}-{\nuⅇ}^{L\left( \nu+q\mu\right)}-{\muⅇ}^{L\mu}+{\muⅇ}^{L\left( q\nu+\mu\right)} \right)} \#(8) \end{aligned}$$

Ignoring mortality ($\mu\longrightarrow0$ and $\nu\longrightarrow0$) this simplifies to

$$\begin{aligned} b.L.\frac{\left( 1+m_{e} \right)}{4m_{e}}=b.L.R_{sib}>\frac{1}{\left( 1-\frac{q}{2} \right)} \#(9) \end{aligned}$$

where $R_{sib}$ is the average life-for-life relatedness to siblings. Under weak selection and low penetrance, we then recover the traditional inclusive fitness result for matrifilial associations with a univoltine lifecycle (Crozier et al., 1996; page 62):

$$\begin{aligned} b.L.R_{sib}>1 \#(10) \end{aligned}$$

For *q* = 1/2 eusociality invades less easily:

$$\begin{aligned} b.L.R_{sib}>\frac{4}{3}\#(11) \end{aligned}$$

However, relevant selection strengths (*S* > 0.01) are more easily achieved for intermediate values of *q* under high penetrance (Figure 3, full white contour line).
