# Supplementary material for: Early-season helping yields increasing returns to scale at the onset of eusociality
Source: Evol Lett. 2025 Sep 22;9(6):675–85. doi: 10.1093/evlett/qraf033 (PMC12676465; doi:10.1093/evlett/qraf033)
Supplement: qraf033_Supplemental_Files [file qraf033_supplemental_files.zip › DiPietro_etal_SI_EvolutionLetters_REVISED.docx]

**Supplementary material**

**Early-season helping yields increasing returns to scale at the onset of eusociality**

Viviana Di Pietro^1*^, Ricardo Caliari Oliveira^1,2^, Tom Wenseleers^1*^

^1^Laboratory of Socioecology and Social Evolution, Department of Biology, KU Leuven, Leuven, Belgium

^2^Departament de Biologia Animal, de Biologia Vegetal i d’Ecologia. Universitat Autònoma de Barcelona. Bellaterra (Barcelona), Spain.

*Viviana Di Pietro, Tom Wenseleers

**Email:**  viviana.dipietro@kuleuven.be, tom.wenseleers@kuleuven.be

VDP: 0000-0003-1116-9795

RCO: 0000-0002-8996-1291

TW: 0000-0002-1434-861X

**Data Availability Statement**: All data generated or analysed during this study are included in this published article and its supplementary information files. The complete datasets supporting the conclusions of this article are available in the Mendeley Data repository, DOI: 10.17632/7hyy7bb4dc.1.

Figure S1. The timing of the switch to sexual production is independent of helper behaviour. (a) The mean first day of sexual emergence in *Polistes gallicus* did not differ significantly across our helper treatments, where ca. 50%, 25% or 0% of the newly eclosed workers were removed (Gamma GLM, Anova test: *F*₂,₉ = 0.079, *p* = 0.92, marginal means plus 95% confidence intervals shown). Across all treatments, sexuals emerged approximately 30 days [22.5-37.7] 95% C.I.s after the first worker eclosed. (b) The same conclusion was reached if treatment was continuously coded, with the timing of sexual emergence not being significantly related to the proportion of female offspring that were allowed to stay as helpers (Gamma GLM, β = -2.65, *t* = -0.14, *n* = 12, *p* = 0.89, marginal means plus 95% confidence intervals shown). In line with these observations, we assumed in our model that helper behaviour would be expressed for a fixed, constant fraction *q* of the total length of the season *L*. For details see section 1 in the R script in Dataset S2.

Figure S2. Helper behaviour does not affect nest survival probability. The proportion of *Polistes gallicus* nests that failed before having produced any dispersing sexuals did not differ significantly across our helper treatments, where ca. 50%, 25% or 0% of the newly eclosed workers were removed (binomial GLM, Anova test: LR *χ²* = 0.55, *n* = 12, *p* = 0.76, marginal means plus 95% confidence intervals shown). This suggests that whole-colony nest failure was independent of colony size, and there not being significant survivorship insurance benefits of helping, in line with earlier data from *P. dominula* (Shreeves et al., 2003). For details see section 2 in the R script in Dataset S2.

Figure S3. Per-capita brood rearing efficiency is independent of colony size. Across our *Polistes gallicus* helper treatments, the benefits of producing a higher or lower cumulative total number of workers on (a) total sexual, (b) virgin queen and (c) male production were all linear when measured over the entire colony lifecycle (relative likelihood of linear vs. quadratic least square model calculated from AICc values: 7.34, 10.23 and 5.95, the orthogonal quadratic terms in orthogonal quadratic polynomial models were all nonsignificant with *p* values of 0.47, 0.83 and 0.37, *n* = 12, model predictions plus 95% confidence intervals shown). In addition, the slopes of the regressions of total sexual, virgin queen and male production on the cumulative number of workers produced were not significantly different from 1 when analysed on a log-log scale, indicating equal reproductive efficiency in function of colony size (log-link Poisson GLMs of sexual production in function of the log of the cumulative number of workers produced, marginal trends tests performed using the *emmeans* R package, slopes (*SE*) and *p* values: 1.21 (0.12), *p*=0.07, 1.16 (0.15), *p*=0.29 and 1.27 (0.17), *p*=0.12). This is consistent with the equal reproductive efficiency in function of colony size found in most social insect groups^36^ and the linear productivity benefits reported in *Polistes* paper wasps when these were measured over the entire colony lifecycle (in *P. snelleni* and *P. chinensis* (Southon et al., 2015; Strassmann et al., 2003)). For details see section 3 in the R script in Dataset S2.


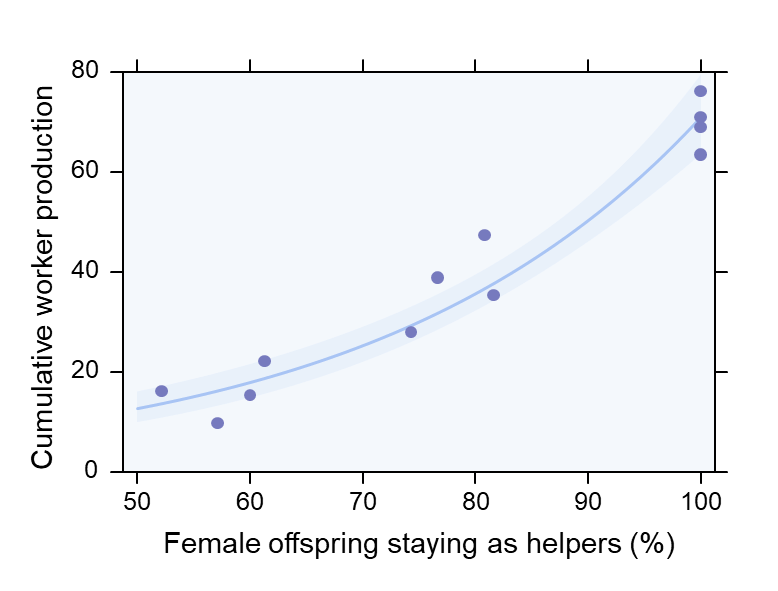


Figure S4. Compounding effects of early helping on cumulative worker production. In *Polistes gallicus*, workers produced early on help to produce more workers during the colony's ergonomic growth stage. This causes compounding effects of early helping on cumulative worker production and results in log-linear rather than a linear relationship between cumulative worker production and the proportion of offspring that stay as helpers during the colony growth stage (log-link Poisson GLM fit shown together with 95% confidence intervals). For details see section 4 in the R script in Dataset S2.

Figure S5. Model results replicate linear productivity benefits of cumulative worker number but increasing returns to scale of helping. (a) If one varies the probability for female to stay as helpers, our ODE model predicts an approximately linear relationship between sexual productivity and the cumulative number of workers that would be produced. This is in line with our *P. gallicus* results with experimentally manipulated proportions of helpers (Figure S3) and data from diverse groups of social insects (Jeanne et al., 2022). (b) Nevertheless, the cumulative worker production in function of the proportion of females that stay as helpers early on in the season is a convex function. This convexity arises because the benefits of helping behaviour cascade and compound over time, amplifying the overall colony productivity in a nonlinear way. (c) Consequently, the relationship between the proportion of females staying as helpers and total sexual productivity (or sexual productivity relative to that of a wild type solitary mother, Figure 2b) is also convex, reflecting increasing returns to scale of helping, in line with our empirical data (Figure 1). This is the relationship that actually matters in the context of the evolution of eusociality, as it is the probability of staying or leaving, and not worker number per se, that evolves. For details see Mathematica notebook in Dataset S3.

Figure S6. Decreasing returns to scale of helping can arise as an artefact. Some authors have inferred a decrease in reproductive efficiency in function of colony size and decreasing returns to scale of helping based on (a) a declining per capita sexual productivity (Michener, 1964) or (b) a slope of a regression line of sexual productivity versus worker number that on a log-log scale is less than one. Nevertheless, as mentioned by Jeanne et al. (2022), both methods can lead one to infer decreasing returns to scale of helping purely as an artefact, as a positive Y intercept results in an asymptote at zero workers in analyses of per capita sexual productivity (a) and a flat curve at low worker numbers in analyses of the slope of sexual productivity vs. worker number on a log-log scale (b). In the context of our model on the evolution of eusociality, these artefacts are clearly visible (a-b) due to the fact that an ancestral solitary breeding mother by definition has positive sexual productivity. For details see Mathematica notebook in Dataset S2.


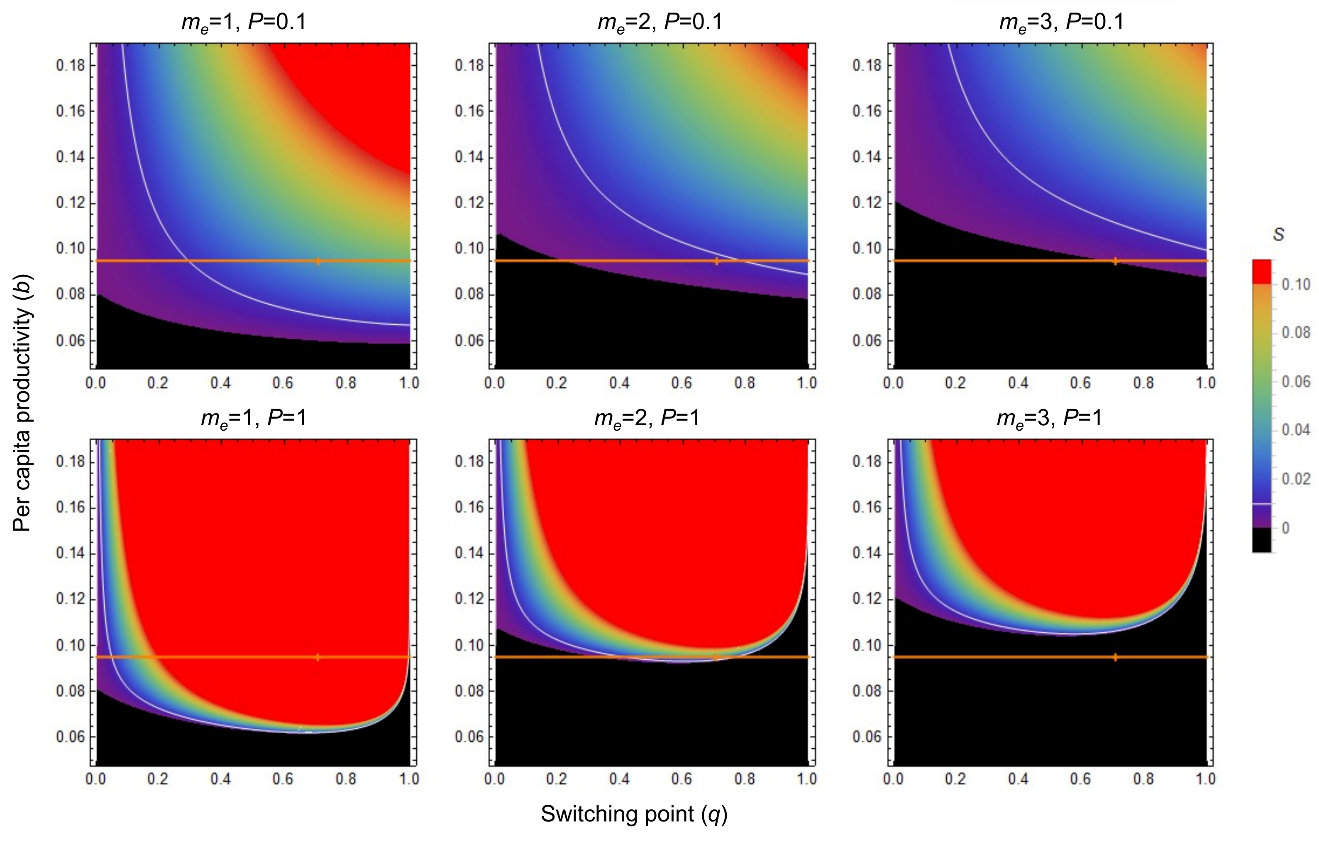


Figure S7. Selection for eusociality under a derived scenario. The selection differential for a dominant eusociality allele under single (left, *m_e_* = 1), double (middle, *m_e_* = 2) or treble (right, *m_e_* = 3) mating under high (bottom, *P* = 1) or low (top, *P* = 1) penetrance, now assuming that secondarily, following the invasion of eusociality in an ancestral nonsocial wildtype population where an equal sex ratio was produced throughout the season (*f_1_*=*f_2_*=0.5), the switching point *q* (proportion of the season before which workers are produced) and the numerical investment in females before and after this switchpoint *f_1_* and *f_2_* have coevolved and would be set as in our *Polistes gallicus* paper wasp model system, i.e. with exclusive production of females for the switchpoint (*f_1_*=1), and a slightly female biased numerical sex ratio thereafter (*f_2_* = 0.54), and with *q* = 0.71 and the rest of the parameters set as in Figure 1 (cf. Table 1). As expected, selection for eusociality then becomes much stronger and is selected for under a wider range of conditions, including under double mating. This implies that the evolution of eusociality is a one-way street, and that once eusociality spreads and the sex ratio and switching parameters further coevolve to their optimal value, it will be hard to reverse back to a nonsocial lifestyle, even under secondarily evolved multiple mating. For details see Mathematica notebook in Dataset S3.

**Legends for Dataset**

**Supplementary Data 1**: Zip file with R script and data on the fitness returns on helping in function of helper number in the primitively eusocial Halictine bee *Halicus scabiosae*, based on a reanalysis of the data of Brand & Chapuisat (2014).

**Supplementary Data 2**: Zip file with R script and sexual productivity and nest mortality data in function of the proportion of females that were allowed to help in our paper wasp model Polistes gallicus, reproducing Figure 1 and Figures S1 to S4.

**Supplementary Data 3**: Zip file with eusociality model in Mathematica notebook (.nb) and PDF format, reproducing Figures 2 and 3 and Figures S5 to S7.

**Supplementary Material References**

Jeanne, R. L., Loope, K. J., Bouwma, A. M., Nordheim, E. V., & Smith, M. L. (2022). Five decades of misunderstanding in the social Hymenoptera: a review and meta‐analysis of Michener's paradox. *Biol. Rev., 97*(4), 1559-1611.

Michener, C. D. (1964). Reproductive efficiency in relation to colony size in hymenopterous societies. *Insectes Soc., 11*, 317-341.

Shreeves, G., Cant, M. A., Bolton, A., & Field, J. (2003). Insurance–based advantages for subordinate co–foundresses in a temperate paper wasp. *Proc. Royal Soc. B, 270*(1524), 1617-1622.

Southon, R. J., Bell, E. F., Graystock, P., & Sumner, S. (2015). Long live the wasp: adult longevity in captive colonies of the eusocial paper wasp *Polistes canadensis* (L.). *PeerJ, 3*, e848.

Strassmann, J. E., Nguyen, J., Arévalo, E., Cervo, R., Zacchi, F., Turillazzi, S., & Queller, D. (2003). Worker interests and male production in *Polistes gallicus*, a Mediterranean social wasp. *J. Evol. Biol., 16*(2), 254-259.
