# Supplementary material for: Early-season helping yields increasing returns to scale at the onset of eusociality
Source: Evol Lett. 2025 Sep 22;9(6):675–85. doi: 10.1093/evlett/qraf033 (PMC12676465; doi:10.1093/evlett/qraf033)
Supplement: qraf033_Supplemental_Files [file qraf033_supplemental_files.zip › SUPPLEMENTARY MATERIAL.zip!/SUPPLEMENTARY MATERIAL/S1_reanalysis brand chapuisat 2014/brand_chapuisat_2014_colony productivity function.pdf]

Behav Ecol Sociobiol (2014) 68:291–298
DOI 10.1007/s00265-013-1644-0

ORIGINAL PAPER

Impact of helpers on colony productivity in a primitively eusocial bee

Nayuta Brand · Michel Chapuisat

Received: 23 August 2013 / Revised: 8 October 2013 / Accepted: 10 October 2013 / Published online: 30 October 2013
© Springer-Verlag Berlin Heidelberg 2013

Abstract Small societies of totipotent individuals are good systems in which to study the costs and benefits of group living that are central to the origin and maintenance of eusociality. For instance, in eusocial halictid bees, some females remain in their natal nest to help rear the next brood. Why do helpers stay in the nest? Do they really help, and if yes, is their contribution large enough to voluntarily forfeit direct reproduction? Here, we estimate the impact of helpers on colony survival and productivity in the sweat bee *Halictus scabiosae*. The number of helpers was positively associated with colony survival and productivity. Colonies from which we experimentally removed one helper produced significantly fewer offspring. However, the effect of helper removal was very small, on average. From the removal experiment, we estimated that one helper increased colony productivity by 0.72 additional offspring in colonies with one to three helpers, while the increase was smaller and not statistically significant in larger colonies. We conclude that helpers do actually help in this primitively eusocial bee, particularly in small colonies. However, the resulting increase in colony productivity is low, which suggests that helpers may be constrained in their role or may attempt to reproduce.

Keywords Social evolution · Cooperative breeding · Altruism · Eusociality · Halictid bees

Communicated by W. T. Wcislo

N. Brand · M. Chapuisat (✉)
Department of Ecology and Evolution, Biophore, UNIL-Sorge, University of Lausanne, 1015 Lausanne, Switzerland
e-mail: Michel.Chapuisat@unil.ch

N. Brand
e-mail: Nayuta.Brand@unil.ch

Introduction

How eusociality arises and is maintained is a major question in evolutionary biology, because eusocial groups contain individuals that do not reproduce and instead help others. The transition from solitary to social breeding and the emergence of a non-reproducing helper caste are best studied in small societies where all individuals are still able to mate and reproduce, such as those of many bees and wasps (e.g. Schwarz et al. 2007; Chapuisat 2010; Yagi and Hasegawa 2012). Such species show great lability in social organization and behaviour—subordinate individuals may increase their indirect fitness by helping relatives but may also obtain considerable direct fitness, for example when they inherit the nest (Hamilton 1964; Bourke 2011; Leadbeater et al. 2011; Schwarz et al. 2011).

In primitively eusocial insects, as in cooperatively breeding birds, young individuals may either leave the parental nest or stay in it in order to help, reproduce personally or wait for breeding opportunities. The pay-off of each option depends on multiple ecological and social factors that jointly influence the opportunities for independent breeding, the probability to inherit the nest, the efficacy of help in increasing the fitness of relatives, and the possibility to get a share of reproduction (e.g. Keller and Chapuisat 1999; Barclay and Reeve 2012; Hatchwell et al. 2013). Breeding cooperatively or helping also provides head start benefits, as well as an insurance-based advantage—if an individual dies, other group members will finish rearing the brood (Queller 1989; Gadagkar 1990; Field et al. 2000). Moreover, the mother or other members of the social group can influence the behavioural trajectories of potential helpers, for example by influencing their development or restricting their reproduction (Alexander 1974; Ratnieks and Wenseleers 2008; Brand and Chapuisat 2012).

The presence of multiple cooperating adults can greatly increase colony survival and productivity compared with lone

breeding females, particularly when the risk of predation, parasitism or usurpation is high or when ecological conditions are harsh (Hogendoorn and Zammit 2001; Dunn and Richards 2003; Rehan et al. 2011; Yagi and Hasegawa 2011; Yagi and Hasegawa 2012). Moreover, division of labour between breeders and helpers can further increase colony success, as specialists become more efficient (Bourke 1999; Thomas and Elgar 2003). Ultimately, the evolution of a specialized helper caste will depend on the indirect fitness benefits generated by helping, as compared with the direct fitness benefits obtained by breeding in the group or independently (worker efficiency, Crozier and Pamilo 1996). Measures of the costs and benefits of helping are therefore essential to gain insights into the evolution of a helper caste.

The average productivity *per helper* has often been estimated by collecting entire colonies in primitively social Hymenoptera (e.g. Michener 1964; Hogendoorn and Zammit 2001; Smith et al. 2007; Yagi and Hasegawa 2012). However, with this correlational approach, some third factor may jointly affect helper number and overall colony productivity. Moreover, each helper is weighted equally, while it seems likely that the first helpers will have a greater influence on colony survival and productivity. To measure the marginal contribution of each additional helper to colony survival and productivity, one could remove helpers experimentally. Removal experiments in wasps have demonstrated the benefits of foundress associations (Clouse 2001; Tibbetts and Reeve 2003; Sumner et al. 2010), and in particular, the importance of insurance-based advantage (Field et al. 2000; Lucas and Field 2011). To our knowledge, the impact of single helpers on the success of obligately but primitively eusocial colonies has not yet been investigated experimentally.

Here, we estimated the effect of helpers on colony survival and productivity in the primitively eusocial bee *Halictus scabiosae*. In this species, foundresses rear a first brood consisting principally of relatively small females that help their mother to rear a second brood of males and gynes (Brand and Chapuisat 2012). The first-brood females have the possibility to mate and reproduce, either in their natal nests or in other nests (Ulrich et al. 2009). Hence, it is interesting to examine whether helpers really increase colony survival and productivity, and by how much. The contribution of a helper to colony success may be sufficient to select for complete reproductive altruism (self-restrained due to kin selection when nestmate relatedness and worker efficiency are high; Crozier and Pamilo 1996; Wenseleers et al. 2004). Alternatively, the first-brood females may be constrained to a helper role, for example if their mothers restrict their pollen provision (Brand and Chapuisat 2012). We do not know if first-brood females can establish their own nests—due to their small size and late emergence, this seems unlikely. First-brood females may thus be doing the best of a bad job, either by helping relatives or by reproducing in their natal nest or in foreign nests (e.g. Richards et al. 1995; Lopez-Vaamonde et al. 2004; Ulrich et al. 2009; Leadbeater et al. 2011).

In a field experiment, we removed one helper (first-brood female) from half of the colonies and examined the effects of the number of helpers remaining in the colony and of the removal treatment on colony success. The quantitative estimate of the effect of helpers on productivity provides insights into the likelihood of voluntary reproductive altruism, versus enforced altruism or direct fitness gain. Our results thus contribute to the understanding of the principles governing the evolution and maintenance of eusociality.

Materials and methods

Study species and study site

Halictus scabiosae is a primitively eusocial halictid bee forming annual colonies. In May, overwintered females establish underground nests. They lay eggs in individual cells stocked with provisions of pollen and nectar (mass provisioning, Brand and Chapuisat 2012). The foundresses rear a first brood (B1) that emerges from the nests in June and July (Ulrich et al. 2009; Brand and Chapuisat 2012). The first brood consists primarily of small-sized females that generally stay in their natal colony to help raise a second brood (B2) of gynes and males (Batra 1966; Ulrich et al. 2009; Brand and Chapuisat 2012). Helpers excavate new cells, forage to provision the offspring and occasionally defend the nests, for instance against predators like ants, parasitic flies or intraspecific usurpers. The gynes and males from the second generation emerge from the nests in August and September. After mating, the gynes overwinter and become the next generation of foundresses in the following spring (Ulrich et al. 2009; Brand and Chapuisat 2012).

Our study site is a dry, south-exposed embankment located in Adlikon, near Zürich, in northern Switzerland (Brand and Chapuisat 2012). It contains a large population of *H. scabiosae*, with more than 1,000 nests per breeding season over an area of ca. 30×10 m. In spring 2009, we marked the nests with numbered nails and flags and selected 245 colonies that successfully produced one or more B1 helpers. Throughout the breeding season, we captured the bees by placing net traps on the colony entrance in the early morning (6–8 a.m.), before the bees became active (8.30–10 a.m.). We captured the foundresses between May 6th and June 4th and marked them with individual colour codes, using quick-drying honeybee-marking paints (Apicolori, Bienen-Meier Künten).

Helper removal experiment and measures of colony productivity

We randomly allocated 114 colonies to the control treatment (no helper removal) and 131 colonies to the removal treatment, which consisted in removing one B1 female helper before it started to help. Between June 25th and July 1st, when the first B1 females started to emerge, we trapped one B1 female per colony, on its first exit from the natal nest. At our study site, brood emergence was synchronized and started on this week for all studied colonies. We released this B1 female near her natal colony in the controls and removed her definitively in the removal treatment. We subsequently estimated the post-removal number of helpers by trapping, marking and releasing all other B1 females emerging from the colonies, on every clear day, until August 24th.

We estimated colony productivity by trapping, marking and releasing all individuals produced in the second brood (gynes and males). Individuals from the second brood emerged from the nests between August 11th and September 8th. Within each nest, the two broods are separated in time, and we could easily distinguish B2 from B1 females based on previous marking, head width (Brand and Chapuisat 2012), wing wear and mandibular wear (Mueller and Wolfmueller 1993).

To check that the trapping method provided accurate estimates of the number of B1 females and of colony productivity (number of B2 gynes and males), we excavated a sample of colonies that were not included in our control or removal colonies but were treated in the same way as control colonies with respect to bee capture and marking. We blew starch into the burrows to follow them more easily while digging, carefully excavated the entire nest and counted the number of offspring in brood cells. The average number of B1 females estimated by excavating colonies (2.54±0.61, $n=13$) was not significantly different from the one estimated by trapping (2.45±0.12, $n=245$; Wilcoxon text, $W=1627.5$, $P=0.89$). Similarly, the average colony productivity estimated by excavating colonies (7.69±1.69, $n=13$) was not significantly different from the one estimated by trapping in successful control colonies (4.81±0.61, $n=48$; Wilcoxon text, $W=220.5$, $P=0.11$).

Statistical analysis

We used generalized linear models (GLM) to test the effect of potential explanatory variables (number of B1 females, removal treatment and their interaction) on colony survival (characterized by the successful production of B2 offspring) and colony productivity (measured as the number of B2 offspring). In a first analysis, we used the 'post-removal number of helpers', in order to estimate the influence of the actual number of B1 females remaining in the control and removal colonies after the removal treatment. In a second analysis, we included the removal treatment, the 'pre-removal number of helpers' and their interaction, in order to examine the effect of removing one helper and assess if this effect varied with respect to the total number of B1 females produced by the colony.

We used a binomial GLM for analysing colony survival. For analysing colony productivity, we used a hurdle model with negative binomial and binomial error distributions, in order to appropriately handle the excess of zeros and overdispersion in the response variable (Zeileis et al. 2008). Adding quadratic and cubic polynomials to the model did not improve the fit. We assessed the significance of explanatory variables using stepwise log-likelihood ratio tests and sequentially removed non-significant terms. We performed *post hoc* pairwise comparisons using a simultaneous inference procedure (Hothorn et al. 2008).

We obtained qualitatively similar statistical results when we took the sex ratio and differential investment in each sex into account for measuring productivity (the dry weight of females is twice that of males in *H. scabiosae*, Brand and Chapuisat 2012) or when we restricted the analysis to the production of gynes only. All statistical analyses were carried out in R 2.10.1, using the packages multcomp, pscl, VGAM and lmtest (R Development Core Team 2011).

Results

Colony size, productivity and sex ratio

H. scabiosae formed very small colonies, which had low productivity. On average, the foundresses reared 2.58 ± 0.12 (mean±s.e.) offspring in their first brood, and the number of B1 females per colony ranged from 1 to 11, with a mean of 2.45±0.12 ($n=245$ colonies; Fig. 1). Colony survival from the first to second brood was moderate, as only 42 % of the 114 control colonies successfully raised a second brood. The 48 successful control colonies produced 4.81±0.61 gynes and males on average, and the mean productivity across all control colonies, including the ones that failed, was 2.03±0.34. The sex ratio was female biased in both broods, with 5 % ±1 % males in the first brood ($n=$ 245 colonies) and 15 %±4 % males in the second brood ($n=48$ control colonies). Across all colonies, the sex ratio in the second brood did not vary significantly with the pre-removal number of helpers ($\chi^2_1=0.22$, $P=0.64$), nor with the removal treatment ($\chi^2_1=1.46$, $P=0.23$).

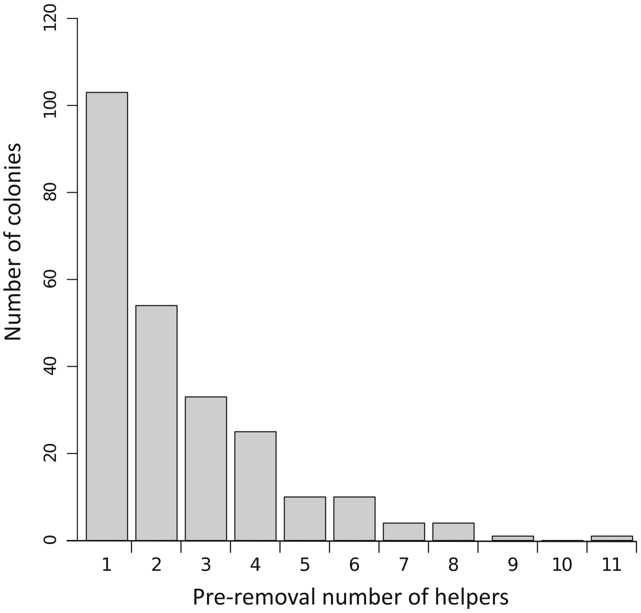

Fig. 1 Distribution of colonies according to the total number of helpers (B1 females) produced per colony

Influence of the post-removal number of helpers

The post-removal number of helpers (i.e. the number of B1 females remaining in the control and removal colonies after the removal treatment) showed a strong positive association with colony survival (Fig. 2a; stepwise log-likelihood ratio test: χ^2_1=48.27, p<0.001; n=245 colonies). Only 20 % of the colonies headed by foundresses with no helper left after removal successfully produced B2 offspring, and the survival rate gradually increased to more than 85 % for colonies with six or more helpers (Fig. 2a). In pairwise comparisons between colonies differing by one helper, colony survival increased significantly between one- and two-helper colonies (|z|=2.9, P=0.02), but not between zero- and one-helper colonies (|z|=2.24, P=0.13). According to the coefficients of the GLM, on average, each helper increased the odds ratio of successful against failed colonies by a factor of 1.8. The post-removal number of helpers was positively associated with the productivity of colonies that produced a second brood (Fig. 2b; χ^2_1=19.43, P<0.001; n=101 colonies). Based on the coefficients of the GLM, each helper in successful colonies increased colony productivity by a factor of 1.2 on average.

Across all colonies, including the ones that failed to produce any B2 offspring, the post-removal number of helpers was correlated with productivity (Fig. 2c; χ^2_1=65.93, P< 0.001). Colonies headed by foundresses with no helper left produced on average 0.28±0.10 B2 offspring, and the productivity rose to 7.00±1.95 in colonies with seven helpers (Fig. 2c). In pairwise comparisons, colony productivity increased significantly between zero- and one-helper colonies (|z|=2.68, P=0.05), as well as between one- and two-helper colonies (|z|=3.22, P=0.01).

Effect of helper removal

Colonies from which we removed one B1 helper (removal treatment) produced 1.59±0.24 males and gynes in the second brood, on average (n=131). Across all colonies, including the ones that failed to produce any B2 offspring, the removal treatment had a significant impact on colony productivity (χ^2_1=4.22, P=0.04), as did the pre-removal number of helpers (χ^2_2=67.31, P<0.001), and there was a significant interaction between the two variables, indicating that the effect of removing one helper depended on the total number of helpers produced by the colony (χ^2_1=5.7, P=0.02; Fig. 3). Indeed, the negative effect of helper removal on colony productivity decreased with the number of helpers (Fig. 3).

We estimated the *per helper* productivity by comparing the productivity of control colonies with the one of colonies in which we removed one helper. In small colonies, with one to three helpers before removal, control colonies produced 0.72 ±0.33 additional B2 offspring (mean±bootstrap s.e.), as compared with removal colonies, a difference that was significant (P=0.02, permutation test with 2,000 permutations). In large colonies, with four or more helpers, control colonies produced 0.41±1.19 additional offspring, as compared with removal colonies, a difference that was not significant due to large variance in productivity (P=0.37). Together, our removal experiment demonstrates that each individual helper contributes to increase colony productivity but that this increase is very small and variable, particularly in large colonies.

Discussion

Halictid bees are excellent model systems to study the evolutionary transitions between solitary and social life, because their social organization and reproductive strategies vary widely (Chapuisat 2010; Field et al. 2010; Yagi and Hasegawa 2012). Females from the first brood generally have multiple options, allowing researchers to investigate the costs and benefits of helping versus reproducing (e.g. Richards et al. 2005; Yagi and Hasegawa 2012). Here, we estimated the impact of helpers on colony survival and productivity in an obligately but primitively eusocial sweat bee, *H. scabiosae*.

Fig. 2 Influence of the number of B1 helpers remaining in control and removal colonies after the removal treatment on (**a**) colony survival (= proportion of nests that succeeded in producing B2 offspring±bootstrap s.e.), (**b**) colony productivity (= number of gynes and males±s.e.) of successful colonies, that is, colonies producing B2 offspring and (**c**) colony productivity across all colonies, including the ones that failed to produce any B2 offspring. The *solid line* fits predicted values from the model, for sample sizes of five or more. Sample sizes are indicated above the *x-axis* (n=number of colonies) ▶

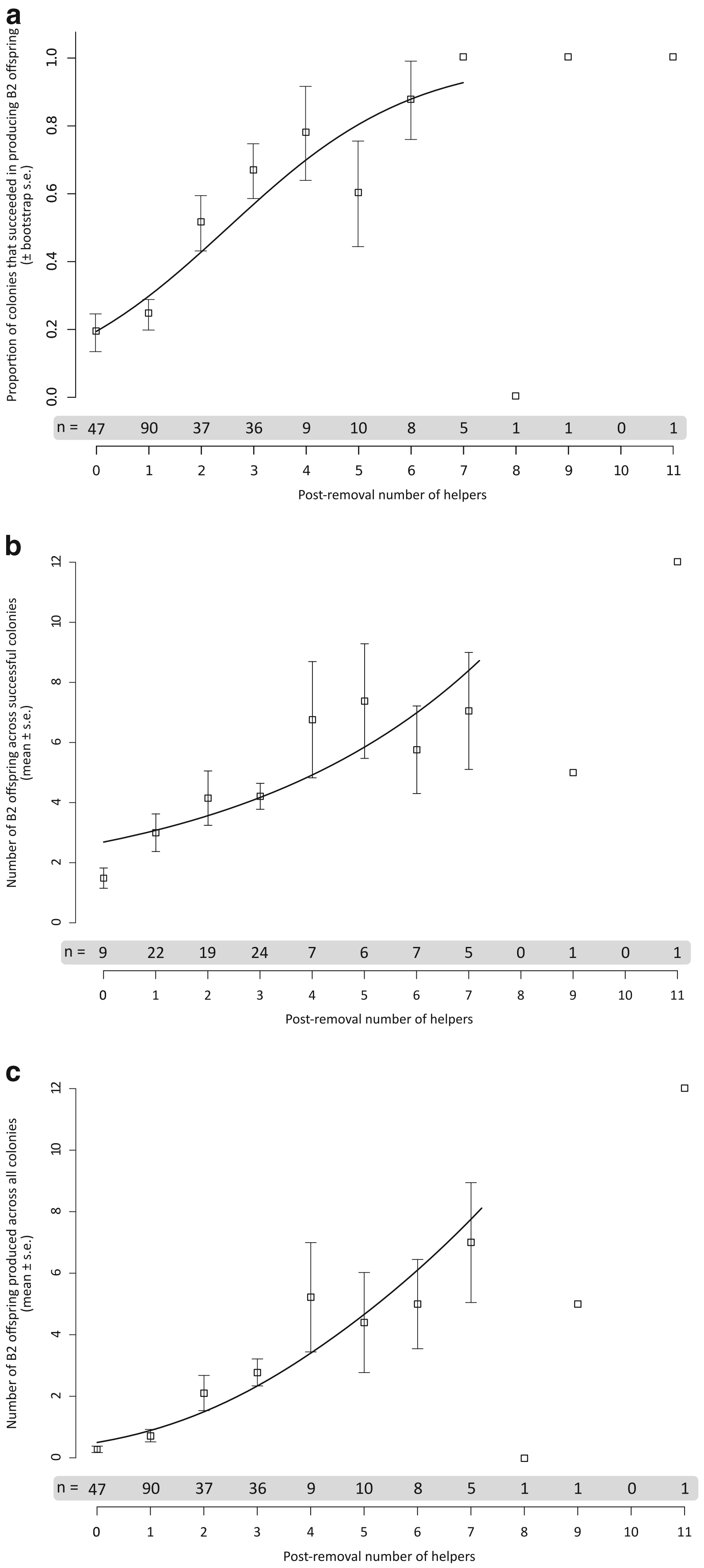
a
Proportion of colonies that succeeded in producing B2 offspring (± bootstrap s.e.)
0.0
0.2
0.4
0.6
0.8
1.0
n = 47 90 37 36 9 10 8 5 1 1 0 1
0 1 2 3 4 5 6 7 8 9 10 11
Post-removal number of helpers
b
Number of B2 offspring across successful colonies (mean ± s.e.)
0
2
4
6
8
10
12
n = 9 22 19 24 7 6 7 5 0 1 0 1
0 1 2 3 4 5 6 7 8 9 10 11
Post-removal number of helpers
c
Number of B2 offspring produced across all colonies (mean ± s.e.)
0
2
4
6
8
10
12
n = 47 90 37 36 9 10 8 5 1 1 0 1
0 1 2 3 4 5 6 7 8 9 10 11
Post-removal number of helpers

Colonies with more helpers had a higher probability of surviving from the first to second brood and produced more gynes and males. Overall, more than half of the colonies failed between the emergence of the first B1 helper and the production of the second brood, and the rate of colony failure dropped steadily as the number of helpers increased. The second helper appeared to have the strongest influence on colony success. High rates of colony failure are common in primitively eusocial species, in particular due to predation or usurpation, which are likely to be leading selective pressures for the evolution of sociality (Wenzel and Pickering 1991; Hogendoorn and Zammit 2001; Strohm and Bordon-Hauser 2003; Zammit et al. 2008).

In our removal experiment, we were interested in the actual contribution of one extra helper to colony survival and productivity, in relation to the number of helpers in the colony. We therefore removed one helper upon emergence in half of the colonies, before it provided any help. In contrast, other studies have looked at the effect of help already provided before helper removal. Colony members that die before the end of brood rearing have ‘assured fitness returns’ if other individuals finish rearing their brood (Gadagkar 1990). Assured fitness returns have been documented in wasps and allodapine bees that are progressive provisioners (Field et al. 2000; Hogendoorn and Zammit 2001; Lucas and Field 2011), as well as in some mass-provisioning halictid species (Kukuk et al. 1998; Smith et al. 2003).

The experimental removal of one helper upon emergence reduced colony productivity, but the effect depended on the number of helpers in the colony. The decrease in colony productivity was more pronounced and more consistent in small colonies than in large ones (Fig. 3). When examining colony survival and productivity in the function of the post-removal number of helpers, the second helper tended to have the largest influence, particularly for the survival of the colony, while the influence of the first helper was smaller (Fig. 2a and pairwise tests). Overall, the impact of helpers peaked at the second helper and became less pronounced in larger colonies. The small mean helper number in *H. scabiosae* (2.45±0.12) is in line with the finding that supplementary helpers had insignificant additional effect on colony productivity in colonies with more than three helpers.

A very small contribution of helpers to colony productivity seems common in primitively social halictid bees. So far, the *per capita* productivity had been estimated by regressing colony productivity over the total number of foundresses and helpers present in the colony, using entire colonies sampled at the end of the season. Typically, the *per helper* productivity ranged between 0.5 and 4 in social halictids, usually based on successful colonies only (Michener 1964; Boomsma and Eickwort 1993; Richards et al. 2005; Yagi and Hasegawa 2012). However, in these correlational data, it is hard to disentangle the influence of helpers from that of other factors linked to colony characteristics or foundress quality. Moreover, the influence of helpers on colony survival is not taken into account when only successful colonies are analysed.

In our experimental study, the average contribution of one helper to the final productivity of colonies was less than one additional offspring, when measured across all colonies, including the ones that failed. Why do foundresses bother to produce a helper if it contributes to less than one extra

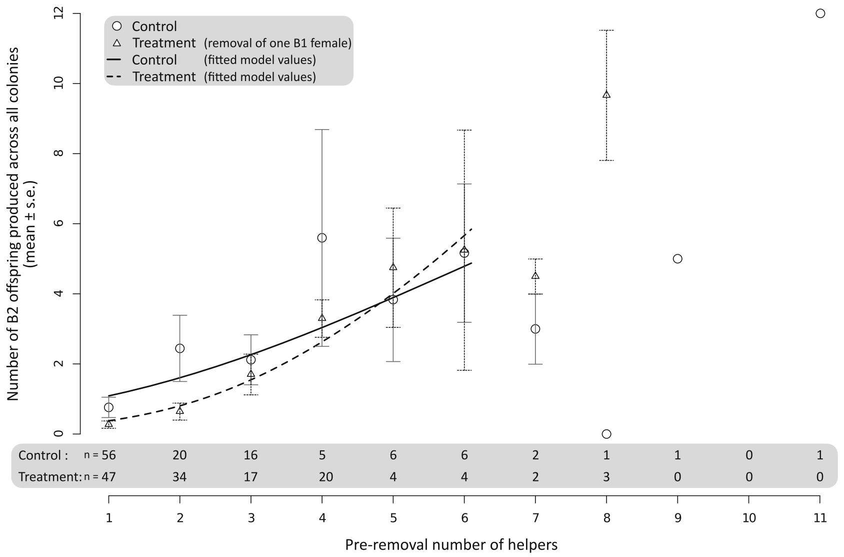

Fig. 3 Effect of helper removal on colony productivity across all colonies, including the ones that failed to produce any B2 offspring, in the function of the total number of B1 helpers produced by the colony (*x-axis*). The average (±s.e.) number of gynes and males produced are shown for control colonies (*circles*) and for colonies in which we had removed one B1 helper (*triangles*). The *solid line* and *dashed line* fit predicted values from the model for control colonies and helper removal colonies, respectively, for sample sizes of four or more. Sample sizes are indicated above the *x-axis* (*n*=number of colonies)

individual, on average? Part of the answer may lie in the fact that gynes are larger than helpers and require larger pollen and nectar provisions. Foundresses may restrict the pollen and nectar provisions of their first-brood daughters in order to force them to help rearing larger B2 offspring, rather than reproducing independently (the parental manipulation hypothesis; Alexander 1974; Kapheim et al. 2011; Brand and Chapuisat 2012). In *H. scabiosae*, the size difference between gynes and helpers amounts to 24 and 105 % for dry weight and absolute fat weight measured by lipid extraction, respectively (Brand and Chapuisat 2012). Hence, the pollen and nectar provisions provided to the second brood are 45 % heavier in terms of dry weight than those provided to the first brood (Brand and Chapuisat 2012). If we take this differential provisioning into account, the average *per capita* productivity of a helper becomes close to one in small colonies.

Overall, our data demonstrate that helpers in *H. scabiosae* do actually help and increase colony survival and productivity, particularly in small colonies. However, the average contribution per helper remained very small and became statistically undetectable in large colonies. It thus appears that helpers are doing the best of a bad job. They may have no chance for independent breeding, either because they lack the energetic reserves required for founding nests independently, due to restricted pollen provisions provided by their mothers (Brand and Chapuisat 2012), or because of adverse ecological conditions (Yagi and Hasegawa 2012). Yet putative helpers may attempt to reproduce in their natal nest or in other nests and thus gain direct fitness (Lopez-Vaamonde et al. 2004; Ulrich et al. 2009). More generally, a small impact of helpers on colony productivity is consistent with the frequent bidirectional transitions between solitary and eusocial organizations that have been documented in halictid bees (Eickwort et al. 1996; Hirata and Higashi 2008; Field et al. 2010; Gibbs et al. 2012).

Acknowledgments We thank Nelly di Marco, Joris Bessan, Sophie Berset, Michiel B. Djikstra, Thomas Martignier, Jérémie Projer, Aude Rogivue, Andrès Salazar Jaramillo, Luc Dolivo, Georg Hoffmann and Anabelle Reber for help in the field or the lab. We also thank Pierre Bize, Pierre Fontanillas, Anabelle Reber, Nicolas Salamin and Eric Lucas for advice on statistics; Michiel B. Djikstra for discussions on experimental set-up; and Jessica Purcell, Eric Lucas, William T. Wcislo and two anonymous reviewers for comments on the manuscript. This study was supported by grant 31003A_125306 from the Swiss National Science Foundation, as well as by grants from the Fondation Herbette and the Société Académique Vaudoise.

References

Alexander RD (1974) The evolution of social behavior. Annu Rev Ecol Syst 5:325–383

Barclay P, Reeve HK (2012) The varying relationship between helping and individual quality. Behav Ecol 23:693–698

Batra SWT (1966) Nesting behavior of *Halictus scabiosae* in Switzerland (Hymenoptera Halictidae). Ins Soc 13:87–92

Boomsma JJ, Eickwort GC (1993) Colony structure, provisioning and sex allocation in the sweat bee *Halictus ligatus* (Hymenoptera, Halictidae). Biol J Linn Soc 48:355–377

Bourke AFG (1999) Colony size, social complexity and reproductive conflict in social insects. J Evol Biol 12:245–257

Bourke AFG (2011) The validity and value of inclusive fitness theory. Proc R Soc B 278:3313–3320

Brand N, Chapuisat M (2012) Born to be bee, fed to be worker? The caste system of a primitively eusocial insect. Front Zool 9:35

Chapuisat M (2010) Evolution: plastic sociality in a sweat bee. Curr Biol 20:R977–R979

Clouse R (2001) Some effects of group size on the output of beginning nests of *Mischocyttarus mexicanus* (Hymenoptera: Vespidae). Fla Entomol 84:418–419

Crozier RH, Pamilo P (1996) Evolution of social insect colonies: sex allocation and kin selection. Oxford University Press, Oxford

Dunn T, Richards MH (2003) When to bee social: interactions among environmental constraints, incentives, guarding, and relatedness in a facultatively social carpenter bee. Behav Ecol 14:417–424

Eickwort GC, Eickwort JM, Gordon J, Eickwort MA (1996) Solitary behavior in a high-altitude population of the social sweat bee *Halictus rubicundus* (Hymenoptera: Halictidae). Behav Ecol Sociobiol 38:227–233

Field J, Shreeves G, Sumner S, Casiraghi M (2000) Insurance-based advantage to helpers in a tropical hover wasp. Nature 404:869–871

Field J, Paxton RJ, Soro A, Bridge C (2010) Cryptic plasticity underlies a major evolutionary transition. Curr Biol 20:2028–2031

Gadagkar R (1990) Evolution of eusociality: the advantage of assured fitness returns. Philos Trans R Soc B 329:17–25

Gibbs J, Brady SG, Kanda K, Danforth BN (2012) Phylogeny of halictine bees supports a shared origin of eusociality for *Halictus* and *Lasioglossum* (Apoidea: Anthophila: Halictidae). Mol Phylogenet Evol 65:926–939

Hamilton WD (1964) The genetical evolution of social behaviour. J Theor Biol 7:1–52

Hatchwell BJ, Sharp SP, Beckerman AP, Meade J (2013) Ecological and demographic correlates of helping behaviour in a cooperatively breeding bird. J Anim Ecol 82:486–494

Hirata M, Higashi S (2008) Degree-day accumulation controlling allopatric and sympatric variations in the sociality of sweat bees, *Lasioglossum* (*Evylaeus*) *baleicum* (Hymenoptera : Halictidae). Behav Ecol Sociobiol 62:1239–1247

Hogendoorn K, Zammit J (2001) Benefits of cooperative breeding through increased colony survival in an allodapine bee. Ins Soc 48:392–397

Hothorn T, Bretz F, Westfall P (2008) Simultaneous inference in general parametric models. Biom J 50:346–363

Kapheim KM, Bernal SP, Smith AR, Nonacs P, Wcislo WT (2011) Support for maternal manipulation of developmental nutrition in a facultatively eusocial bee, *Megalopta genalis* (Halictidae). Behav Ecol Sociobiol 65:1179–1190

Keller L, Chapuisat M (1999) Cooperation among selfish individuals in insect societies. Bioscience 49:899–909

Kukuk PF, Ward SA, Jozwiak A (1998) Mutualistic benefits generate an unequal distribution of risky activities among unrelated group members. Naturwissenschaften 85:445–449

Leadbeater E, Carruthers JM, Green JP, Rosser NS, Field J (2011) Nest inheritance is the missing source of direct fitness in a primitively eusocial insect. Science 333:874–876

Lopez-Vaamonde C, Koning JW, Brown RM, Jordan WC, Bourke AFG (2004) Social parasitism by male-producing reproductive workers in a eusocial insect. Nature 430:557–560

Lucas ER, Field J (2011) Assured fitness returns in a social wasp with no worker caste. Proc R Soc B 278:2991–2995

Michener CD (1964) Reproductive efficiency in relation to colony size in hymenopterous societies. Ins Soc 4:317–342

Mueller UG, Wolfmueller B (1993) A method for estimating the age of bees: age-dependent wing wear and coloration in the wool-carder bee *Anthidium manicatum* (Hymenoptera, Megachilidae). J Insect Behav 6:529–537

Queller DC (1989) The evolution of eusociality: reproductive head starts of workers. Proc Natl Acad Sci U S A 86:3224–3226

R Development Core Team (2011) R: a language and environment for statistical computing. In: R Foundation for Statistical Computing. http://www.R-project.org, Vienna, Austria

Ratnieks FLW, Wenseleers T (2008) Altruism in insect societies and beyond: voluntary or enforced? Trends Ecol Evol 23:45–52

Rehan SM, Schwarz MP, Richards MH (2011) Fitness consequences of ecological constraints and implications for the evolution of sociality in an incipiently social bee. Biol J Linn Soc 103:57–67

Richards MH, French D, Paxton RJ (2005) It's good to be queen: classically eusocial colony structure and low worker fitness in an obligately social sweat bee. Mol Ecol 14:4123–4133

Richards MH, Packer L, Seger J (1995) Unexpected patterns of parentage and relatedness in a primitively eusocial bee. Nature 373:239–241

Schwarz MP, Richards MH, Danforth BN (2007) Changing paradigms in insect social evolution: insights from halictine and allodapine bees. Annu Rev Entomol 52:127–150

Schwarz MP, Tierney SM, Rehan SM, Chenoweth LB, Cooper SJB (2011) The evolution of eusociality in allodapine bees: workers began by waiting. Biol Lett 7:277–280

Smith AR, Wcislo WT, O'Donnell S (2003) Assured fitness returns favor sociality in a mass-provisioning sweat bee, *Megalopta genalis* (Hymenoptera: Halictidae). Behav Ecol Sociobiol 54:14–21

Smith AR, Wcislo WT, O'Donnell S (2007) Survival and productivity benefits to social nesting in the sweat bee *Megalopta genalis* (Hymenoptera: Halictidae). Behav Ecol Sociobiol 61:1111–1120

Strohm E, Bordon-Hauser A (2003) Advantages and disadvantages of large colony size in a halictid bee: the queen's perspective. Behav Ecol 14:546–553

Sumner S, Kelstrup H, Fanelli D (2010) Reproductive constraints, direct fitness and indirect fitness benefits explain helping behaviour in the primitively eusocial wasp, *Polistes canadensis*. Proc R Soc B 277: 1721–1728

Thomas ML, Elgar MA (2003) Colony size affects division of labour in the ponerine ant *Rhytidoponera metallica*. Naturwissenschaften 90: 88–92

Tibbetts EA, Reeve HK (2003) Benefits of foundress associations in the paper wasp *Polistes dominulus*: increased productivity and survival, but no assurance of fitness returns. Behav Ecol 14:510–514

Ulrich Y, Perrin N, Chapuisat M (2009) Flexible social organization and high incidence of drifting in the sweat bee, *Halictus scabiosae*. Mol Ecol 18:1791–1800

Wenseleers T, Hart AG, Ratnieks FLW (2004) When resistance is useless: policing and the evolution of reproductive acquiescence in insect societies. Am Nat 164:E154–E167

Wenzel JB, Pickering J (1991) Cooperative foraging, productivity, and the central limit theorem. Proc Natl Acad Sci U S A 88: 36–38

Yagi N, Hasegawa E (2011) Both cooperation and nest position improve larval survival in the sweat bee, *Lasioglossum* (*Evylaeus*) *baleicum*. J Ethol 29:63–67

Yagi N, Hasegawa E (2012) A halictid bee with sympatric solitary and eusocial nests offers evidence for Hamilton's rule. Nat Commun 3:939

Zammit J, Hogendoorn K, Schwarz MP (2008) Strong constraints to independent nesting in a facultatively social bee: quantifying the effects of enemies-at-the-nest. Ins Soc 55:74–78

Zeileis A, Kleiber C, Jackman S (2008) Regression models for count data in R. J Stat Softw 27:25
