# Supplementary material for: Early-season helping yields increasing returns to scale at the onset of eusociality
Source: Evol Lett. 2025 Sep 22;9(6):675–85. doi: 10.1093/evlett/qraf033 (PMC12676465; doi:10.1093/evlett/qraf033)
Supplement: qraf033_Supplemental_Files [file qraf033_supplemental_files.zip › SUPPLEMENTARY MATERIAL.zip!/SUPPLEMENTARY MATERIAL/S3_mathematica model/eusociality model_FINAL_REVISED.pdf]

Early-season helping yields increasing returns to scale at the onset of eusociality

Viviana Di Pietro1*, Ricardo Caliari Oliverira2 & Tom Wenseleers1
1 Laboratory of Socioecology and Social Evolution, Department of Biology, KU Leuven, Naamsestraat 59, 3000 Leuven, Belgium
2 Departament de Biologia Animal, de Biologia Vegetal i d' Ecologia - Universitat Autònoma de Barcelona, 08193 Bellaterra (Barcelona), Spain
*Corresponding author

Model setup: We consider an annual male-haploid species where early in the season (before a fraction *q* of the total length of the season with length *L* passed) some females may stay & help instead of dispersing if they carry and express a eusociality allele. We assume the allele is dominant and expressed with penetrance *P* in the first part of the season. We then use an ordinary differential equation (ODE) model to analyse the expected within-year dynamics and determine what sexual output incipient social colonies would produce in function of the proportion of females that stay and help during the ergonomic growth phase of the colony. This is measured by the expected number of sexuals alive at the end of the season, which is when we assume mating takes place. Subsequently, we plug the solutions of this ODE model into a recurrence equation model to analyse the cross-year gene frequency dynamics and determine under what conditions the eusociality allele would be able to invade in a nonsocial wild type population. We allow the sex ratio produced before or after the switchpoint to differ, though when considering the invasion of a eusociality allele in an ancestral nonsocial wild type population, we will assume an equal 1:1 investment sex ratio throughout the whole season (f1=f2=0.5), consistent with the maternal sex ratio optimum of a solitary species with a univoltine lifecycle and equal mortality in both sexes. The model is parameterised using data obtained from the annual univoltine paper wasp *Polistes gallicus*, where the effect of a given proportion of females staying and helping on colony productivity was empirically measured and was shown to be consistent with the proposed model, i.e. with sexual productivity in function of the probability of helping having a concave shape, implying increasing returns to scale.

Parameters:
t=time in days since nest founding
L=length of the season in days after which no more new individuals are produced; in our *Polistes gallicus* study: L=97 days (n=12 colonies, adding one generation for the few workers that were already present when observations started, which is 39 days, Pardi 1951[1])
q=proportion of the season after which all females that are produced disperse & mate; in our *Polistes gallicus* study sexual production (males and dispersing females) started on average 30 days after the start of our observations, which if we could count from nest initiation translates into q = (30+39)/97 days = 0.71
p=proportion of female offspring of a given mating type that stay as a helper before time t = q.L (this is a function of the proportion of offspring carrying a eusociality allele & penetrance of the eusociality allele)
P=penetrance of the dominant eusociality allele; note that although we are considering the invasion of a dominant eusociality allele, taking P=0.5 could either be interpreted as modelling the invasion

of a dominant allele expressed with 50% probability or as an obligately expressed codominant allele
f1=proportion of individuals produced before time Ts that are female; in our *Polistes gallicus* study: f1 = 1, but ancestrally we assume f1=0.5, in line with the maternal optimum of a solitary breeding mother
f2=proportion of individuals produced after time Ts that are female; in our *Polistes gallicus* study: f2 = 0.54 ([0.49-0.60] 95% CIs from binomial GLM, our data, N=12 colonies) [NOTE: for simplificity we are assuming in our model they have the same energetic cost as males, even though in *P. gallicus* the fresh weights were slightly higher for female foundresses than males, 61.78 mg, SD 13.96 mg, n=176 vs. 45.61 mg, SD 8.19 mg, n=105; the fresh weights of workers were 45.12 mg, SD 9.31 mg, n=231, data from 12 colonies]; but ancestrally we assume f2=0.5, in line with the maternal optimum of a solitary breeding mother
b=per capita productivity (birth rate, nr of new individuals produced per individual per day); in our *Polistes gallicus* study: b = 0.095 [0.093-0.097] 95% CIs based on fitting colony productivity data in function of proportion of helpers that stayed based on the solution of the ODE below (nonlinear Poisson ML fit done using bbmle::mle2, cf. R script); this is also very close to the observed average per capita productivity if we calculate it as the mean ratio of nr of eggs present in nests / (nr workers present x 10 day egg to larva development time) reported in Strassmann et al. 2003 [2], N=19 nests & using a 10 day egg to larva development time reported in Pardi 1951 [1] - we then get a similar average value of b=0.11.
μ=mortality rate of workers (per day=1/life expectancy in days); for *Polistes gallicus*: μ = 1/19.2 (using avg. worker life expectancy reported for *P. gallicus* in Southon et al. 2015 [3] & Pardi 1948 [4])
v=mortality rate of sexuals (per day=1/life expectancy in days); for *Polistes gallicus :* mortality rate *v* of female sexuals = 1/(19.2*5.8) (using avg worker longevity for P. gallicus & avg Q/W longevity of 5.8 measured in *P. lanio, P. simillimus, P. exclamans, P. fuscatus* & *P. gallicus/dominula* in Southon et al. 2015 [3]); mortality of male sexuals before mating is assumed identical.
[NOTE: we are currently ignoring foundress mortality, as loss of the foundress could result in nest inheritance by a mated daughter or the onset of male production by mated workers, which would result in direct fitness benefits, which is an option we would like to exclude in the current model, which concerns the evolution of helping behaviour by obligately sterile workers. We are also varying relatedness only by varying mating frequency and, as in our *P. gallicus* study population, are assuming nests are founded by single foundresses.]
m_e=effective female mating frequency
T_m=nr. of males alive at the end of the season at which point mating is assumed to take place
T_f=nr. of female sexuals alive at the end of the season at which point mating is assumed to take place
G_m=relative number of males produced by a eusocial sibship compared to by a non-social wild type
G_f=relative number of female sexuals produced by a eusocial sibship compared to by a non-social wild type

1. Set global parameters & options

Empirical parameter values for *L, q, f1, f2, b*, μ and *v* as measured in the primitively eusocial paper wasp *Polistes gallicus* :

```
In[1]:= LEst = 97;
qEst = 0.71;
f1Est = 1; (* numerical investment in females before switching time q.L *)
f2Est = 0.54; (* numerical investment in females after switching time q.L *)
bEst = 0.095; (* fitted from observed sexual output vs proportion of helpers
 relationship and expected values based on our ODE model below *)
μEst = 1 / 19.2;
vEst = 1 / (19.2 * 5.8);
```

Assumed ancestral values for *q* (0.5) and *f* (0.5, i.e. equal sex ratio throughout the season) at the invasion of eusociality :

```
In[5]:= qAnc = 0.5; (* assumed ancestral example value,
but plots are made for a range of possible q values *)
fAnc = 0.5; (* assumed ancestral value at the origin of sociality,
ie equal investment sex ratio *)
```

Set some global plot options :

```
In[7]:= SetOptions[{Plot, ListPlot, ListLinePlot, ListLogLogPlot, ContourPlot},
  TicksStyle → Directive[Black],
  FrameStyle → Directive[Black],
  Frame → True,
  LabelStyle → Directive[Black],
  BaseStyle → {FontColor → Black},
  FrameLabel → None
 ];
```

2. Calculation of the invasion conditions of an eusociality allele in a nonsocial ancestor population

2.1. Model dynamics of within-year colony demographics using an ODE system

Before we set up our recurrence equations to model across-year gene frequency dynamics and determine the conditions under which a eusociality can invade, let us write down an ODE system describing within-year colony demographics, taking care to explicitly track the production of eusocial Aa and wild-type aa females in function of the proportion of early-season females that are Aa (pAa), which would express helping with probability equal to the penetrance parameter P. Our model assumes that all helpers, i.e. each of the workers plus the foundress, whose contribution we count as equivalent to that of a worker, have identical, additive effects on the colony growth rate, which is assumed to be independent of colony size. This assumption of a constant per-capita birth rate *b* is in line with Jeanne et al. (2022)[5], who documented an approximately constant reproductive efficiency in function of colony size across all major Hymenopteran social insect taxa in a large meta-analysis of data from 80 social insect species, as well as our own data from *Polistes gallicus* (section 3 in supplemental R script) and earlier data from *Polistes snelleni* and *P. chinensis* (Inagawa et al. 2001 [6], Suzuki 1981 [7]), which also demonstrate a linear relationship between end-of-season reproductive output and the cumulative number of workers produced. We further take into account worker mortality and sexual mortality (parameters μ and *v*), which are assumed to be constant and independent of colony size, in line with earlier data from primitively eusocial paper wasps (Shreeves et al. 2003 [8]). Finally, we denote f1 as the numerical proportionate investment in females in the first part of the season (before a fraction q of the total season passed) where helper

behaviour is expressed, which is set at 1 to model our *P. gallicus* data, but ancestrally is assumed to have lied at 0.5, in line with the maternal optimum in a solitary breeding population with a univoltine lifecycle. Given these assumptions, the ODE system for the first phase of the year with helper behaviour can be written as

In[8]:=
```
(*Phase 1 Differential Equations*)
socPhase1 = DSolve[{
    F'[t] == 0, (* foundress compartment,
    currently we are ignoring foundress mortality as colony
     turnover would result in direct fitness benefits to helpers,
    which we would like to exclude in a model on the evolution of
     true eusociality, based purely on indirect fitness benefits *)
    W'[t] == b * f1 * pAa * P * (W[t] + F[t]) - μ * W[t], (* workers *)
    Wcum'[t] == b * f1 * pAa * P * (W[t] + F[t]),
    (* cumulative nr of workers produced, so ignoring mortality here *)
    Qaa'[t] == b * f1 * (1 - pAa) * (W[t] + F[t]) - ν * Qaa[t],
    (* aa dispersing female sexuals *)
    QAa'[t] == b * f1 * pAa * (1 - P) * (W[t] + F[t]) - ν * QAa[t],
    (* Aa dispersing female sexuals *)
    M'[t] == b * (1 - f1) * (W[t] + F[t]) - ν * M[t], (* males *)
    {F[0] == 1, W[0] == 0, Wcum[0] == 0, QAa[0] == 0, Qaa[0] == 0, M[0] == 0}},
   {F[t], W[t], Wcum[t], QAa[t], Qaa[t], M[t]}, t];

(*Retrieving solutions at switching time t = Ts = L*q *)
W1End = W[t] /. socPhase1[[1]] /. {t → L * q};
Wcum1End = Wcum[t] /. socPhase1[[1]] /. {t → L * q};
QAa1End = QAa[t] /. socPhase1[[1]] /. {t → L * q};
Qaa1End = Qaa[t] /. socPhase1[[1]] /. {t → L * q};
Q1End = QAa1End + Qaa1End;
M1End = M[t] /. socPhase1[[1]] /. {t → L * q};
```

where F, W, Qaa, QAa and M are compartments for the foundress, the number of workers, the number of aa and Aa dispersing female sexuals and the number of males. The cumulative number of workers produced Wcum is also kept to be able to relate our results to earlier discussions on reproductive efficiency in social insects (Jeanne et al. 2022 [5]). Foundress mortality, and subsequent nest inheritance (Nonacs 2001 [9], Leadbeater et al. 2011 [10]) or worker male production (Strassmann et al. 2003 [2]), is ignored to be able to focus on the evolution of genuine worker altruism, without the possibility for any direct fitness benefits.

By the same logic, the ODE for the second phase of the year, where helper behaviour is no longer expressed, and all female sexuals produced disperse, can be written as

In[15]:=
```
(*Phase 2 Differential Equations*)
socPhase2 = DSolve[{
    F'[t] == 0, (* foundress compartment, ignoring foundress mortality *)
    W'[t] == -μ * W[t], (* workers *)
    Qaa'[t] == b * f2 * (1 - pAa) * (W[t] + F[t]) - ν * Qaa[t],
    (* aa dispersing female sexuals *)
    QAa'[t] == b * f2 * pAa * (W[t] + F[t]) - ν * QAa[t],
    (* Aa dispersing female sexuals *)
    M'[t] == b * (1 - f2) * (W[t] + F[t]) - ν * M[t], (* males *)
    {F[0] == 1, W[L * q] == W1End,
     QAa[L * q] == QAa1End, Qaa[L * q] == Qaa1End, M[L * q] == M1End}},
   {F[t], W[t], QAa[t], Qaa[t], M[t]}, t];
```

By solving these ODEs, we can then determine the expected colony productivities in terms of the production of Aa or aa female and male sexuals (TfAa, Tfaa and Tm), the proportion of dispersing females that would be Aa (πAa) and the production of female and male sexuals relative to those produced by a solitary wild type mother (where pAa=0) (Gf and Gm) :

```
(* Colony productivities in terms of nr of sexuals alive at end of season;
note: solitary mothers would have these productivities but with p=0 *)
Clear[Tf, πAa, Tm, Wtot];
TfAa[pAa_, P_, b_, f1_, f2_, q_, L_, μ_, ν_] =
  Simplify[(QAa[t] /. socPhase2[[1]] /. {t → L})];
Tfaa[pAa_, P_, b_, f1_, f2_, q_, L_, μ_, ν_] =
  Simplify[(Qaa[t] /. socPhase2[[1]] /. {t → L})];
Tf[pAa_, P_, b_, f1_, f2_, q_, L_, μ_, ν_] =
  Simplify[TfAa[pAa, P, b, f1, f2, q, L, μ, ν] + Tfaa[pAa, P, b, f1, f2, q, L, μ, ν]];
πAa[pAa_, P_, b_, f1_, f2_, q_, L_, μ_, ν_] =
  Simplify[TfAa[pAa, P, b, f1, f2, q, L, μ, ν] / Tf[pAa, P, b, f1, f2, q, L, μ, ν]];
(* prop of dispersing females that would be Aa *)
Tm[pAa_, P_, b_, f1_, f2_, q_, L_, μ_, ν_] =
  Simplify[M[t] /. socPhase2[[1]] /. {t → L}];
Tsex[pAa_, P_, b_, f1_, f2_, q_, L_, μ_, ν_] =
  Tf[pAa, P, b, f1, f2, q, L, μ, ν] + Tm[pAa, P, b, f1, f2, q, L, μ, ν];
(* Cumulative total nr of dispersing female and male sexuals produced *)
Wtot[pAa_, P_, b_, f1_, q_, L_, μ_] = Simplify[Wcum1End];
(* Cumulative total nr of workers produced *)
Gf[pAa_, pAaW_, P_, b_, f1_, f2_, q_, L_, μ_, ν_] =
  Tf[pAa, P, b, f1, f2, q, L, μ, ν] / Tf[pAaW, P, b, f1, f2, q, L, μ, ν];
(* production of female sexuals relative to the solitary wild type *)
Gm[pAa_, pAaW_, P_, b_, f1_, f2_, q_, L_, μ_, ν_] =
  Tm[pAa, P, b, f1, f2, q, L, μ, ν] / Tm[pAaW, P, b, f1, f2, q, L, μ, ν];
(* production of male sexuals relative to the solitary wild type *)
```

```
In[26]:= Tfaa[0.5, 1, bEst, fAnc, fAnc, qAnc, LEst, μEst, νEst]
Out[26]= 1.97779

In[27]:= TfAa[0.5, 1, bEst, fAnc, fAnc, qAnc, LEst, μEst, νEst]
Out[27]= 1.12941

In[28]:= πAa[0.5, 1, bEst, fAnc, fAnc, qAnc, LEst, μEst, νEst]
Out[28]= 0.363481

In[29]:= Tm[0.5, 1, bEst, fAnc, fAnc, qAnc, LEst, μEst, νEst]
Out[29]= 3.95557

In[30]:= Wtot[0.5, 1, bEst, fAnc, qAnc, LEst, μEst]
Out[30]= 1.59258

In[31]:= Tfaa[1, 1, bEst, fAnc, fAnc, qAnc, LEst, μEst, νEst]
Out[31]= 0.

In[32]:= TfAa[1, 1, bEst, fAnc, fAnc, qAnc, LEst, μEst, νEst]
Out[32]= 3.15821
```

In[33]:=
```
πAa[1, 1, bEst, fAnc, fAnc, qAnc, LEst, μEst, νEst]
```
Out[33]=
```
1.
```
In[34]:=
```
Tm[1, 1, bEst, fAnc, fAnc, qAnc, LEst, μEst, νEst]
```
Out[34]=
```
5.75057
```
In[35]:=
```
Wtot[1, 1, bEst, fAnc, qAnc, LEst, μEst]
```
Out[35]=
```
4.77121
```

Note that in our model, the assumption that the per capita birth rate *b* is constant regardless of colony size leads to an approximately linear relationship between total sexual productivity and the season-long cumulative number of workers produced, i.e. in equal reproductive efficiency in function of colony size. This is in line with Jeanne et al. (2022) [5], who documented an approximately constant reproductive efficiency in function of colony size across all major Hymenopteran social insect taxa in a large meta-analysis of data from 80 social insect species, as well as our own data from *Polistes gallicus* (section 3 in supplemental R script) and earlier data from *Polistes snelleni* and *P. chinensis* (Suzuki 1981 [7], Inagawa et al. 2001 [6]), which also support a linear relationship between end-of-season reproductive output and the cumulative number of workers produced. Earlier suggestions of decreasing reproductive efficiency in function of group size (Michener 1964 [11]) have for the most part been rejected and have rather been shown to be due to methodological flaws in the original analyses (Jeanne et al. 2022 [5]).

Equally though, it is the case that in our model, the cumulative number of workers produced in function of the proportion *p* of females that stay as helpers early on in the season is a convex function. This convexity arises because the benefits of helping behaviour cascade and compound over time, amplifying the overall colony productivity in a nonlinear way. Consequently, the relationship between the proportion *p* of females staying as helpers and total sexual productivity (or sexual productivity relative to that of a wild type solitary mother, Fig. 2b) is also convex, reflecting increasing returns to scale of helping (Fig. 1). This is the relationship that actually matters in the context of the evolution of eusociality, as it is the probability of staying or leaving, and not worker number per se, that evolves. By analogy to recessiveness at the individual phenotype level, this situation has sometimes been referred to as “behavioural recessiveness” (Bulmer 1994 [12]). This relationship is consistent with our empirically measured values of the reproductive output of *Polistes gallicus* paper wasp nests with manipulated fractions of early season females that help (Fig. 1 in main text, cf. section 4.2.3 in supplemental R script). The convex relationship is opposite to the concave relationship that would be needed to allow eusociality to invade more easily under low relatedness and polyandry (cf. Olejarz et al. 2015 [13]), the argument being that in that situation polyandry would at invasion result in relatively more highly productive family groups with at least some helpers. In general, the fact that family groups with more Aa offspring are more productive causes positive among-colony selection for eusociality (Fig. 2).

These effects are illustrated in the graphs below.

```
(* Create a grid of p values from 0 to 1 in steps of 0.01 =
 prop. of females that stay as helpers *)
pValues = Range[0, 1, 0.01];

(* Evaluate sexual productivity and cumulative nr. of
 workers produced for parameters as observed in P. gallicus *)
ProdTablePg = Table[{Tf[p, 1, bEst, f1Est, f2Est, qEst, LEst, μEst, νEst],
     (* total female sexual production = Tf *)
    Tm[p, 1, bEst, f1Est, f2Est, qEst, LEst, μEst, νEst],
```

```
    (* total male sexual production = Tm *)
    Tsex[p, 1, bEst, f1Est, f2Est, qEst, LEst, μEst, νEst],
    (* total sexual production *)
    Wtot[p, 1, bEst, f1Est, qEst, LEst, μEst]},
   (* cumulative nr. of workers produced *)
   {p, pValues}];

(* Evaluate sexual productivity and cumulative
 nr. of workers produced for likely ancestral parameter
 values at the invasion of eusociality (q=f=0.5) *)
ProdTableAnc = Table[{Tf[p, 1, bEst, fAnc, fAnc, qAnc, LEst, μEst, νEst],
    (* total female sexual production = Tf *)
    Tm[p, 1, bEst, fAnc, fAnc, qAnc, LEst, μEst, νEst],
    (* total male production = Tm *)
    Tsex[p, 1, bEst, fAnc, fAnc, qAnc, LEst, μEst, νEst],
    (* total sexual production *)
    Wtot[p, 1, bEst, fAnc, qAnc, LEst, μEst]},
   (* cumulative nr. of workers produced *)
   {p, pValues}];

(*Separate out results for plotting*)
GyneProdPg = ProdTablePg[[All, 1]];
(* Sexual gyne productivity = Tf = Tfaa+TfAa *)
MaleProdPg = ProdTablePg[[All, 2]]; (* Sexual male productivity = Tm *)
TotSexProdPg = ProdTablePg[[All, 3]]; (* Total sexual productivity = Tf+Tm *)
WtotPg = ProdTablePg[[All, 4]]; (* Cumulative nr. of workers produced *)
PerCapGyneProdPg = GyneProdPg / WtotPg; (* Per capita gyne productivity *)
PerCapMaleProdPg = MaleProdPg / WtotPg; (* Per capita male productivity *)
PerCapTotSexProdPg = TotSexProdPg / WtotPg;
(* Per capita total sexual productivity *)
dataGyneProdPg = Transpose[{WtotPg, GyneProdPg}];
dataMaleProdPg = Transpose[{WtotPg, MaleProdPg}];
dataGyneProdPg2 = Transpose[{100 * pValues, GyneProdPg}];
dataMaleProdPg2 = Transpose[{100 * pValues, MaleProdPg}];
dataTotSexProdPg = Transpose[{WtotPg, TotSexProdPg}];
dataTotSexProdPg2 = Transpose[{100 * pValues, TotSexProdPg}];
dataWorkerProdPg = Transpose[{100 * pValues, WtotPg}];
dataPerCapGyneProdPg = Transpose[{WtotPg, PerCapGyneProdPg}];
dataPerCapMaleProdPg = Transpose[{WtotPg, PerCapMaleProdPg}];
dataPerCapTotSexProdPg = Transpose[{WtotPg, PerCapTotSexProdPg}];

GyneProdAnc = ProdTableAnc[[All, 1]];
(* Sexual gyne productivity = Tf = Tfaa+TfAa *)
MaleProdAnc = ProdTableAnc[[All, 2]]; (* Sexual male productivity = Tm *)
TotSexProdAnc = ProdTableAnc[[All, 3]];
(* Total sexual productivity = Tf+Tm *)
WtotAnc = ProdTableAnc[[All, 4]]; (* Cumulative nr. of workers produced *)
PerCapGyneProdAnc = GyneProdAnc / WtotAnc; (* Per capita gyne productivity *)
PerCapMaleProdAnc = MaleProdAnc / WtotAnc; (* Per capita male productivity *)
PerCapTotSexProdAnc = TotSexProdAnc / WtotAnc;
(* Per capita total sexual productivity *)
dataGyneProdAnc = Transpose[{WtotAnc, GyneProdAnc}];
dataMaleProdAnc = Transpose[{WtotAnc, MaleProdAnc}];
```

```
dataGyneProdAnc2 = Transpose[{100 * pValues, GyneProdAnc}];
dataMaleProdAnc2 = Transpose[{100 * pValues, MaleProdAnc}];
dataTotSexProdAnc = Transpose[{WtotAnc, TotSexProdAnc}];
dataTotSexProdAnc2 = Transpose[{100 * pValues, TotSexProdAnc}];
dataWorkerProdAnc = Transpose[{100 * pValues, WtotAnc}];
dataPerCapGyneProdAnc = Transpose[{WtotAnc, PerCapGyneProdAnc}];
dataPerCapMaleProdAnc = Transpose[{WtotAnc, PerCapMaleProdAnc}];
dataPerCapTotSexProdAnc = Transpose[{WtotAnc, PerCapTotSexProdAnc}];
```

Power::infy : Infinite expression $\frac{1}{0.}$ encountered. >>

Power::infy : Infinite expression $\frac{1}{0.}$ encountered. >>

Power::infy : Infinite expression $\frac{1}{0.}$ encountered. >>

Power::infy : Infinite expression $\frac{1}{0.}$ encountered. >>

Power::infy : Infinite expression $\frac{1}{0.}$ encountered. >>

Power::infy : Infinite expression $\frac{1}{0.}$ encountered. >>

```
In[73]:= lp1 = ListLinePlot[{dataMaleProdPg, dataGyneProdPg, dataTotSexProdPg,
                  dataMaleProdAnc, dataGyneProdAnc, dataTotSexProdAnc},
    Joined → True,
    PlotStyle → {{ColorData["HTML"]["DodgerBlue"], Thick},
      {ColorData["HTML"]["DeepPink"], Thick}, {Gray, Thick},
              {ColorData["HTML"]["DodgerBlue"], Thick, Dashed},
      {ColorData["HTML"]["DeepPink"], Thick, Dashed}, {Gray, Thick, Dashed}},
    FrameLabel → {"Cumulative nr. of workers produced", "Sexual production"},
    Frame -> True,
    LabelStyle → {FontFamily → "Helvetica", FontSize → 12},
    Background → None, ImageSize → 400, PlotRange → {{0, 82}, {0, 70}},
    PlotLegends →
     Placed[{"derived, males", "derived, females", "derived, total",
                        "ancestral, males", "ancestral, females",
       "ancestral, total"}, {Left, Top}],
    Prolog → {RGBColor[235 / 255, 243 / 255, 251 / 255],
      Rectangle[Scaled[{0, 0}], Scaled[{1, 1}]]}];
  lp2 = ListLinePlot[{dataWorkerProdPg, dataWorkerProdAnc},
    PlotStyle → {{Gray, Thick}, {Gray, Thick, Dashed}},
    FrameLabel → {"Female offspring staying as helpers (%)",
      "Cumulative nr. of workers produced"}, Frame -> True,
    LabelStyle → {FontFamily → "Helvetica", FontSize → 12},
    Background → None, ImageSize → 400, PlotRange → {{0, 100}, {0, 82}},
    PlotLegends → Placed[{"derived", "ancestral"}, {Left, Top}],
    Prolog → {RGBColor[235 / 255, 243 / 255, 251 / 255],
      Rectangle[Scaled[{0, 0}], Scaled[{1, 1}]]}];
  lp3 = ListLinePlot[{dataMaleProdPg2, dataGyneProdPg2, dataTotSexProdPg2,
                  dataMaleProdAnc2, dataGyneProdAnc2, dataTotSexProdAnc2},
    PlotStyle → {{ColorData["HTML"]["DodgerBlue"], Thick},
      {ColorData["HTML"]["DeepPink"], Thick}, {Gray, Thick},
              {ColorData["HTML"]["DodgerBlue"], Thick, Dashed},
      {ColorData["HTML"]["DeepPink"], Thick, Dashed}, {Gray, Thick, Dashed}},
    FrameLabel → {"Female offspring staying as helpers (%)",
      "Sexual production"}, Frame -> True,
    LabelStyle → {FontFamily → "Helvetica", FontSize → 12},
    Background → None, ImageSize → 400, PlotRange → {{0, 100}, {0, 50}},
    PlotLegends →
     Placed[{"derived, males", "derived, females", "derived, total",
                        "ancestral, males", "ancestral, females",
       "ancestral, total"}, {Left, Top}],
    Prolog → {RGBColor[235 / 255, 243 / 255, 251 / 255],
      Rectangle[Scaled[{0, 0}], Scaled[{1, 1}]]}];
  lp1
  lp2
  lp3
```

Out[76]=

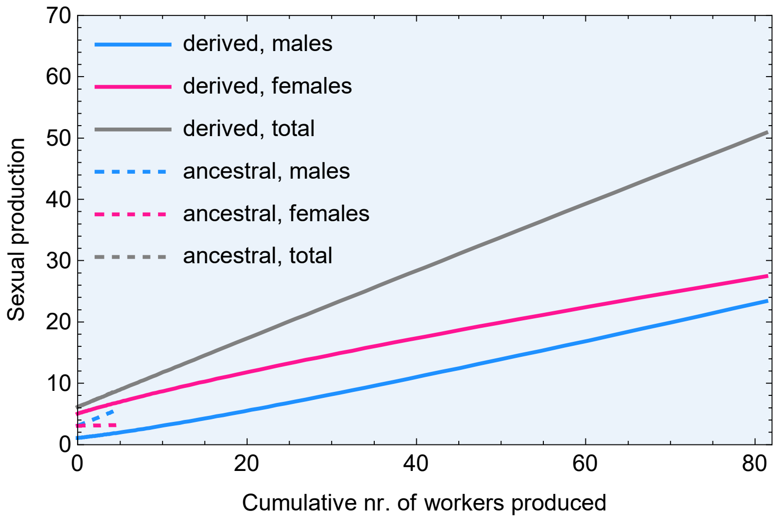

Out[77]=

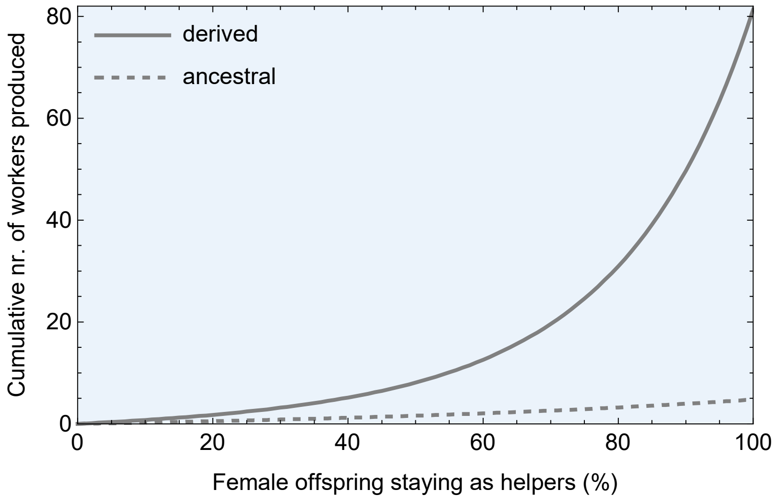

Out[78]=

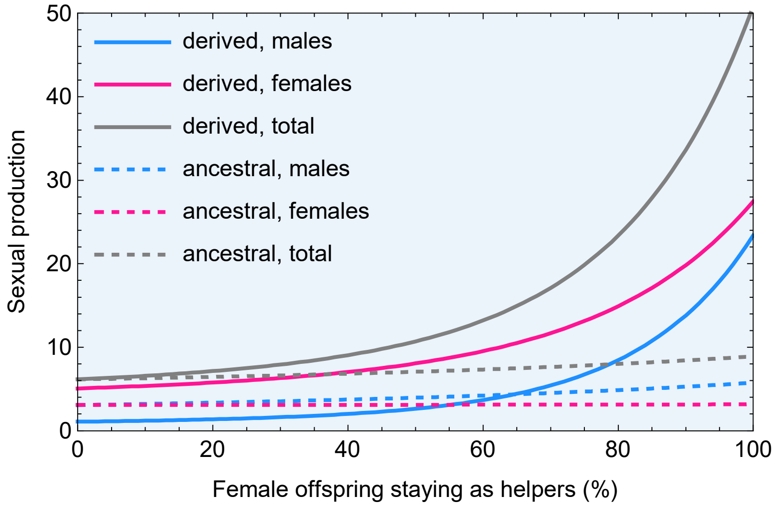

Also note that the relationship between per capita sexual productivity (sexual productivity divided by the cumulative number of workers produced) and the cumulative number of workers produced (colony size) is a decreasing function, but this is simply because of the fact that a sibship with zero workers still has positive sexual productivity because of the contribution of the mother, and is not evidence for diminishing returns to scale with increasing colony size per se, as is in fact mentioned by Michener 1964 [11], Cole 1984 [14] and Jeanne et al. 2022 [5].

```
In[79]:= lp1 = ListLinePlot[
    {dataPerCapMaleProdPg, dataPerCapGyneProdPg, dataPerCapTotSexProdPg,
                  dataPerCapMaleProdAnc,
     dataPerCapGyneProdAnc, dataPerCapTotSexProdAnc},
    PlotStyle → {{ColorData["HTML"]["DodgerBlue"], Thick},
      {ColorData["HTML"]["DeepPink"], Thick}, {Gray, Thick},
               {ColorData["HTML"]["DodgerBlue"], Thick, Dashed},
      {ColorData["HTML"]["DeepPink"], Thick, Dashed}, {Black, Thick, Dashed}},
    FrameLabel → {"Cumulative nr. of workers produced",
      "Per capita sexual productivity"}, Frame -> True,
    LabelStyle → {FontFamily → "Helvetica", FontSize → 12},
    Background → None, ImageSize → 400,
    PlotLegends →
     Placed[{"derived, males", "derived, females", "derived, total",
                        "ancestral, males", "ancestral, females",
       "ancestral, total"}, {Right, Top}],
    Prolog → {RGBColor[235 / 255, 243 / 255, 251 / 255],
      Rectangle[Scaled[{0, 0}], Scaled[{1, 1}]]}];
   lp2 = ListLogLogPlot[{dataMaleProdPg, dataGyneProdPg, dataTotSexProdPg,
                    dataMaleProdAnc, dataGyneProdAnc, dataTotSexProdAnc},
    Joined → True,
    PlotStyle → {{ColorData["HTML"]["DodgerBlue"], Thick},
      {ColorData["HTML"]["DeepPink"], Thick}, {Gray, Thick},
               {ColorData["HTML"]["DodgerBlue"], Thick, Dashed},
      {ColorData["HTML"]["DeepPink"], Thick, Dashed}, {Black, Thick, Dashed}},
    FrameLabel → {"Cumulative nr. of workers produced", "Sexual productivity"},
    Frame -> True,
    LabelStyle → {FontFamily → "Helvetica", FontSize → 12},
    Background → None, ImageSize → 400, PlotRange → {{0.07, 82}, {1, 150}},
    PlotLegends →
     Placed[{"derived, males", "derived, females", "derived, total",
                        "ancestral, males", "ancestral, females",
       "ancestral, total"}, {Left, Top}],
    Prolog → {RGBColor[235 / 255, 243 / 255, 251 / 255],
      Rectangle[Scaled[{0, 0}], Scaled[{1, 1}]]}];
   (* GraphicsGrid[{{lp1},{lp2}},ImageSize→400] *)
   lp1
   lp2
```

Out[81]=

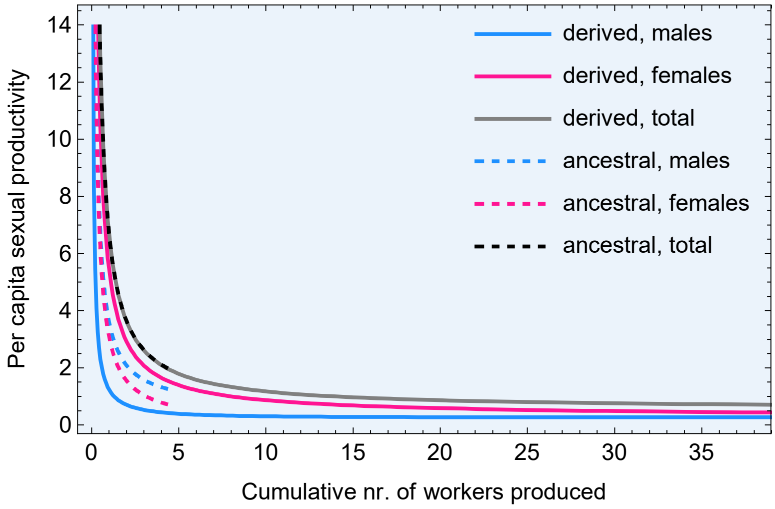

Out[82]=

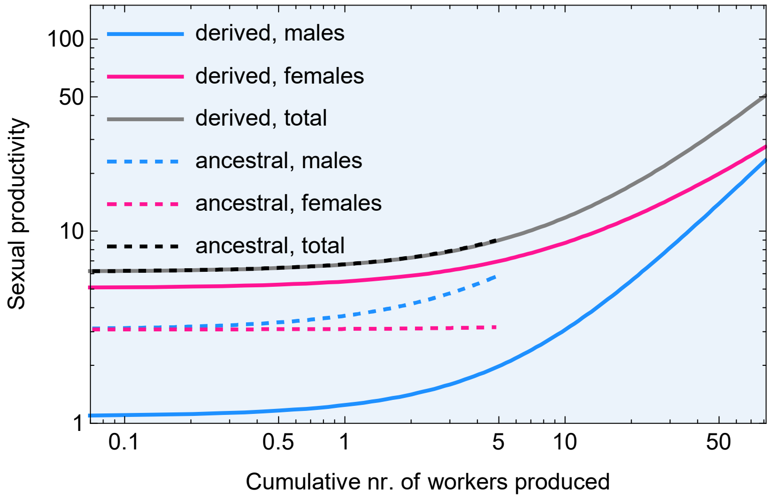

We can also plot the sexual productivity relative to that of the solitary breeding wild type G for female (Gf) or male sexuals (Gm), either for the likely ancestral situation (dashed line) or the for the derived parameters as observed in *P. gallicus*, here under the assumption of high penetrance (P=1). This shows significant colony-level benefits of helping, especially in terms of male production, for both sets of parameters, leading to positive among-group selection for eusociality, again with increasing (for derived parameters) or near-linear (for ancestral parameters) returns to scale of helping (cf. Fig. 2b, left panel) :

In[83]:=
```
plotcolonybenefit =
 Plot[{Gm[p / 100, 0, 1, bEst, f1Est, f2Est, qEst, LEst, μEst, νEst],
   Gf[p / 100, 0, 1, bEst, f1Est, f2Est, qEst, LEst, μEst, νEst],
   Gm[p / 100, 0, 1, bEst, fAnc, fAnc, qAnc, LEst, μEst, νEst],
   Gf[p / 100, 0, 1, bEst, fAnc, fAnc, qAnc, LEst, μEst, νEst]}, {p, 0, 100},
  Frame → True, PlotStyle → { {ColorData["HTML"]["DodgerBlue"]},
              {ColorData["HTML"]["DeepPink"]},
              {ColorData["HTML"]["DodgerBlue"], Dashed},
              {ColorData["HTML"]["DeepPink"], Dashed}},
  PlotRange → {0.5, 22.5},
  FrameLabel → {"Female offspring carrying eusociality allele (%)",
    "Productivity relative to solitary wild type"},
  PlotLegends → Placed[{"derived, males", "derived, females",
     "ancestral, males", "ancestral, females"}, {Left, Top}],
  LabelStyle → {FontFamily → "Helvetica", FontSize → 12},
  Background → None,
  Prolog → {RGBColor[235 / 255, 243 / 255, 251 / 255],
    Rectangle[Scaled[{0, 0}], Scaled[{1, 1}]]},
  ImageSize →
   400]
```

Out[83]=

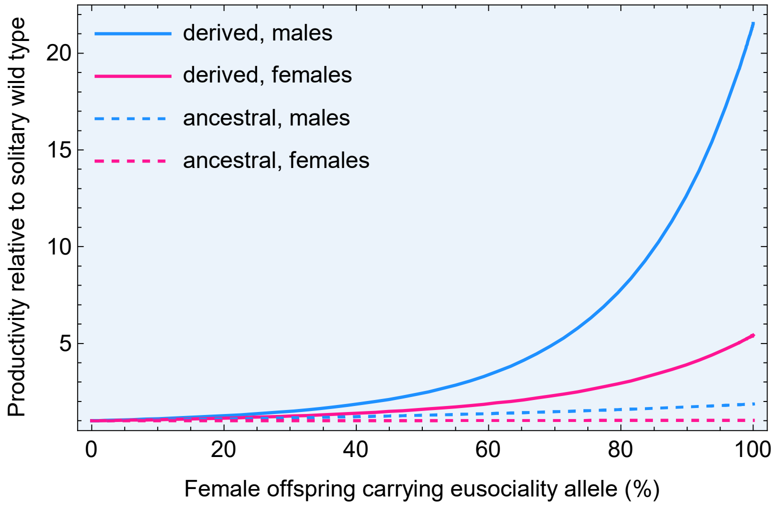

At the same time, there would be negative within-colony selection for the eusociality allele, since a significant portion of the Aa females would become sterile workers instead of dispersing sexuals a smaller proportion than pAa dispersing females will end up carrying the eusociality allele if Aa and aa segregate within the same colony (as early on in the season Aa females that express the helper phenotype with probability P will not contribute to the pool of dispersing sexuals). We can plot this in terms of the fold reduction in the probability that the allele would be carried by dispersing female sexuals within a given colony, here for either the ancestral (dashed line) or derived *P. gallicus* parameter values (full line) (cf. Fig. 2b, right panel) :
(NOTE: an equivalent expression would be
(1-*π*Aa[p/100,1,bEst,f1Est,f2Est,qEst,LEst,μEst,*ν*Est])/*π*Aa[p/100,1,bEst,f1Est,f2Est,qEst,LEst,μEst,*ν*Est] *)

In[84]:=
```
plotindividualcost =
 Plot[{Tfaa[p / 100, 1, bEst, f1Est, f2Est, qEst, LEst, μEst, νEst] /
    TfAa[p / 100, 1, bEst, f1Est, f2Est, qEst, LEst, μEst, νEst],
   Tfaa[p / 100, 1, bEst, fAnc, fAnc, qAnc, LEst, μEst, νEst] /
    TfAa[p / 100, 1, bEst, fAnc, fAnc, qAnc, LEst, μEst, νEst]}, {p, 0, 100},
  Frame → True, PlotStyle → { {ColorData["HTML"]["DeepPink"]},
             {ColorData["HTML"]["DeepPink"], Dashed}},
  FrameLabel → {"Female offspring carrying eusociality allele (%)",
    "Disadvantage of Aa over aa"},
  PlotLegends → Placed[{"derived, females", "ancestral, females"},
    {Right, Top}],
  LabelStyle → {FontFamily → "Helvetica", FontSize → 12},
  PlotRange → {-1.25, 50 + 1.25},
  Background → None,
  Prolog → {RGBColor[235 / 255, 243 / 255, 251 / 255],
    Rectangle[Scaled[{0, 0}], Scaled[{1, 1}]]},
  ImageSize →
   400]
```

Out[84]=

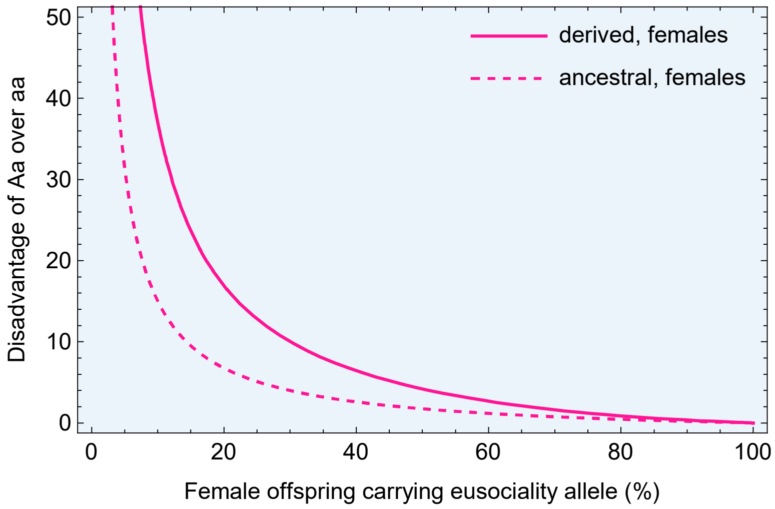

The relative group productivities of female and male sexuals solve to

In[85]:=
```
FullSimplify[Gf[pAa, pAaW, P, b, f1, f2, q, L, μ, ν]]
(* production of female sexuals relative to the solitary wild type *)
FullSimplify[Gm[pAa, pAaW, P, b, f1, f2, q, L, μ, ν]]
(* production of male sexuals relative to the solitary wild type *)
```

Out[85]=

$$\begin{aligned}
-\Big(\Big(&(b\, f1\, P\, pAaW + \nu - \mu)\,(-b\, f1\, P\, pAaW + \mu)\\
&\Big(e^{L\mu}\,(-f1 + e^{L\nu}\, f2 + f1\, P\, pAa - e^{Lq\nu}\,(f2 + f1\,(-1 + P\, pAa)))\,(\nu - \mu)^2\,\mu +\\
&b^2\, f1^2\, f2\, P^2\, pAa^2\,\Big(-e^{L\,(b\, f1\, P\, pAa\, q+\nu)}\,\nu + e^{L\,(q\,(b\, f1\, P\, pAa+\nu-\mu)+\mu)}\,\nu + e^{L\,(\nu+q\mu)}\,\nu - e^{L\,(q\nu+\mu)}\,\mu +\\
&e^{L\,(\nu+\mu)}\,(-\nu + \mu)\Big) - b\, f1\, P\, pAa\,(\nu - \mu)\,\Big(e^{L\,(b\, f1\, P\, pAa\, q+\nu)}\, f2\,\nu - e^{L\,(\nu+q\mu)}\, f2\,\nu -\\
&e^{L\,(q\,(b\, f1\, P\, pAa+\nu-\mu)+\mu)}\,(f2 + f1\,(-1 + P\, pAa))\,\nu + e^{L\,(\nu+\mu)}\, f2\,(\nu - 2\mu) +\\
&e^{L\,(q\nu+\mu)}\,(2\, f2 + f1\,(-1 + P\, pAa))\,\mu - e^{L\mu}\, f1\,(-1 + P\, pAa)\,(-\nu + \mu)\Big)\Big)\Big)\Big/\\
\Big(&(b\, f1\, P\, pAa + \nu - \mu)\,(-b\, f1\, P\, pAa + \mu)\,\Big(e^{L\mu}\,(f1 - e^{L\nu}\, f2 - f1\, P\, pAaW +\\
&e^{Lq\nu}\,(f2 + f1\,(-1 + P\, pAaW)))\,(\nu - \mu)^2\,\mu + b^2\, f1^2\, f2\, P^2\, pAaW^2\\
&\Big(e^{L\,(b\, f1\, P\, pAaW\, q+\nu)}\,\nu - e^{L\,(q\,(b\, f1\, P\, pAaW+\nu-\mu)+\mu)}\,\nu - e^{L\,(\nu+q\mu)}\,\nu + e^{L\,(\nu+\mu)}\,(\nu - \mu) + e^{L\,(q\nu+\mu)}\,\mu\Big) +\\
&b\, f1\, P\, pAaW\,(\nu - \mu)\,\Big(e^{L\,(b\, f1\, P\, pAaW\, q+\nu)}\, f2\,\nu - e^{L\,(\nu+q\mu)}\, f2\,\nu -\\
&e^{L\,(q\,(b\, f1\, P\, pAaW+\nu-\mu)+\mu)}\,(-f1 + f2 + f1\, P\, pAaW)\,\nu + e^{L\,(\nu+\mu)}\, f2\,(\nu - 2\mu) +\\
&e^{L\,(q\nu+\mu)}\,(-f1 + 2\, f2 + f1\, P\, pAaW)\,\mu - e^{L\mu}\, f1\,(-1 + P\, pAaW)\,(-\nu + \mu)\Big)\Big)\Big)
\end{aligned}$$

Out[86]=

$$\begin{aligned}
\Big(&(b\, f1\, P\, pAaW + \nu - \mu)\,(-b\, f1\, P\, pAaW + \mu)\\
&\Big(-e^{L\mu}\,(1 - f1 + e^{Lq\nu}\,(f1 - f2) + e^{L\nu}\,(-1 + f2))\,(\nu - \mu)^2\,\mu +\\
&b^2\, f1^2\,(-1 + f2)\, P^2\, pAa^2\,\Big(e^{L\,(b\, f1\, P\, pAa\, q+\nu)}\,\nu - e^{L\,(q\,(b\, f1\, P\, pAa+\nu-\mu)+\mu)}\,\nu -\\
&e^{L\,(\nu+q\mu)}\,\nu + e^{L\,(\nu+\mu)}\,(\nu - \mu) + e^{L\,(q\nu+\mu)}\,\mu\Big) + b\, f1\, P\, pAa\,(\nu - \mu)\\
&\Big(e^{L\,(q\,(b\, f1\, P\, pAa+\nu-\mu)+\mu)}\,(f1 - f2)\,\nu + e^{L\,(b\, f1\, P\, pAa\, q+\nu)}\,(-1 + f2)\,\nu - e^{L\,(\nu+q\mu)}\,(-1 + f2)\,\nu +\\
&e^{L\,(\nu+\mu)}\,(-1 + f2)\,(\nu - 2\mu) - e^{L\,(q\nu+\mu)}\,(1 + f1 - 2\, f2)\,\mu + e^{L\mu}\,(-1 + f1)\,(-\nu + \mu)\Big)\Big)\Big)\Big/\\
\Big(&(b\, f1\, P\, pAa + \nu - \mu)\,(-b\, f1\, P\, pAa + \mu)\,\Big(-e^{L\mu}\,(1 - f1 + e^{Lq\nu}\,(f1 - f2) + e^{L\nu}\,(-1 + f2))\\
&(\nu - \mu)^2\,\mu + b^2\, f1^2\,(-1 + f2)\, P^2\, pAaW^2\,\Big(e^{L\,(b\, f1\, P\, pAaW\, q+\nu)}\,\nu - e^{L\,(q\,(b\, f1\, P\, pAaW+\nu-\mu)+\mu)}\,\nu -\\
&e^{L\,(\nu+q\mu)}\,\nu + e^{L\,(\nu+\mu)}\,(\nu - \mu) + e^{L\,(q\nu+\mu)}\,\mu\Big) + b\, f1\, P\, pAaW\,(\nu - \mu)\\
&\Big(e^{L\,(q\,(b\, f1\, P\, pAaW+\nu-\mu)+\mu)}\,(f1 - f2)\,\nu + e^{L\,(b\, f1\, P\, pAaW\, q+\nu)}\,(-1 + f2)\,\nu - e^{L\,(\nu+q\mu)}\,(-1 + f2)\,\nu +\\
&e^{L\,(\nu+\mu)}\,(-1 + f2)\,(\nu - 2\mu) - e^{L\,(q\nu+\mu)}\,(1 + f1 - 2\, f2)\,\mu + e^{L\mu}\,(-1 + f1)\,(-\nu + \mu)\Big)\Big)\Big)
\end{aligned}$$

The equation for the proportion of female sexuals that will be Aa solves to

In[87]:=
```
FullSimplify[πAa[pAa, P, b, f1, f2, q, L, μ, ν]]
```

Out[87]=

$$\begin{aligned}
-\Big(\Big(&pAa\,\Big(e^{L\mu}\,(-f1 + e^{L\nu}\, f2 - e^{Lq\nu}\,(f2 + f1\,(-1 + P)) + f1\, P)\,(\nu - \mu)^2\,\mu +\\
&b^2\, f1^2\, f2\, P^2\, pAa^2\,\Big(-e^{L\,(b\, f1\, P\, pAa\, q+\nu)}\,\nu + e^{L\,(q\,(b\, f1\, P\, pAa+\nu-\mu)+\mu)}\,\nu + e^{L\,(\nu+q\mu)}\,\nu -\\
&e^{L\,(q\nu+\mu)}\,\mu + e^{L\,(\nu+\mu)}\,(-\nu + \mu)\Big) - b\, f1\, P\, pAa\,(\nu - \mu)\,\Big(e^{L\,(b\, f1\, P\, pAa\, q+\nu)}\, f2\,\nu -\\
&e^{L\,(\nu+q\mu)}\, f2\,\nu - e^{L\,(q\,(b\, f1\, P\, pAa+\nu-\mu)+\mu)}\,(f2 + f1\,(-1 + P))\,\nu + e^{L\,(\nu+\mu)}\, f2\,(\nu - 2\mu) +\\
&e^{L\,(q\nu+\mu)}\,(2\, f2 + f1\,(-1 + P))\,\mu - e^{L\mu}\, f1\,(-1 + P)\,(-\nu + \mu)\Big)\Big)\Big)\Big/\\
\Big(&e^{L\mu}\,(f1 - e^{L\nu}\, f2 - f1\, P\, pAa + e^{Lq\nu}\,(f2 + f1\,(-1 + P\, pAa)))\,(\nu - \mu)^2\,\mu +\\
&b^2\, f1^2\, f2\, P^2\, pAa^2\\
&\Big(e^{L\,(b\, f1\, P\, pAa\, q+\nu)}\,\nu - e^{L\,(q\,(b\, f1\, P\, pAa+\nu-\mu)+\mu)}\,\nu - e^{L\,(\nu+q\mu)}\,\nu + e^{L\,(\nu+\mu)}\,(\nu - \mu) + e^{L\,(q\nu+\mu)}\,\mu\Big) +\\
&b\, f1\, P\, pAa\,(\nu - \mu)\,\Big(e^{L\,(b\, f1\, P\, pAa\, q+\nu)}\, f2\,\nu - e^{L\,(\nu+q\mu)}\, f2\,\nu -\\
&e^{L\,(q\,(b\, f1\, P\, pAa+\nu-\mu)+\mu)}\,(-f1 + f2 + f1\, P\, pAa)\,\nu + e^{L\,(\nu+\mu)}\, f2\,(\nu - 2\mu) +\\
&e^{L\,(q\nu+\mu)}\,(-f1 + 2\, f2 + f1\, P\, pAa)\,\mu - e^{L\mu}\, f1\,(-1 + P\, pAa)\,(-\nu + \mu)\Big)\Big)\Big)
\end{aligned}$$

For low penetrance, this could be approximated using a first order Taylor approximation by

In[88]:=
```
Clear[πAalowP];
πAalowP[pAa_, P_, b_, f1_, f2_, q_, L_, μ_, ν_] =
 FullSimplify[Normal[Series[πAa[pAa, P, b, f1, f2, q, L, μ, ν], {P, 0, 1}]]]
```

Out[89]= $$pAa + \frac{(-1 + e^{L\,q\,\nu})\ f1\ P\ (-1 + pAa)\ pAa}{-f1 + e^{L\,q\,\nu}\ (f1 - f2) + e^{L\,\nu}\ f2}$$

Which if we ignore sexual mortality would simplify further to

In[90]:=
```
Clear[πAalowPnomort];
πAalowPnomort[pAa_, P_, f1_, f2_, q_] =
 FullSimplify[Limit[πAalowP[pAa, P, b, f1, f2, q, L, μ, ν] , ν → 0]]
```

Out[91]= $$\frac{pAa\ (f2 - f2\ q + f1\ (1 + P\ (-1 + pAa))\ q)}{f2 + f1\ q - f2\ q}$$

With zero penetrance it would become equal to pAa, as there would cease to be any within-colony selection :

In[92]:=
```
πAalowP[pAa, 0, b, f1, f2, q, L, μ, ν]
```

Out[92]= pAa

For example:

In[93]:=
```
πAa[0.5, 0.1, bEst, f1Est, f2Est, qEst, LEst, μEst, νEst] (* low penetrance *)
```

Out[93]= 0.480444

In[94]:=
```
πAa[0.5, 1, bEst, f1Est, f2Est, qEst, LEst, μEst, νEst] (* high penetrance *)
```

Out[94]= 0.192115

In[95]:=
```
πAa[0.5, 0.1, bEst, 1 / 2, 1 / 2, qEst, LEst, μEst, νEst]
(* low penetrance, with f=f1=f2=1/2 *)
```

Out[95]= 0.48403

In[96]:=
```
πAa[0.5, 1, bEst, 1 / 2, 1 / 2, qEst, LEst, μEst, νEst]
(* high penetrance, with f=f1=f2=1/2 *)
```

Out[96]= 0.265506

2.2. Track across-year gene frequency changes across generations using recurrence equations & calculate invasion conditions of a eusociality allele

When the A allele is rare we need to consider these 2 mating types to check if the allele can invade in the population:
M-type colonies : Aa x a,a,...   1/2 Aa, fraction P of these would help/stay before time t=q*L   freq 2.pf
P-type colonies : aa x A,a,a,...   1/me Aa, fraction P of these would help/stay before time t=q*L   freq me.pm

If the frequency of the A gene in female and male gametes is pf and pm, then when rare the frequency of the M and P type mating types are approx. 2.pf and me.pm where me is the effective mating frequency.

Based on the expected productivities of male and female sexuals, as derived by the above ODE system, we can then write down the following recurrence equations:

In[97]:=
```
(* frequencies of M & P type colonies when pf and pm is small *)
fM = 2 * pf;
fP = me * pm;

(* we denote GmM, GmP,
GfM and GfP for the colony productivities in terms of male and female
 dispersing sexuals relative to that of the solitary wild type *)

(* we denote πmM, πmP, πfM and πfP for the proportion of male or female
 sexual offspring carrying the A allele in M or P type colonies *)

(* Now let' s calculate the frequency of the A gene in the next
 generation in male and female gametes pmng and pfng in function of the
 frequency in current generation male and female gametes pm and pf. *)
(* For male gametes: pmng=relative number of A males produced *)
pmng = (fM * GmM * πmM + fP * GmP * πmP);
(* For females: pfng=relative number of Aa females produced/2 *)
pfng = (1 / 2) * (fM * GfM * πfM + fP * GfP * πfP);
```

In[101]:=
```
(* NOTE: to look at invasion of solitary allele A in a
    social wild type population: change ODE so that Gf and Gm are
    defined in terms of prop of offspring with helper genotype &
 prop of offspring with helper genotype in wild type
*)
```

Write this as a gene flow matrix A :

A = gene transition matrix

$$\begin{pmatrix} aff & afm \\ amf & amm \end{pmatrix} = \begin{pmatrix} \text{fem. parent to fem. offspring} & \text{fem. parent to male offspring} \\ \text{male parent to female offspring} & \text{male parent to male offspring} \end{pmatrix}$$

In[102]:=

$$A = \begin{pmatrix} a_{ff} & a_{fm} \\ a_{mf} & a_{mm} \end{pmatrix};$$

Now calculate the dominant eigenvalue λ1 of A :

In[103]:=
```
λ1 = FullSimplify[Eigenvalues[A][[2]]]
```

Out[103]=

$$\frac{1}{2} \left(a_{ff} + \sqrt{4\, a_{fm}\, a_{mf} + (a_{ff} - a_{mm})^2} + a_{mm} \right)$$

Elements aff, afm, amf and amm of matrix A in our case are :

In[104]:=
```
A = A /. {aff -> pfng /. {pf → 1, pm → 0}, afm -> pmng /. {pf → 1, pm → 0},
    amf -> pfng /. {pf → 0, pm → 1}, amm -> pmng /. {pf → 0, pm → 1}};
A // MatrixForm
```

Out[105]//MatrixForm=

$$\begin{pmatrix} \text{GfM}\,\pi\text{fM} & 2\ \text{GmM}\,\pi\text{mM} \\ \frac{\text{GfP me}\,\pi\text{fP}}{2} & \text{GmP me}\,\pi\text{mP} \end{pmatrix}$$

Where given that πmM = 1/2 and πmP = 0 :

In[106]:=
```
A = A /. {πmM → 1 / 2, πmP → 0};
A // MatrixForm
```

Out[107]//MatrixForm=

$$\begin{pmatrix} GfM\,\pi fM & GmM \\ \frac{GfP\,me\,\pi fP}{2} & 0 \end{pmatrix}$$

In which case the dominant eigenvalue λ1 and selection differential s become

In[108]:=
```
λ1 = FullSimplify[Eigenvalues[A][[2]]]
```

Out[108]=

$$\frac{1}{2}\left(GfM\,\pi fM + \sqrt{GfM^2\,\pi fM^2 + 2\,GfP\,GmM\,me\,\pi fP}\right)$$

In[109]:=
```
s = FullSimplify[λ1 - 1]
```

Out[109]=

$$\frac{1}{2}\left(-2 + GfM\,\pi fM + \sqrt{GfM^2\,\pi fM^2 + 2\,GfP\,GmM\,me\,\pi fP}\right)$$

The eusociality allele A can invade when the selection differential s > 0 and when GfP is larger than this critical value :

In[110]:=
```
critGfP = FullSimplify[Solve[s == 0, GfP][[1, 1, 2]]]
```

Out[110]=

$$\frac{2 - 2\,GfM\,\pi fM}{GmM\,me\,\pi fP}$$

Whether or not this condition is easier or harder to satisfy with higher mating frequencies depends on how relative group productivities G scale in function of the proportion of individuals that carry the cooperative helper genotype Aa across different sibships (Olejarz et al. 2015 [13]). Based on the analytical solution of our mechanistically motivated ODE system, it is clear that the traditional prediction is upheld, where eusociality invades less easily under multiple mating:

In[111]:=
```
(* proportion Aa among female offspring in M, P & wild type colonies *)
pAaM = 1 / 2;
pAaP = 1 / me;
pAaW = 0;
```

In[114]:=
```
(* critical GfP needed under single mating,
ancestral f1=f2=0.5 & q=0.5, P=1, me=1 *)
(critGfP /. {GfM -> Gf[pAaM, pAaW, P, b, f1, f2, q, L, μ, ν],
          GmM -> Gm[pAaM, pAaW, P, b, f1, f2, q, L, μ, ν],
           πfM -> πAa[pAaM, P, b, f1, f2, q, L, μ, ν],
          πfP -> πAa[pAaP, P, b, f1, f2, q, L, μ, ν]}) /. {f1 → fAnc,
  f2 → fAnc, q → qAnc, L → LEst, me → 1, ν → νEst, μ → μEst, b → bEst, P → 1}
```

Out[114]= 0.984147

In[115]:=
```
(* critical GfP needed under double mating,
ancestral f1=f2=0.5 & q=0.5, P=1, me=2 *)
(critGfP /. {GfM -> Gf[pAaM, pAaW, P, b, f1, f2, q, L, μ, ν],
          GmM -> Gm[pAaM, pAaW, P, b, f1, f2, q, L, μ, ν],
           πfM -> πAa[pAaM, P, b, f1, f2, q, L, μ, ν],
          πfP -> πAa[pAaP, P, b, f1, f2, q, L, μ, ν]}) /. {f1 → fAnc,
  f2 → fAnc, q → qAnc, L → LEst, me → 2, ν → νEst, μ → μEst, b → bEst, P → 1}
```

Out[115]= 1.35378

In[116]:=
```
(* critical GfP needed under triple mating,
ancestral f1=f2=0.5 & q=0.5, P=1, me=3 *)
(critGfP /. {GfM -> Gf[pAaM, pAaW, P, b, f1, f2, q, L, μ, ν],
          GmM -> Gm[pAaM, pAaW, P, b, f1, f2, q, L, μ, ν],
           πfM -> πAa[pAaM, P, b, f1, f2, q, L, μ, ν],
           πfP -> πAa[pAaP, P, b, f1, f2, q, L, μ, ν]}) /. {f1 → fAnc,
  f2 → fAnc, q → qAnc, L → LEst, me → 3, ν → νEst, μ → μEst, b → bEst, P → 1}
```

Out[116]= 1.45499

Plugging in the analytical solutions for GmM, GmP, GfM, GfP, πfM and πfP based on our mechanistically motivated ODE system then yields the following analytical equation for the selection differential :

In[117]:=
```
S[b_, f1_, f2_, q_, L_, μ_, ν_, me_, P_] = s /. {
   GmM -> Gm[pAaM, pAaW, P, b, f1, f2, q, L, μ, ν],
   (* colony productivities relative to solitary wild type *)
   GmP -> Gm[pAaP, pAaW, P, b, f1, f2, q, L, μ, ν],
   GfM -> Gf[pAaM, pAaW, P, b, f1, f2, q, L, μ, ν],
   GfP -> Gf[pAaP, pAaW, P, b, f1, f2, q, L, μ, ν],
   πfM -> πAa[pAaM, P, b, f1, f2, q, L, μ, ν], (* proportion of dispersing
    female sexual offspring carrying the A allele in M or P type colonies *)
   πfP -> πAa[pAaP, P, b, f1, f2, q, L, μ, ν]
  }
```

Out[117]=

$$\frac{1}{2}\Bigg(-2+\Bigg(e^{-L\mu}\Bigg(-e^{L\mu}\left((-e^{L\nu}+e^{Lq\nu})\,f2+(-1+e^{Lq\nu})\,f1\left(-1+\frac{P}{2}\right)\right)(\nu-\mu)^2\mu-\frac{1}{4}b^2\,f1^2\,f2\,P^2$$
$$\left(e^{L\left(\frac{1}{2}b\,f1\,P\,q+\nu\right)}\nu-e^{L\left(\frac{1}{2}b\,f1\,P\,q+q\nu+\mu-q\mu\right)}\nu-e^{L(\nu+q\mu)}\nu+e^{L(\nu+\mu)}(\nu-\mu)+e^{L(q\nu+\mu)}\mu\right)-$$
$$\frac{1}{2}b\,f1\,P\,(\nu-\mu)\Bigg(e^{L\left(\frac{1}{2}b\,f1\,P\,q+\nu\right)}f2\,\nu-e^{L(\nu+q\mu)}f2\,\nu-$$
$$e^{L\left(\frac{1}{2}b\,f1\,P\,q+q\nu+\mu-q\mu\right)}\left(f2+f1\left(-1+\frac{P}{2}\right)\right)\nu+e^{L(\nu+\mu)}f2\,(\nu-2\mu)+$$
$$e^{L(q\nu+\mu)}\left(2\,f2+f1\left(-1+\frac{P}{2}\right)\right)\mu-e^{L\mu}f1\left(-1+\frac{P}{2}\right)(-\nu+\mu)\Bigg)\Bigg)$$
$$\Bigg(-e^{L\mu}\left((-e^{L\nu}+e^{Lq\nu})\,f2+(-1+e^{Lq\nu})\,f1\,(-1+P)\right)(\nu-\mu)^2\mu-\frac{1}{4}b^2\,f1^2\,f2$$
$$P^2\left(e^{L\left(\frac{1}{2}b\,f1\,P\,q+\nu\right)}\nu-e^{L\left(\frac{1}{2}b\,f1\,P\,q+q\nu+\mu-q\mu\right)}\nu-e^{L(\nu+q\mu)}\nu+e^{L(\nu+\mu)}(\nu-\mu)+e^{L(q\nu+\mu)}\mu\right)-$$
$$\frac{1}{2}b\,f1\,P\,(\nu-\mu)\Bigg(e^{L\left(\frac{1}{2}b\,f1\,P\,q+\nu\right)}f2\,\nu-e^{L(\nu+q\mu)}f2\,\nu-$$
$$e^{L\left(\frac{1}{2}b\,f1\,P\,q+q\nu+\mu-q\mu\right)}(f2+f1\,(-1+P))\,\nu+e^{L(\nu+\mu)}f2\,(\nu-2\mu)+$$
$$e^{L(q\nu+\mu)}(2\,f2+f1\,(-1+P))\,\mu-e^{L\mu}f1\,(-1+P)\,(-\nu+\mu)\Bigg)\Bigg)\Bigg)\Bigg/$$
$$\Bigg(2\left(-(-1+e^{Lq\nu})\,f1+(-e^{L\nu}+e^{Lq\nu})\,f2\right)(\nu-\mu)\left(\frac{b\,f1\,P}{2}+\nu-\mu\right)\left(-\frac{1}{2}b\,f1\,P+\mu\right)$$
$$\Bigg(e^{L\mu}\left((-e^{L\nu}+e^{Lq\nu})\,f2+(-1+e^{Lq\nu})\,f1\left(-1+\frac{P}{2}\right)\right)(\nu-\mu)^2\mu+\frac{1}{4}b^2\,f1^2\,f2\,P^2$$
$$\left(e^{L\left(\frac{1}{2}b\,f1\,P\,q+\nu\right)}\nu-e^{L\left(\frac{1}{2}b\,f1\,P\,q+q\nu+\mu-q\mu\right)}\nu-e^{L(\nu+q\mu)}\nu+e^{L(\nu+\mu)}(\nu-\mu)+e^{L(q\nu+\mu)}\mu\right)+$$
$$\frac{1}{2}b\,f1\,P\,(\nu-\mu)\Bigg(e^{L\left(\frac{1}{2}b\,f1\,P\,q+\nu\right)}f2\,\nu-e^{L(\nu+q\mu)}f2\,\nu-$$

$$e^{L\left(\frac{1}{2} b\, f1\, P\, q+q\, \nu+\mu-q\, \mu\right)} \left(f2 + f1 \left(-1 + \frac{P}{2}\right)\right) \nu + e^{L\,(\nu+\mu)}\, f2\, (\nu - 2\,\mu) +$$

$$e^{L\,(q\,\nu+\mu)} \left(2\, f2 + f1 \left(-1 + \frac{P}{2}\right)\right) \mu - e^{L\,\mu}\, f1 \left(-1 + \frac{P}{2}\right) (-\nu + \mu)\Big)\Big)\Big) +$$

$$\sqrt{\vphantom{x}}\Big(\Big(\Big(e^{-2\,L\,\mu} \Big(-e^{L\,\mu} \Big((-e^{L\,\nu} + e^{L\,q\,\nu})\, f2 + (-1 + e^{L\,q\,\nu})\, f1 \left(-1 + \frac{P}{2}\right)\Big) (\nu - \mu)^2\, \mu -$$

$$\frac{1}{4}\, b^2\, f1^2\, f2\, P^2 \Big(e^{L\left(\frac{1}{2} b\, f1\, P\, q+\nu\right)}\, \nu - e^{L\left(\frac{1}{2} b\, f1\, P\, q+q\, \nu+\mu-q\, \mu\right)}\, \nu - e^{L\,(\nu+q\,\mu)}\, \nu +$$

$$e^{L\,(\nu+\mu)}\, (\nu - \mu) + e^{L\,(q\,\nu+\mu)}\, \mu\Big) - \frac{1}{2}\, b\, f1\, P\, (\nu - \mu) \Big(e^{L\left(\frac{1}{2} b\, f1\, P\, q+\nu\right)}\, f2\, \nu -$$

$$e^{L\,(\nu+q\,\mu)}\, f2\, \nu - e^{L\left(\frac{1}{2} b\, f1\, P\, q+q\, \nu+\mu-q\, \mu\right)} \left(f2 + f1 \left(-1 + \frac{P}{2}\right)\right) \nu + e^{L\,(\nu+\mu)}\, f2$$

$$(\nu - 2\,\mu) + e^{L\,(q\,\nu+\mu)} \left(2\, f2 + f1 \left(-1 + \frac{P}{2}\right)\right) \mu - e^{L\,\mu}\, f1 \left(-1 + \frac{P}{2}\right) (-\nu + \mu)\Big)\Big)^2$$

$$\Big(-e^{L\,\mu}\, ((-e^{L\,\nu} + e^{L\,q\,\nu})\, f2 + (-1 + e^{L\,q\,\nu})\, f1\, (-1 + P))\, (\nu - \mu)^2\, \mu - \frac{1}{4}\, b^2\, f1^2$$

$$f2\, P^2 \Big(e^{L\left(\frac{1}{2} b\, f1\, P\, q+\nu\right)}\, \nu - e^{L\left(\frac{1}{2} b\, f1\, P\, q+q\, \nu+\mu-q\, \mu\right)}\, \nu - e^{L\,(\nu+q\,\mu)}\, \nu + e^{L\,(\nu+\mu)}\, (\nu - \mu) +$$

$$e^{L\,(q\,\nu+\mu)}\, \mu\Big) - \frac{1}{2}\, b\, f1\, P\, (\nu - \mu) \Big(e^{L\left(\frac{1}{2} b\, f1\, P\, q+\nu\right)}\, f2\, \nu - e^{L\,(\nu+q\,\mu)}\, f2\, \nu -$$

$$e^{L\left(\frac{1}{2} b\, f1\, P\, q+q\, \nu+\mu-q\, \mu\right)}\, (f2 + f1\, (-1 + P))\, \nu + e^{L\,(\nu+\mu)}\, f2\, (\nu - 2\,\mu) +$$

$$e^{L\,(q\,\nu+\mu)}\, (2\, f2 + f1\, (-1 + P))\, \mu - e^{L\,\mu}\, f1\, (-1 + P)\, (-\nu + \mu)\Big)\Big)^2\Big) \Big/$$

$$\Big(4\, (-(-1 + e^{L\,q\,\nu})\, f1 + (-e^{L\,\nu} + e^{L\,q\,\nu})\, f2)^2\, (\nu - \mu)^2 \left(\frac{b\, f1\, P}{2} + \nu - \mu\right)^2$$

$$\left(-\frac{1}{2}\, b\, f1\, P + \mu\right)^2 \Big(e^{L\,\mu} \Big((-e^{L\,\nu} + e^{L\,q\,\nu})\, f2 + (-1 + e^{L\,q\,\nu})\, f1 \left(-1 + \frac{P}{2}\right)\Big) (\nu - \mu)^2\, \mu +$$

$$\frac{1}{4}\, b^2\, f1^2\, f2\, P^2 \Big(e^{L\left(\frac{1}{2} b\, f1\, P\, q+\nu\right)}\, \nu - e^{L\left(\frac{1}{2} b\, f1\, P\, q+q\, \nu+\mu-q\, \mu\right)}\, \nu - e^{L\,(\nu+q\,\mu)}\, \nu + e^{L\,(\nu+\mu)}$$

$$(\nu - \mu) + e^{L\,(q\,\nu+\mu)}\, \mu\Big) + \frac{1}{2}\, b\, f1\, P\, (\nu - \mu) \Big(e^{L\left(\frac{1}{2} b\, f1\, P\, q+\nu\right)}\, f2\, \nu - e^{L\,(\nu+q\,\mu)}$$

$$f2\, \nu - e^{L\left(\frac{1}{2} b\, f1\, P\, q+q\, \nu+\mu-q\, \mu\right)} \left(f2 + f1 \left(-1 + \frac{P}{2}\right)\right) \nu + e^{L\,(\nu+\mu)}\, f2\, (\nu - 2\,\mu) +$$

$$e^{L\,(q\,\nu+\mu)} \left(2\, f2 + f1 \left(-1 + \frac{P}{2}\right)\right) \mu - e^{L\,\mu}\, f1 \left(-1 + \frac{P}{2}\right) (-\nu + \mu)\Big)\Big)^2\Big) -$$

$$\Big(2\, e^{-2\,L\,\mu} \Big(-e^{L\,\mu}\, (1 - f1 + e^{L\,q\,\nu}\, (f1 - f2) + e^{L\,\nu}\, (-1 + f2))\, (\nu - \mu)^2\, \mu +$$

$$\frac{1}{4}\, b^2\, f1^2\, (-1 + f2)\, P^2 \Big(e^{L\left(\frac{1}{2} b\, f1\, P\, q+\nu\right)}\, \nu - e^{L\left(\frac{1}{2} b\, f1\, P\, q+q\, \nu+\mu-q\, \mu\right)}\, \nu - e^{L\,(\nu+q\,\mu)}\, \nu + e^{L\,(\nu+\mu)}$$

$$(\nu - \mu) + e^{L\,(q\,\nu+\mu)}\, \mu\Big) + \frac{1}{2}\, b\, f1\, P\, (\nu - \mu) \Big(e^{L\left(\frac{1}{2} b\, f1\, P\, q+q\, \nu+\mu-q\, \mu\right)}\, (f1 - f2)\, \nu +$$

$$e^{L\left(\frac{1}{2} b\, f1\, P\, q+\nu\right)}\, (-1 + f2)\, \nu - e^{L\,(\nu+q\,\mu)}\, (-1 + f2)\, \nu + e^{L\,(\nu+\mu)}\, (-1 + f2)\, (\nu - 2\,\mu) -$$

$$e^{L\,(q\,\nu+\mu)}\, (1 + f1 - 2\, f2)\, \mu + e^{L\,\mu}\, (-1 + f1)\, (-\nu + \mu)\Big)\Big)$$

$$\Big(-e^{L\,\mu}\, ((-e^{L\,\nu} + e^{L\,q\,\nu})\, f2 + (-1 + e^{L\,q\,\nu})\, f1\, (-1 + P))\, (\nu - \mu)^2\, \mu - \frac{1}{me^2} b^2\, f1^2\, f2$$

$$P^2 \left(e^{L\left(\frac{b\,f1\,P\,q}{me}+\nu\right)}\,\nu - e^{L\left(\frac{b\,f1\,P\,q}{me}+q\,\nu+\mu-q\,\mu\right)}\,\nu - e^{L\,(\nu+q\,\mu)}\,\nu + e^{L\,(\nu+\mu)}\,(\nu-\mu) + e^{L\,(q\,\nu+\mu)}\,\mu\right) -$$

$$\frac{1}{me} b\,f1\,P\,(\nu-\mu)\left(e^{L\left(\frac{b\,f1\,P\,q}{me}+\nu\right)}\,f2\,\nu - e^{L\,(\nu+q\,\mu)}\,f2\,\nu -\right.$$

$$e^{L\left(\frac{b\,f1\,P\,q}{me}+q\,\nu+\mu-q\,\mu\right)}\,(f2+f1\,(-1+P))\,\nu + e^{L\,(\nu+\mu)}\,f2\,(\nu-2\,\mu) +$$

$$\left.e^{L\,(q\,\nu+\mu)}\,(2\,f2+f1\,(-1+P))\,\mu - e^{L\,\mu}\,f1\,(-1+P)\,(-\nu+\mu)\right)\Bigg)$$

$$\left(-e^{L\,\mu}\left((-e^{L\,\nu}+e^{L\,q\,\nu})\,f2 + (-1+e^{L\,q\,\nu})\,f1\left(-1+\frac{P}{me}\right)\right)(\nu-\mu)^2\,\mu - \frac{1}{me^2} b^2\,f1^2\,f2\right.$$

$$P^2 \left(e^{L\left(\frac{b\,f1\,P\,q}{me}+\nu\right)}\,\nu - e^{L\left(\frac{b\,f1\,P\,q}{me}+q\,\nu+\mu-q\,\mu\right)}\,\nu - e^{L\,(\nu+q\,\mu)}\,\nu + e^{L\,(\nu+\mu)}\,(\nu-\mu) + e^{L\,(q\,\nu+\mu)}\,\mu\right) -$$

$$\frac{1}{me} b\,f1\,P\,(\nu-\mu)\left(e^{L\left(\frac{b\,f1\,P\,q}{me}+\nu\right)}\,f2\,\nu - e^{L\,(\nu+q\,\mu)}\,f2\,\nu -\right.$$

$$e^{L\left(\frac{b\,f1\,P\,q}{me}+q\,\nu+\mu-q\,\mu\right)}\left(f2+f1\left(-1+\frac{P}{me}\right)\right)\nu + e^{L\,(\nu+\mu)}\,f2\,(\nu-2\,\mu) +$$

$$\left.\left.e^{L\,(q\,\nu+\mu)}\left(2\,f2+f1\left(-1+\frac{P}{me}\right)\right)\mu - e^{L\,\mu}\,f1\left(-1+\frac{P}{me}\right)(-\nu+\mu)\right)\right)\Bigg/$$

$$\Bigg((1-f1+e^{L\,q\,\nu}\,(f1-f2)+e^{L\,\nu}\,(-1+f2))\,(-(-1+e^{L\,q\,\nu})\,f1+(-e^{L\,\nu}+e^{L\,q\,\nu})\,f2)$$

$$(\nu-\mu)^2\left(\frac{b\,f1\,P}{2}+\nu-\mu\right)\left(\frac{b\,f1\,P}{me}+\nu-\mu\right)\left(-\frac{1}{2}\,b\,f1\,P+\mu\right)\left(-\frac{b\,f1\,P}{me}+\mu\right)$$

$$\left(e^{L\,\mu}\left((-e^{L\,\nu}+e^{L\,q\,\nu})\,f2+(-1+e^{L\,q\,\nu})\,f1\left(-1+\frac{P}{me}\right)\right)(\nu-\mu)^2\,\mu + \frac{1}{me^2} b^2\,f1^2\,f2\,P^2\right.$$

$$\left(e^{L\left(\frac{b\,f1\,P\,q}{me}+\nu\right)}\,\nu - e^{L\left(\frac{b\,f1\,P\,q}{me}+q\,\nu+\mu-q\,\mu\right)}\,\nu - e^{L\,(\nu+q\,\mu)}\,\nu + e^{L\,(\nu+\mu)}\,(\nu-\mu) + e^{L\,(q\,\nu+\mu)}\,\mu\right) +$$

$$\frac{1}{me} b\,f1\,P\,(\nu-\mu)\left(e^{L\left(\frac{b\,f1\,P\,q}{me}+\nu\right)}\,f2\,\nu - e^{L\,(\nu+q\,\mu)}\,f2\,\nu -\right.$$

$$e^{L\left(\frac{b\,f1\,P\,q}{me}+q\,\nu+\mu-q\,\mu\right)}\left(f2+f1\left(-1+\frac{P}{me}\right)\right)\nu + e^{L\,(\nu+\mu)}\,f2\,(\nu-2\,\mu) +$$

$$\left.\left.e^{L\,(q\,\nu+\mu)}\left(2\,f2+f1\left(-1+\frac{P}{me}\right)\right)\mu - e^{L\,\mu}\,f1\left(-1+\frac{P}{me}\right)(-\nu+\mu)\right)\right)\Bigg)$$

The social allele A can invade & spread when the selection differential S > 0 :

In[118]:=
```
spread[b_, f1_, f2_, q_, L_, μ_, ν_, me_, P_] =
  If[S[b, f1, f2, q, L, μ, ν, me, P] > 0, 1, 0];
```

2.3. Invasion conditions of the eusociality allele under weak selection

Under weak selection, with low penetrance *P*, and assuming that ancestrally *f1 = f2 = fAnc=1/2*, the selection differential S simplifies to

In[119]:=
```
SlowP = FullSimplify[Normal[Series[S[b, 1 / 2, 1 / 2, q, L, μ, ν, me, P], {P, 0, 1}]]]
 (* this is using a first order Taylor approximation around P=0 *)
```

Out[119]=

$$\frac{e^{-L\,\mu}\,P\,\left(-4\,e^{L\,\mu}\,(-1+e^{L\,q\,\nu})\,me\,\mu\,(-\nu+\mu) + b\,(1+me)\,\left(e^{L\,\nu}\,\nu - e^{L\,(\nu+q\,\mu)}\,\nu - e^{L\,\mu}\,\mu + e^{L\,(q\,\nu+\mu)}\,\mu\right)\right)}{6\,(-1+e^{L\,\nu})\,me\,\mu\,(-\nu+\mu)}$$

In that case, invasion of the eusociality allele is possible under vanishly weak selection when *b* is larger than this critical value :

In[120]:=
```
critblowP[q_, L_, μ_, ν_, me_] = FullSimplify[Solve[SlowP == 0, b] [[1, 1, 2]],
  μ > 0 && ν > 0 && me ≥ 1 && f > 0 && f < 1 && q > 0 && q < 1 && L > 0]
```

Out[120]=
$$\frac{4\,e^{L\,\mu}\,(-1+e^{L\,q\,\nu})\,me\,\mu\,(-\nu+\mu)}{(1+me)\,(e^{L\,\nu}\,\nu-e^{L\,(\nu+q\,\mu)}\,\nu-e^{L\,\mu}\,\mu+e^{L\,(q\,\nu+\mu)}\,\mu)}$$

We can see that the required critical birth rate is higher under double mating than under single mating :

In[121]:=
```
critblowP[qAnc, LEst, μEst, νEst, 1]
```

Out[121]= 0.0912764

In[122]:=
```
critblowP[qAnc, LEst, μEst, νEst, 2]
```

Out[122]= 0.121702

If we would ignore mortality (μ = ν = 0), this would further simplify to

In[123]:=
```
critblowPnomort[q_, L_, me_] =
  FullSimplify[Limit[Limit[critblowP[q, L, μ, ν, me], μ → 0], ν → 0]]
```

Out[123]=
$$-\frac{8\,me}{L\,(1+me)\,(-2+q)}$$

For q=1/2 we would then get

In[124]:=
```
critblowPnomort[1 / 2, L, me]
```

Out[124]=
$$\frac{16\,me}{3\,L\,(1+me)}$$

While for q = 0 we would get

In[125]:=
```
critblowPnomort[0, L, me]
```

Out[125]=
$$\frac{4\,me}{L\,(1+me)}$$

which implies that b.L.Rsib > 1

In[126]:=
```
Rbrother = 1 / 4;
Rsister[me_] = (1 / 4) + 1 / (2 * me);
Rsib[me_, f_] = FullSimplify[(1 - f) * Rbrother + f * Rsister[me]]
```

Out[128]=
$$\frac{2\,f+me}{4\,me}$$

In[129]:=
```
Rsib[me, 1 / 2]
```

Out[129]=
$$\frac{1+me}{4\,me}$$

2.4. Plots of selection differential and invasion conditions, assuming an ancestral 1 : 1 investment sex ratio throughout the season (f1 = f2 = fEst)

Set some options for countour plots of selection differentials :

```
In[130]:= (*Define the number of contours and their range*)
maxS = 0.1;
maxSlegend = 0.11; (* Slightly higher to show red clipped part in legend *)
minS = 0;
minSlegend = -0.01;
(* Slightly lower to show black clipped part in legend *)
ncontours = 200;
contourValues = Table[i, {i, 0, maxS, maxS / ncontours}];
nrsteps = 30; (* increase nr of steps to make plots look nicer *)
colpal = "Rainbow";
clipcol1 = Black; (* for region where allele cannot invade *)
clipcol2 = Red; (* for region where selection differential S > maxS *)
(* Define a normalized color function for plots *)
plotColorFunction = Function[{z}, ColorData[colpal][Rescale[z, {minS, maxS}]]];
(* Define a separate color function for the legend *)
legendColorFunction =
  Function[{z}, Module[{scaledZ = (z * (maxSlegend - minSlegend) + minSlegend)},
    (* Scale z to the full range of the legend *)
    Piecewise[{
      {clipcol1, scaledZ < minS},
      {ColorData[colpal][(scaledZ) / (maxSlegend - minSlegend)],
       minS ≤ scaledZ ≤ maxS},
      {clipcol2, scaledZ > maxS}}]]];
```

Calculate the critical b that would be needed for M type colonies to produce at least nwM workers :

```
In[142]:= critbworkerprod[f1_, q_, L_, μ_, P_, nwM_ ] :=
 FindRoot[Wtot[0.5, P, b, f1, q, L, μ] == nwM, {b, 0.1}][[1, 2]]

In[143]:= critbworkerprod[fAnc, qEst, LEst, μEst, 0.1, 1]

Out[143]= 0.484095

In[144]:= critbworkerprod[fAnc, qEst, LEst, μEst, 1, 1]

Out[144]= 0.0484095
```

Calculate the critical b that would be needed to get a given minimum selection coefficient S (e.g. S=0.01, which is the selection coefficient you would approximately need to overcome drift in a population with an effective population size Ne=100, which is a common value for Ne in both solitary and social Hymenoptera, Zayed 2004 [15] & Dyson et al. 2021 [16]) :

```
In[145]:= critbminS[f1_, f2_, q_, L_, μ_, ν_, me_, P_ , minS_] :=
 FindRoot[S[b, f1, f2, q, L, μ, ν, me, P] == minS, {b, 0.1}][[1, 2]]

In[146]:= critbminS[fAnc, fAnc, 0.001, LEst, μEst, νEst, 1, 1 , 0.01]

Out[146]= 2.10297

In[147]:= critbminS[fAnc, fAnc, qEst, LEst, μEst, νEst, 1, 1 , 0.01]

Out[147]= 0.099871

In[148]:= critbminS[fAnc, fAnc, qEst, LEst, μEst, νEst, 1, 0.1 , 0.01]

Out[148]= 0.1187
```

Plot selection differentials for a dominant allele causing the expression of helping behaviour with probability equal to the penetrance parameter P for various mating frequencies (1-3) me :

In[149]:=
```
f1val = fAnc; (* ancestrally assumed =1/2 *)
f2val = fAnc;
bmin = bEst / 2;
bmax = bEst * 2;
minSval = 0.01; (* contour to plot as a white
  line for a minimum relevant selection coefficient S=0.01,
which would be approx. the selection coefficient
  needed to overpower drift with Ne=100 *)

(* plots of selection differential for low penetrance,
P=0.1, with single, double or triple mating *)
cp1 = ContourPlot[S[b, f1val, f2val, q, LEst, μEst, νEst, 1, 0.1],
   {q, 0, 1}, {b, bmin, bmax}, PlotPoints → nrsteps,
   PlotRange → {minS, maxS}, ClippingStyle → {clipcol1, clipcol2},
   ContourLines → False, ColorFunctionScaling → False,
   Contours → contourValues, ColorFunction → plotColorFunction,
   PlotLabel → Row[{Style[Subscript["m", "e"], Italic],
      "=1, ", Style["P", Italic], "=0.1"}] ];
cp2 = ContourPlot[S[b, f1val, f2val, q, LEst, μEst, νEst, 2, 0.1],
   {q, 0, 1}, {b, bmin, bmax}, PlotPoints → nrsteps,
   PlotRange → {minS, maxS}, ClippingStyle → {clipcol1, clipcol2},
   ContourLines → False, ColorFunctionScaling → False,
   Contours → contourValues, ColorFunction → plotColorFunction,
   PlotLabel → Row[{Style[Subscript["m", "e"], Italic],
      "=2, ", Style["P", Italic], "=0.1"}]];
cp3 = ContourPlot[S[b, f1val, f2val, q, LEst, μEst, νEst, 3, 0.1],
   {q, 0, 1}, {b, bmin, bmax}, PlotPoints → nrsteps,
   PlotRange → {minS, maxS}, ClippingStyle → {clipcol1, clipcol2},
   ContourLines → False, ColorFunctionScaling → False,
   Contours → contourValues, ColorFunction → plotColorFunction,
   PlotLabel → Row[{Style[Subscript["m", "e"], Italic],
      "=3, ", Style["P", Italic], "=0.1"}]];
(* plots of selection differential for high penetrance, P=1,
with single, double or triple mating *)
cp4 = ContourPlot[S[b, f1val, f2val, q, LEst, μEst, νEst, 1, 1],
   {q, 0, 1}, {b, bmin, bmax}, PlotPoints → nrsteps,
   PlotRange → {minS, maxS}, ClippingStyle → {clipcol1, clipcol2},
   ContourLines → False, ColorFunctionScaling → False,
   Contours → contourValues, ColorFunction → plotColorFunction, PlotLabel → Row[
     {Style[Subscript["m", "e"], Italic], "=1, ", Style["P", Italic], "=1"}]];
cp5 = ContourPlot[S[b, f1val, f2val, q, LEst, μEst, νEst, 2, 1],
   {q, 0, 1}, {b, bmin, bmax}, PlotPoints → nrsteps,
   PlotRange → {minS, maxS}, ClippingStyle → {clipcol1, clipcol2},
   ContourLines → False, ColorFunctionScaling → False,
   Contours → contourValues, ColorFunction → plotColorFunction, PlotLabel → Row[
     {Style[Subscript["m", "e"], Italic], "=2, ", Style["P", Italic], "=1"}]];
cp6 = ContourPlot[S[b, f1val, f2val, q, LEst, μEst, νEst, 3, 1],
   {q, 0, 1}, {b, bmin, bmax}, PlotPoints → nrsteps,
   PlotRange → {minS, maxS}, ClippingStyle → {clipcol1, clipcol2},
   ContourLines → False, ColorFunctionScaling -> False,
   Contours → contourValues, ColorFunction → plotColorFunction, PlotLabel → Row[
     {Style[Subscript["m", "e"], Italic], "=3, ", Style["P", Italic], "=1"}]];
(* calculate contours with critical b for low S=
```

```
 0.01 & show them as a white line *)
lp1a = Plot[critbminS[f1val, f2val, q, LEst, μEst, νEst, 1, 0.1 , minSval],
   {q, 0, 1}, PlotStyle → {White, Thickness[0.004]},
   PlotPoints → nrsteps * 10, PlotRange → {{0, 1}, {bmin, bmax}}];
lp2a = Plot[critbminS[f1val, f2val, q, LEst, μEst, νEst, 2, 0.1 , minSval],
   {q, 0, 1}, PlotStyle → {White, Thickness[0.004]},
   PlotPoints → nrsteps * 10, PlotRange → {{0, 1}, {bmin, bmax}}];
lp3a = Plot[critbminS[f1val, f2val, q, LEst, μEst, νEst, 3, 0.1 , minSval],
   {q, 0, 1}, PlotStyle → {White, Thickness[0.004]},
   PlotPoints → nrsteps * 10, PlotRange → {{0, 1}, {bmin, bmax}}];
lp4a = Plot[critbminS[f1val, f2val, q, LEst, μEst, νEst, 1, 1 , minSval], {q, 0, 1},
   PlotStyle → {White, Thin},
   PlotPoints → nrsteps * 10, PlotRange → {{0, 1}, {bmin, bmax}}];
lp5a = Plot[critbminS[f1val, f2val, q, LEst, μEst, νEst, 2, 1 , minSval],
   {q, 0, 1}, PlotStyle → {White, Thickness[0.004]},
   PlotPoints → nrsteps * 10, PlotRange → {{0, 1}, {bmin, bmax}}];
lp6a = Plot[critbminS[f1val, f2val, q, LEst, μEst, νEst, 3, 1 , minSval], {q, 0, 1},
   PlotStyle → {White, Thin},
   PlotPoints → nrsteps * 10, PlotRange → {{0, 1}, {bmin, bmax}}];
(* calculate contours with critical b for which 10% of all
 M type colonies would produce at least 1 worker *)
lp1b = Plot[critbworkerprod[f1val, q, LEst, μEst, 0.1, 0.1],
   {q, 0, 1}, PlotStyle → {White, Thin, Dashed},
   PlotPoints → nrsteps * 10, PlotRange → {{0, 1}, {bmin, bmax}}];
lp2b = Plot[critbworkerprod[f1val, q, LEst, μEst, 0.1, 0.1],
   {q, 0, 1}, PlotStyle → {White, Thin, Dashed},
   PlotPoints → nrsteps * 10, PlotRange → {{0, 1}, {bmin, bmax}}];
lp3b = Plot[critbworkerprod[f1val, q, LEst, μEst, 0.1, 0.1],
   {q, 0, 1}, PlotStyle → {White, Thin, Dashed},
   PlotPoints → nrsteps * 10, PlotRange → {{0, 1}, {bmin, bmax}}];
lp4b = Plot[critbworkerprod[f1val, q, LEst, μEst, 1, 0.1], {q, 0, 1},
   PlotStyle → {White, Thin, Dashed},
   PlotPoints → nrsteps * 10, PlotRange → {{0, 1}, {bmin, bmax}}];
lp5b = Plot[critbworkerprod[f1val, q, LEst, μEst, 1, 0.1],
   {q, 0, 1}, PlotStyle → {White, Thin, Dashed},
   PlotPoints → nrsteps * 10, PlotRange → {{0, 1}, {bmin, bmax}}];
lp6b = Plot[critbworkerprod[f1val, q, LEst, μEst, 1, 0.1], {q, 0, 1},
   PlotStyle → {White, Thin, Dashed},
   PlotPoints → nrsteps * 10, PlotRange → {{0, 1}, {bmin, bmax}}];
(* add empirically measured values for P. gallicus as a + on the plot *)
pointPgallicus = ListPlot[{{qEst, bEst}},
   PlotMarkers → Style["+", {Orange, Bold, FontSize → 10}], PlotLabel → None ];
linebPgallicus = Plot[bEst, {q, 0, 1}, PlotStyle → {Orange, Thickness[0.008]}];
gg = GraphicsGrid[ {{
     Show[cp1, lp1a, lp1b, linebPgallicus, pointPgallicus],
     Show[cp2, lp2a, lp2b, linebPgallicus, pointPgallicus],
     Show[cp3, lp3a, lp3b, linebPgallicus, pointPgallicus]}, {
     Show[cp4, lp4a, lp4b, linebPgallicus, pointPgallicus],
     Show[cp5, lp5a, lp5b, linebPgallicus, pointPgallicus],
     Show[cp6, lp6a, lp6b, linebPgallicus, pointPgallicus]}} ,
   ImageSize → 800];
legend = BarLegend[{legendColorFunction, {minSlegend, maxSlegend}},
   LegendLabel → Style["S", Italic, FontSize → 12],
```

```
   "StyledContours" → {{minSval, Directive[White]}}];
multipanel1 =
 Row[{Labeled[gg, {Row[{"Per capita productivity (", Style["b", Italic], ")"}],

    Row[{"Switching point (", Style["q", Italic], ")"}]}, {Left, Bottom},
   LabelStyle → Directive[FontFamily → "Helvetica"], RotateLabel → True,
   ImageSize → 800], Spacer[10], legend}, Alignment → Right]
```

Out[176]=

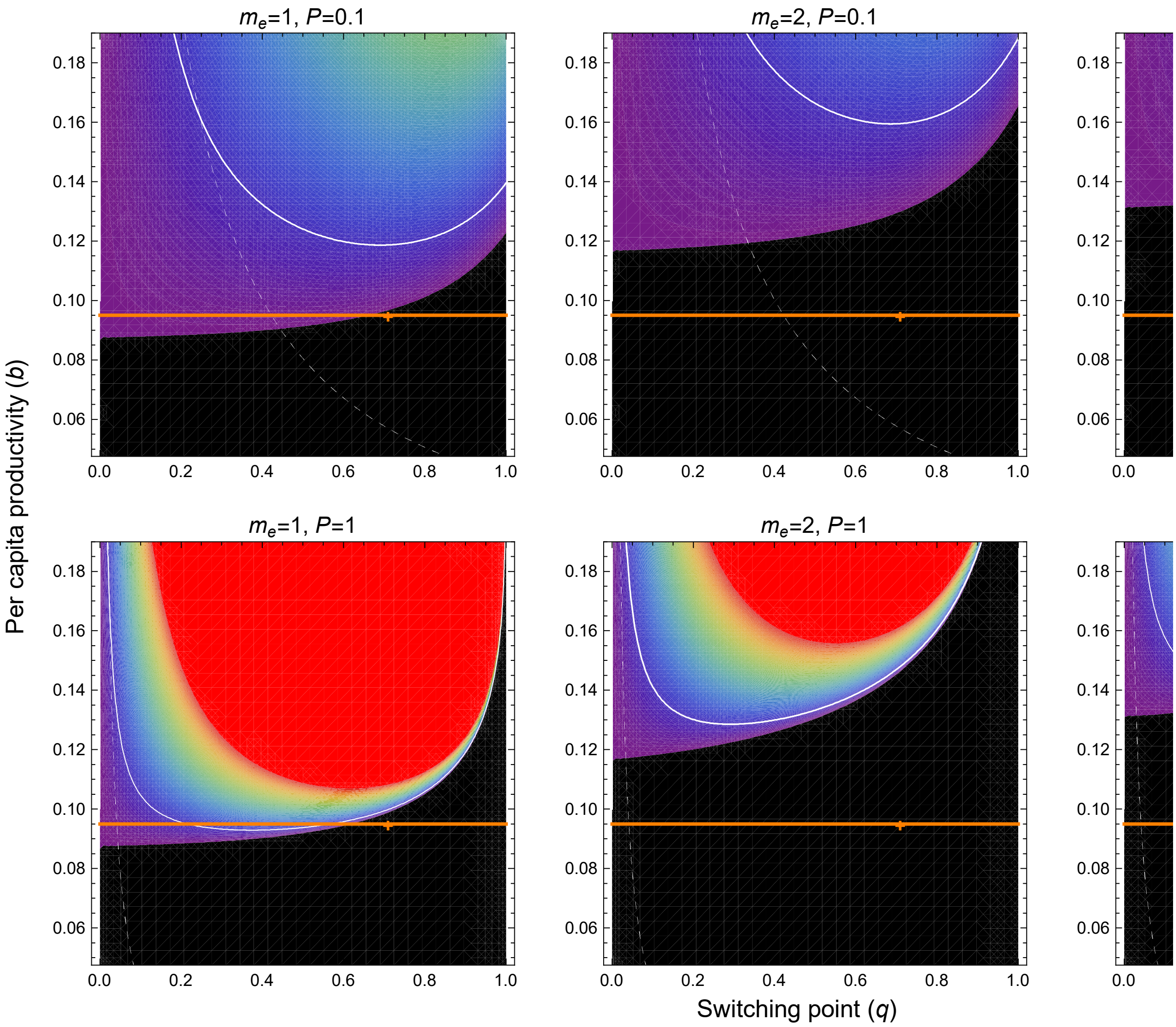

In general, for any strength of selection, the critical per capita birth rate b that needs to be exceeded for the eusociality allele to be able to invade can be solved numerically as follows :

In[177]:=
```
critb[f1_, f2_, q_, L_, μ_, ν_, me_, P_] :=
 FindRoot[S[b, f1, f2, q, L, μ, ν, me, P] == 0, {b, 0.1}][[1, 2]]
```

We can plot the required critical value of b in function of the switch point q for different mating frequencies and either low (P = 0.1) or high penetrance (P = 1) :

In[178]:=
```
Plot[{critb[fAnc, fAnc, q, LEst, μEst, νEst, 1, 0.1],
  critb[fAnc, fAnc, q, LEst, μEst, νEst, 2, 0.1],
  critb[fAnc, fAnc, q, LEst, μEst, νEst, 3, 0.1],
  critb[fAnc, fAnc, q, LEst, μEst, νEst, 1, 1],
  critb[fAnc, fAnc, q, LEst, μEst, νEst, 2, 1],
  critb[fAnc, fAnc, q, LEst, μEst, νEst, 3, 1]},
 {q, 0, 1}, Frame -> True, PlotLabel → None,
 PlotStyle → {{Blue}, {Darker[Green]}, {Red},
   {Blue, Dashed}, {Darker[Green], Dashed}, {Red, Dashed}},
 FrameLabel -> {"Switch point (q)", "Per capita productivity (b)"}]
```

Out[178]=

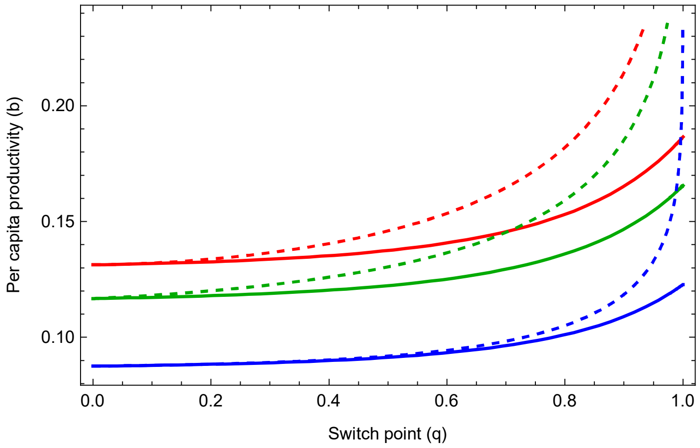

2.5. Plots of selection differential and invasion conditions, for the derived case where f1=1 and f2=f2Est (i.e. with all-female early season allocation and female-biased late season allocation)

Plot selection differentials for f1=1 & f2=f2Est - the empirically measured values observed in *P. gallicus* :

In[179]:=
```
f1val = f1Est; (* = 1 *)
f2val = f2Est;
bmin = bEst / 2;
bmax = bEst * 2;
minSval = 0.01; (* contour to plot as a white
  line for a minimum relevant selection coefficient S=0.01,
which would be approx. the selection coefficient
  needed to overpower drift with Ne=100 *)

(* plots of selection differential for low penetrance,
P=0.1, with single, double or triple mating *)
cp1 = ContourPlot[S[b, f1val, f2val, q, LEst, μEst, νEst, 1, 0.1],
   {q, 0, 1}, {b, bmin, bmax}, PlotPoints → nrsteps,
   PlotRange → {minS, maxS}, ClippingStyle → {clipcol1, clipcol2},
   ContourLines → False, ColorFunctionScaling → False,
   Contours → contourValues, ColorFunction → plotColorFunction,
   PlotLabel → Row[{Style[Subscript["m", "e"], Italic],
      "=1, ", Style["P", Italic], "=0.1"}] ];
cp2 = ContourPlot[S[b, f1val, f2val, q, LEst, μEst, νEst, 2, 0.1],
   {q, 0, 1}, {b, bmin, bmax}, PlotPoints → nrsteps,
   PlotRange → {minS, maxS}, ClippingStyle → {clipcol1, clipcol2},
```

```
    ContourLines → False, ColorFunctionScaling → False,
    Contours → contourValues, ColorFunction → plotColorFunction,
    PlotLabel → Row[{Style[Subscript["m", "e"], Italic],
       "=2, ", Style["P", Italic], "=0.1"}]];
cp3 = ContourPlot[S[b, f1val, f2val, q, LEst, μEst, νEst, 3, 0.1],
    {q, 0, 1}, {b, bmin, bmax}, PlotPoints → nrsteps,
    PlotRange → {minS, maxS}, ClippingStyle → {clipcol1, clipcol2},
    ContourLines → False, ColorFunctionScaling → False,
    Contours → contourValues, ColorFunction → plotColorFunction,
    PlotLabel → Row[{Style[Subscript["m", "e"], Italic],
       "=3, ", Style["P", Italic], "=0.1"}]];
(* plots of selection differential for high penetrance, P=1,
with single, double or triple mating *)
cp4 = ContourPlot[S[b, f1val, f2val, q, LEst, μEst, νEst, 1, 1],
    {q, 0, 1}, {b, bmin, bmax}, PlotPoints → nrsteps,
    PlotRange → {minS, maxS}, ClippingStyle → {clipcol1, clipcol2},
    ContourLines → False, ColorFunctionScaling → False,
    Contours → contourValues, ColorFunction → plotColorFunction, PlotLabel → Row[
      {Style[Subscript["m", "e"], Italic], "=1, ", Style["P", Italic], "=1"}]];
cp5 = ContourPlot[S[b, f1val, f2val, q, LEst, μEst, νEst, 2, 1],
    {q, 0, 1}, {b, bmin, bmax}, PlotPoints → nrsteps,
    PlotRange → {minS, maxS}, ClippingStyle → {clipcol1, clipcol2},
    ContourLines → False, ColorFunctionScaling → False,
    Contours → contourValues, ColorFunction → plotColorFunction, PlotLabel → Row[
      {Style[Subscript["m", "e"], Italic], "=2, ", Style["P", Italic], "=1"}]];
cp6 = ContourPlot[S[b, f1val, f2val, q, LEst, μEst, νEst, 3, 1],
    {q, 0, 1}, {b, bmin, bmax}, PlotPoints → nrsteps,
    PlotRange → {minS, maxS}, ClippingStyle → {clipcol1, clipcol2},
    ContourLines → False, ColorFunctionScaling -> False,
    Contours → contourValues, ColorFunction → plotColorFunction, PlotLabel → Row[
      {Style[Subscript["m", "e"], Italic], "=3, ", Style["P", Italic], "=1"}]];
(* calculate contours with critical b for low S=
 0.01 & show them as a white line *)
lp1a = Plot[critbminS[f1val, f2val, q, LEst, μEst, νEst, 1, 0.1 , minSval],
    {q, 0, 1}, PlotStyle → {White, Thickness[0.004]},
    PlotPoints → nrsteps * 10, PlotRange → {{0, 1}, {bmin, bmax}}];
lp2a = Plot[critbminS[f1val, f2val, q, LEst, μEst, νEst, 2, 0.1 , minSval],
    {q, 0, 1}, PlotStyle → {White, Thickness[0.004]},
    PlotPoints → nrsteps * 10, PlotRange → {{0, 1}, {bmin, bmax}}];
lp3a = Plot[critbminS[f1val, f2val, q, LEst, μEst, νEst, 3, 0.1 , minSval],
    {q, 0, 1}, PlotStyle → {White, Thickness[0.004]},
    PlotPoints → nrsteps * 10, PlotRange → {{0, 1}, {bmin, bmax}}];
lp4a = Plot[critbminS[f1val, f2val, q, LEst, μEst, νEst, 1, 1 , minSval], {q, 0, 1},
    PlotStyle → {White, Thickness[0.004]},
    PlotPoints → nrsteps * 10, PlotRange → {{0, 1}, {bmin, bmax}}];
lp5a = Plot[critbminS[f1val, f2val, q, LEst, μEst, νEst, 2, 1 , minSval],
    {q, 0, 1}, PlotStyle → {White, Thickness[0.004]},
    PlotPoints → nrsteps * 10, PlotRange → {{0, 1}, {bmin, bmax}}];
lp6a = Plot[critbminS[f1val, f2val, q, LEst, μEst, νEst, 3, 1 , minSval], {q, 0, 1},
    PlotStyle → {White, Thickness[0.004]},
    PlotPoints → nrsteps * 10, PlotRange → {{0, 1}, {bmin, bmax}}];
(* add empirically measured values for P. gallicus as a + on the plot *)
pointPgallicus = ListPlot[{{qEst, bEst}},
```

```
   PlotMarkers → Style["+", {Orange, Bold, FontSize → 10}], PlotLabel → None ];
linebPgallicus = Plot[bEst, {q, 0, 1}, PlotStyle → {Orange, Thickness[0.008]}];
gg = GraphicsGrid[ {{
      Show[cp1, lp1a, linebPgallicus, pointPgallicus],
      Show[cp2, lp2a, linebPgallicus, pointPgallicus],
      Show[cp3, lp3a, linebPgallicus, pointPgallicus]}, {
      Show[cp4, lp4a, linebPgallicus, pointPgallicus],
      Show[cp5, lp5a, linebPgallicus, pointPgallicus],
      Show[cp6, lp6a, linebPgallicus, pointPgallicus]}} ,
   ImageSize → 800];
legend = BarLegend[{legendColorFunction, {minSlegend, maxSlegend}},
   LegendLabel → Style["S", Italic, FontSize → 12],
   "StyledContours" → {{minSval, Directive[White]}}];
multipanel2 =
 Row[{Labeled[gg, {Row[{"Per capita productivity (", Style["b", Italic], ")"}],

     Row[{"Switching point (", Style["q", Italic], ")"}]}, {Left, Bottom},
    LabelStyle → Directive[FontFamily → "Helvetica"], RotateLabel → True,
    ImageSize → 800], Spacer[10], legend}, Alignment → Right]
```

Out[200]=

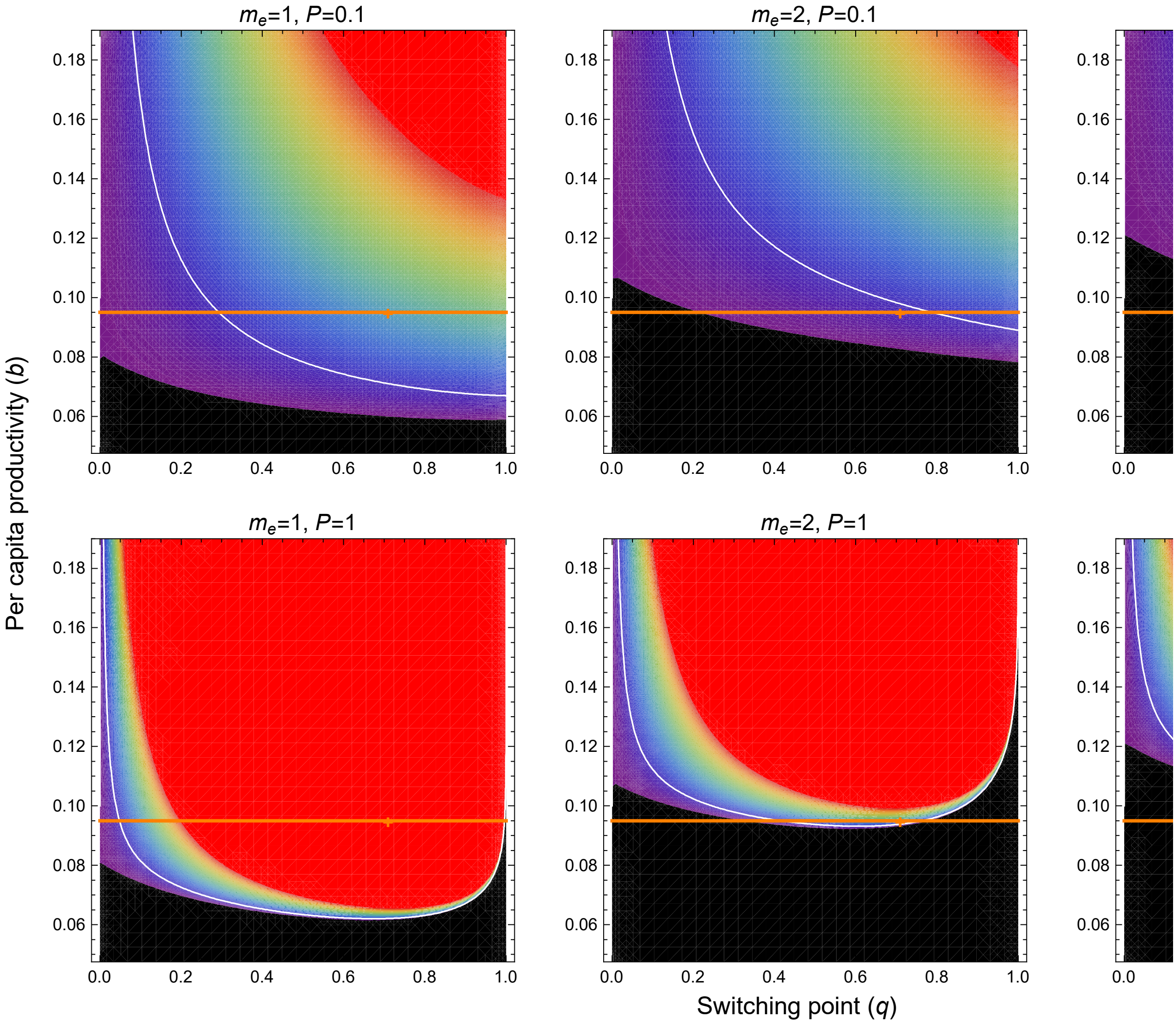

3. References

[1] Pardi, L. Studio dell’attività e della divisione di lavoro in una società di Polistes gallicus L. dopo la comparsa delle operaie (Richerche sui Polistini XII). Arch. Zool. Ital. 36, 363-431 (1951).
[2] Strassmann, J. E. et al. Worker interests and male production in Polistes gallicus, a Mediterranean social wasp. J. Evol. Biol. 16, 254-259 (2003).
[3] Southon, R. J., Bell, E. F., Graystock, P. & Sumner, S. Long live the wasp: adult longevity in captive colonies of the eusocial paper wasp Polistes canadensis (L.). PeerJ 3, e848 (2015).
[4] Pardi, L. (1948). Dominance order in Polistes wasps. Physiological Zoology, 21(1), 1-13.
[5] Jeanne, R. L., Loope, K. J., Bouwma, A. M., Nordheim, E. V. & Smith, M. L. Five decades of misunderstanding in the social Hymenoptera: a review and meta-analysis of Michener’s paradox. Biol. Rev. 97, 1559-1611 (2022).
[6] Inagawa, K., Kojima, J., Sayama, K. & Tsuchida, K. Colony productivity of the paper wasp Polistes snelleni: comparison between cool-temperate and warm-temperate
[7]Suzuki, T. Flesh intake and production of offspring in colonies of Polistes chinensis antennalis (Hymenoptera, Vespidae). II. Flesh intake and production of reproductives. Kontyu 49, 283-301 (1981).
[8] Shreeves, G., Cant, M. A., Bolton, A. & Field, J. Insurance-based advantages for subordinate co-foundresses in a temperate paper wasp. Proc. Royal Soc. B 270, 1617-1622 (2003).
[9] Nonacs, P. (2001, January). A life-history approach to group living and social contracts between individuals. In Annales Zoologici Fennici (pp. 239-254). Finnish Zoological and Botanical Publishing Board.
[10] Leadbeater, E., Carruthers, J. M., Green, J. P., Rosser, N. S. & Field, J. Nest inheritance is the missing source of direct fitness in a primitively eusocial insect. Science 333, 874-876 (2011)
[11] Michener, C. D. Reproductive efficiency in relation to colony size in hymenopterous societies. Insectes Soc. 11, 317-341 (1964).
[12] Bulmer, M. Theoretical evolutionary ecology. (Sinauer Associates, 1994).
[13] Olejarz, J. W., Allen, B., Veller, C. & Nowak, M. A. The evolution of non-reproductive workers in insect colonies with haplodiploid genetics. eLife 4, e08918 (2015).
[14] Cole, B. J. (1984). Colony efficiency and the reproductivity effect in Leptothorax allardycei (Mann). Insectes Sociaux, 31(4), 403-407.
[15] Zayed, A. Effective population size in Hymenoptera with complementary sex determination. Heredity 93, 627-630 (2004).
[16] Dyson, C. J. et al. Temporal analysis of effective population size and mating system in a social wasp. The Journal of Heredity 112, 626-634 (2021).
